# Examining the effect of post-depositional processes on the preservation and identification of stone tool residues from temperate environments: An experimental approach

**DOI:** 10.1371/journal.pone.0309060

**Published:** 2024-10-21

**Authors:** Dries Cnuts, Veerle Rots

**Affiliations:** 1 TraceoLab, University of Liège, Liège, Belgium; 2 F.R.S.—FNRS (Fonds de la Recherche Scientifique), Bruxelles, Belgium; Universita degli Studi di Ferrara, ITALY

## Abstract

Studying taphonomy is crucial for understanding how post-depositional processes impact archaeological remains. This knowledge is pivotal for accurately interpreting the archaeological record. Although taphonomy has a long tradition in archaeology, it is less developed in the analysis of stone tool residues compared to other subdisciplines. To address this gap, our study aims to further develop our understanding of the preservation potential of stone tool residues in temperate environments through actualist experiments. To achieve this, we develop a multidimensional experimental program that features the first biweekly monitoring of weathering processes on residues over a one-year cycle, aiming to understand the short-term effects of weathering immediately after tool discard. Additionally, the program involves the study of longer-term burial and weathering visual effects on different residue types within various previously unexplored depositional environments. This approach allows us to observe the visual effects of both weathering and burial processes and to improve our understanding of the different mechanisms involved in the diagenesis of stone tool residues. While known factors such as microbial activity and soil acidity play a primary role in residue decay, specific stone tool-related factors also prove important, underscoring the need to develop further a specific branch of taphonomy related to stone tool residues. Moreover, our results show that certain residue types may survive within these environments that are often considered as being hostile. A residue analysis of stone tools from temperate contexts may thus contribute unique data that can improve our understanding of past human behaviour. Future research with more diverse residue types and depositional conditions will permit further refinement of our understanding of how taphonomy affects residue preservation and enhance the reliability of residue identifications. As such, stone tool residue analysis will become firmly rooted within broader functional approaches to address how humans use stone tools and how this affects stone tool variability.

## Introduction

The study of the effect of post-depositional processes on archaeological material takes an important place in archaeology, as it is considered crucial for interpreting the archaeological record. Efremov [1] coined the scientific term taphonomy, which was initially developed as a subdiscipline of palaeontology to study the transition of animal remains from the biosphere into the lithosphere. Much later, taphonomy was introduced in archaeology to study the effect of post-depositional processes on archaeological material, including human and natural agencies [2–5]. Originally, the primary emphasis in archaeology was to study the impact at a macroscopic scale, either for individual artefacts or entire assemblages [6, 7]. Over the last three decades, the focus has expanded to investigating alterations caused by post-depositional processes on micro to nanoscale [8–12]. Within this framework, significant attention has been devoted to the role of the depositional environment in the diagenesis of archaeological remains [9, 13–16]. This microscale approach has greatly enhanced our understanding of the degradation mechanisms that lead to the disappearance or transformation of materials within the archaeological record [9].

Although studies have shown that post-depositional processes can significantly affect archaeological remains, the methods used to study these effects vary depending on the type of archaeological material involved. Furthermore, the emphasis placed on taphonomy varies significantly across different disciplines. For instance, taphonomy has been extensively developed within archaeozoology [2, 5, 17–19], where it is considered essential for studying faunal remains.

In the case of lithic analysis, the effect of post-depositional processes on lithic material has been studied less systematically. Most studies on lithic taphonomy have looked at the effect of post-depositional processes to evaluate the integrity of a specific assemblage or site rather than developing a taphonomic perspective that leads to a more accurate interpretation of cultural remains. Some of the approaches used to study the effect of post-depositional processing within lithic analysis include a descriptive analysis of the post-depositional modifications on lithics [20–23], refitting of lithic assemblages [24–27] or the study of artefact size distribution within assemblages [28–30] and the use of statistical and spatial data [31, 32]. Within use-wear analysis, the effect of post-depositional processes on use-wear has similarly not been studied systematically. Instead, specific issues related to the effect of post-depositional processes have been addressed exclusively through experimentation, such as the effect of mechanical [33] or chemical alteration [34, 35], freeze-thaw processes [36], UV-light [37] or excavation procedures [38].

Although the importance of taphonomy when analysing stone tool residues was acknowledged early on [39–41], relatively little attention has been devoted to studying the effects of post-depositional processes on stone tool residues [42, 43]. Consequently, our current understanding of residue preservation may exhibit inherent biases, culminating in an overly simplistic model that inadequately captures the intricate complexities of residue preservation. Throughout the years, three general issues have been addressed: determining the preservation potential of different residue types in particular depositional environments [42, 44, 45], evaluating the visual and chemical effect of post-depositional processes on cultural residues, often with the use of chemical solutions [41, 46, 47], and detecting the deposition of post-depositional residues [48, 49].

One of the main approaches to studying taphonomy besides analysing archaeological material is experimentation, as it has proven vital in comprehending the impact of post-depositional processes on archaeological material [50, 51]. However, experimental replication of the intricate interplay between diverse post-depositional processes and the extended duration of exposure is challenging [52]. Ideally, it is associated with a taphonomic analysis of archaeological material and advanced modelling to explore diagenetic trajectories [10, 53]. In the case of residue analysis, experiments have played a major role in understanding the short-term effect of post-depositional processes on residues. At the same time, the study of archaeological material proved informative for understanding the effect of long-term processes.

Past experiments primarily focused on the short-term effect of weathering or burial processes on residues, often related to specific environments such as dry rock shelters [42]. Langejans [32] observed that residues were preserved better on tools buried or placed in sheltered areas than on tools buried or placed in an open-air environment. Based on her observations, Langejans [42] suggested that the dry setting of caves and rock shelters inhibits microbial decay, concluding that microbial activity is mainly responsible for residue degradation. She considered other variables, such as soil pH, temperature, and burial time, less important. These observations have led to the hypothesis that residues seldom endure on stone tools from temperate environments as per the *Kopper Geigen* classification Cfb (warm temperate, no dry season, warm summer), a notion initially posited by Langejans [32]. Subsequently, few residue studies have been performed in such settings, resulting in limited opportunities for further verification. Whether such settings can preserve at least certain residue types has not yet been conclusively verified. One of the major outcomes of previous experiments is that distinctive preservation patterns may exist for different residue types. Langejans [32] observed that wood and starch residues were preserved in larger quantities than bone and muscle tissue residues, suggesting that the former types have a higher chance of survival. The burial of stone tools into a compost heap for 63 days [54] led to similar conclusions, as plant residues proved more resistant than animal residues. Another more extensive taphonomic experiment was conducted in a temperate environment under three different burial conditions—acidic peat, slightly acidic clay soils, and slightly alkaline calcareous uncompacted soil—[44]. Resin and ochre were identified as the most resistant residues, followed by keratinized remains (i.e., bird feathers, squirrel hair), softwood tracheids, and reed plant cells. Several experimental studies [42, 55, 56] have demonstrated the poor preservation potential of certain protein-rich residue types, such as meat and blood. These conclusions strongly contradict the results of many residue studies that report on the preservation of these vulnerable protein-rich residue types [57–63].

Within the field of taphonomy, a wide range of methods has been used to study the effect of post-depositional processes at different scales, ranging from the electron to the macro-level [9]. Light microscopy is commonly used in taphonomy to detect and document surface alterations at the microscopic scale [64, 65]. The integration of scanning electron microscopy (SEM) has allowed for enhanced resolution of three-dimensional structures, improved depth of field, and greater magnification compared to light microscopy [66]. Other limitations have been mitigated recently with the use of confocal microscopy [67]. Diagenetic changes at the nanoscale have been studied using transmission electron microscopy (TEM) [68], atomic force microscopy (AFM) [69], Attenuated total reflection (ATR-) Fourier-transform infrared spectroscopy (FTIR) [70–74] and internal structural changes of the material have been studied with micro-computed tomography (micro-CT) [65, 75].

Within the field of residue analysis, light microscopy has traditionally been used to observe diagenetic changes in stone tool residues [36, 37, 44, 45, 76] but it has clear limitations in detecting and accurately identifying these often-degraded residues [77, 78]. Therefore, other methods, such as scanning electron microscopy [47, 79], vibrational spectroscopic methods such as FTIR spectroscopy [56, 80–82] or Raman spectroscopy [83–85] or Gas chromatography-mass spectrometry (GC–MS) [86, 87] have been integrated within the residue analysis protocol.

In this study, we investigate the impact of temperate environments on the visual identification of stone tool residues. Our study also aims to improve the understanding of the preservation potential of these residues in temperate contexts, which have traditionally been considered unfavourable for organic preservation [12, 19, 88]. We applied a qualitative visual examination of residues using light microscopy combined with Scanning Electron Microscope Energy-Dispersive X-ray Spectroscopy (SEM-EDS). We focused on the phases after the tool was discarded, as these are considered the most destructive for archaeological remains [5]. We report on the results and implications of three experimental programs: a one-year monitoring experiment in a temperate forest environment, a three-year surface experiment in two different temperate environments, and a three-year burial experiment in four different temperate environments.

## Background: The complexity of residue taphonomy

Once residues have been deposited onto a tool’s surface, they are exposed to various post-depositional processes [3, 42]. Studies have also shown that post-depositional processes frequently deposit residues and that this may lead to misinterpretations because these residues can be mistaken for functional ones [78, 85, 89]. Residues are thus deposited through both environmental (e.g., contact with soil) and cultural (e.g., use, incidental deposition during discard) processes. Environmental residues include starch through contact with organic soils [90], fish scales from being deposited in a river environment [78], iron oxide and gypsum crystals [85], conidia [89], apatite from authigenic mineralisation [91] and bone [48]. All residues will also degrade and/or alter due to exposure to post-depositional processes. The nature and extent of these taphonomic alterations depend on the depositional environment in which the artefact is situated [92].

Different processes may be at play when a stone tool is located on the soil surface (*biostratinomic context*) or when it is buried (*burial context*) [5, 51] and the resistance against these processes varies between residue types. Insights from biochemistry and previous studies permit to hypothesise that the preservation potential of a residue depends on the resistance of its biomolecules (e.g., lipids, carbohydrates, proteins, and lignin) [93, 94] against the local conditions of the depositional environment (i.e. pH, biological activity, sediment type, etc.) [13–15, 42, 95]. Preservation is further influenced by the strength of the bonding between the residual deposit and the mineral tool surface [96, 97].

Microbial decay is considered the most frequent degradation process at archaeological sites, except for waterlogged, extremely arid, or permafrost settings. It is caused by heterotrophic microorganisms (e.g., bacteria, fungi), which secrete specific enzymes (e.g., collagenase for bone [98] to break down these complex molecules into more absorbable molecules. Since the microbial attack is dominant, some discussions have questioned why certain residual deposits survive. Haslam [99] discussed various protective mechanisms that protect starch grains from microbial decay, such as their penetration into the stone tools’ surface cracks or the presence of soil aggregates, clays, or heavy metals. A similar hypothesis has been proposed to explain the preservation of proteins and DNA [100, 101]. Controlled laboratory experiments demonstrated that certain starch types are more resistant to bacterial decay than others, which can lead to a preservation bias in the archaeological record [102]. Rapid desiccation may also protect the residue against microbial attack [103]. The fact that microbial decay has occurred can be established through the remnants of heterotrophs (e.g., fungal structures) present on the stone tools or through specific damage from fungi. Although various studies report on the presence of fungal structures in association with stone tool residues, only one study [104] used it as a proxy to assess past biological activity. In this study at Sibhudu Cave, the minimal presence of fungal structures on the stone tools was taken as an explanation for the good preservation of the organic residues [104].

Physical degradation processes (wind, water transport, freezing and thawing, and sediment transport) may also damage residual deposits [105]. Only for the Sterkfontein cave has the poor residue preservation been explained partly by the friction caused by water flow and sediment erosion [106]. Stone tool residues may be degraded by chemical processes, including hydrolysis (i.e. decomposition by the addition of water molecules), natural oxidation [107], photo-oxidation through exposure to sunlight [108], bone dissolution by acidity [88, 109, 110], and thermal degradation by combustion [76]. An often-overlooked aspect of residue taphonomy is the impact of the strength of the bond between the residues and the stone tool surface. This bond may determine the resistance of the residue to physical agents of deterioration (water, wind, frost, soil compaction and creep) [3]. The precise mechanisms of residue adherence to stone tool surfaces remain poorly understood [111]. Still, it has been suggested that the bond between the residue fragment and the stone tool surface is caused by adhesion, particularly physical adsorption, which can be attributed to Van der Waal forces [112]. Earlier publications [103] have suggested that the bonding between the residue deposit, the stone tool, and the soil particles is central to preserving residues. Rapid residue dehydration changes its ionic composition, and the chemically charged stone and soil particles form a hydrophobic, insoluble complex. It might explain why residues can withstand groundwater and microbial degradation over long periods. Barton [103] has also suggested that the variation in preservation within the same starch deposit might be explained by the bonding of the residue with the stone tool surface, which protects the inner part of the residue deposit from being attacked by the microorganisms. In residue analysis on ceramics, the strong bond between protein residues and mineral ceramic surfaces significantly impacted the extraction success rate [97, 113].

Other experimental studies have demonstrated that stone tool residues may undergo a visual or chemical transformation when exposed to post-depositional processes. Anderson [41] observed holes in the antler residues at high magnification (x6000) corresponding in diameter to collagen fibres, indicating that hydrogen peroxide destroyed the organic components, leaving only the hydroxyapatite minerals behind. Others found that the morphology of bone and bamboo residues persisted and could be identified with both incident and scanning electron microscopy [46]. The energy-dispersive analysis revealed that hydrogen peroxide had caused chemical alteration: bone had lost its potassium (K), while the proportion of iron (Fe) in bamboo had decreased. Hayes and Rots [47] showed that weak solutions of hydrochloric acid (HCl(_aq_) and sodium hydroxide (NaOH) lead to heavy degradation of certain residue types. The exposure of hydroxyapatite residues (bone, antler, and fish scales) to 3% HCl led to the complete disappearance of phosphorus. At the same time, NaOH completely removed the hydroxyapatite minerals inside bone, antler, and fish scale residues, leaving only the collagen behind [114, Fig 14]. The submersion of raw starch into NaOH resulted in swollen, gelatinized starch grains with a dramatic increase in size [114, Fig 15]. Muscle tissue has been found to lose its diagnostic traits (Defined as containing enough specific visual traits to be unambiguously identified with optical microcopy, modified after [44]) after being exposed to acidic conditions as the striated appearance of the sarcomeres disappeared, hampering the identification of these residues with incident light microscopy [44].

## Materials and methods

### Experimental design

#### One-year monitoring experiment

For this experiment, eight variables were considered (**see SI1 in S1 File; Table 1**). Five of these variables were kept constant throughout the experiment: raw material, adhesive recipe, use motion, use duration, and the environment. The three remaining variables were varied: the handle material, the worked material, and the duration of exposure.

**Table 1:** Residue variables recorded after exposure.

	*Variable*	*Categories*
*1*	Association with the used edge	absent; weak; intermediate; strong
*2*	Association with use-wear	yes; no
*3*	Degree of smearing	absent; weak; intermediate; heavy/ significant
*4*	Directionality against the used edge	perpendicular; oblique; parallel
*5*	Density	d1; d2; d3; d4
*6*	Residue loss	absent; weak; intermediate; strong
*7*	Location	--
*8*	Distribution	local; dispersed
*9*	Change in distribution	absent; weak; intermediate; strong
*10*	Interpretability Residue cause	uncertain; poor certainty; moderate certainty; high certainty; certain
*11*	Visual distinctive characteristics	yes; no
*12*	Degree of fragmentation	absent; weak; intermediate; strong
*13*	Colour	--
*14*	Change in colour	yes; no
*15*	Fungi density	d1; d2; d3; d4
*16*	Interpretability Residue Nature	uncertain; poor certainty; moderate certainty; high certainty; certain

**Fig 1:**
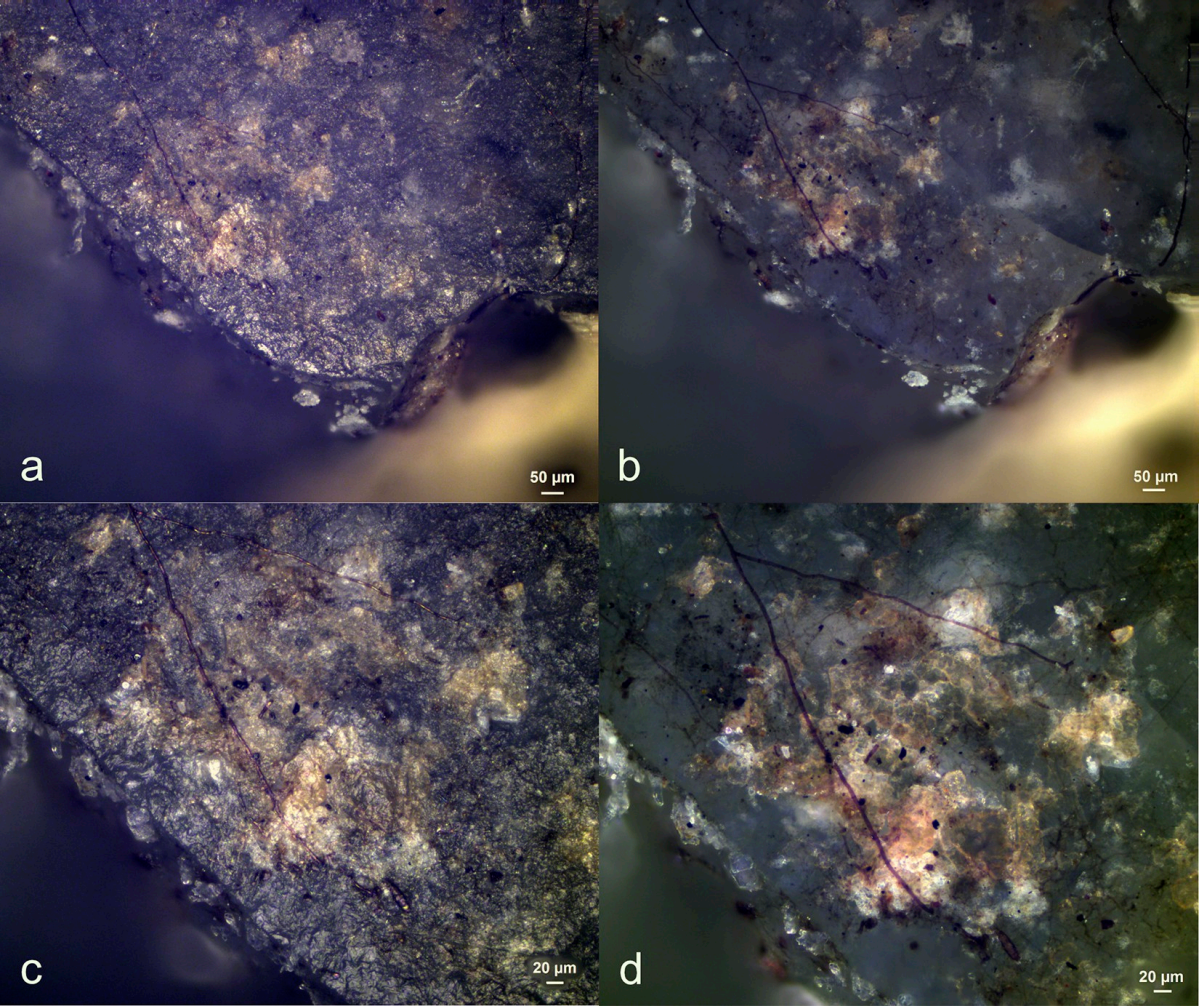
Visual characteristics of antler production residues after short-term weathering exposure (Exp49/154; 36weeks): local distribution on the butt (a+b), strong degree of smearing (d), translucent under bright field (a+b), brown discolouration under cross-polarized light (b+c), flat topography (c+d), internal cracks (d) and sharp edges (d) (a+b = x100; c+d = x200).

Fifty-two backed blades (Exp49/131-Exp49/182) were knapped out of Harmignies flint by an experienced knapper (Christian Lepers, TraceoLab). Half of the tools (N = 26) were hafted on wooden handles, while the other half were hafted on bone handles. All stone tools were hafted in a juxtaposed position, secured with a mixture of 70% natural spruce resin and 30% natural beeswax, and subsequently secured with leather bindings. The stone tools with wooden handles were utilized for removing soft animal tissues such as muscle tissue, tendons, and fat from bones, whereas the stone tools with bone handles were employed for cutting fresh wood (**see SI1 in S1 File; Fig 1**). Each stone tool was used for at least 20 minutes to ensure sufficient residue accumulation. No permits were required for the described study, which complied with all relevant regulations.

After their use, tools were immediately dehafted and deposited on the soil surface within a pine forest, with the ventral surface facing down in contact with the soil and the dorsal face being exposed to the air (**see SI1 in S1 File; Table 2**). To safeguard against carnivores, the tools were covered with pine tree branches. At two-week intervals, one animal tissue-cutting tool and one wood-cutting tool were retrieved from the surface (**see SI1 in S1 File; Table 3**) and immediately placed in a freezer at -18°C to halt all bacterial activity. Unfortunately, two stone tools used on fresh bone could not be recovered and had to be excluded from further analysis.

**Table 2:** Observed characteristics of production residues exposed to short-term weathering.

*Cause*	*Production*
*Mechanism of deposition*	Hammer
*Nature*	Antler
*N° tools with observed residual deposit*	3
***Residue cause characteristics***	
*Location*	Butt
*Residue loss*	absent
*Density*	d2
*Association with the used edge*	NA
*Degree of smearing*	strong
*Change in distribution*	No
*Directionality against the used edge*	NA
*Interpretability Cause*	High certainty
***Residue nature characteristics***	
*Distinctive characteristics*	cracks, ***sharp*** edges, flat topography (RL); Ca Ph peaks (SEM-EDS)
*Degree of fragmentation*	absent
*Colour*	translucent (brightfield), white/brown (polarizer)
*Colour change*	yes
*Fungal density*	1
*Interpretability Nature*	Poor certainty*

**Table 3:** Observed characteristics of hafting residues exposed to short-term weathering.

*Cause*	*Hafting*			
*Mechanism of deposition*	Handle		Binding	Adhesive
*Nature*	Bone	Wood	Leather	Resin
*N° tools*	22	22	44	44
*N° of tools with original residual deposit*	NA	NA	NA	44
*N° tools with observed residual deposit*	1	6	0	44
*Residue loss*	absent	absent	NA	absent
*Density*	d2	d2	NA	d4
*Association with the used edge*	NA	NA	NA	NA
*Degree of smearing*	strong	strong	NA	absent
*Change in distribution*	no	no	NA	no
*Directionality against the used edge*	absent	absent	NA	NA
*Interpretability Cause*	poor certainty	poor certainty	NA	moderate certainty
*Visual characteristics*	NA	plant cell walls, cellulose fibres	NA	Brown, viscous appearance
*Degree of fragmentation*	weak	weak	Na	absent
*Colour*	White, brown	dark brown	NA	dark brown to black
*Colour change*	no	no	NA	Yes
*Fungal density*				
*Interpretability Nature*	poor certainty*	high-certainty	NA	poor certainty

The weathering process was closely monitored at regular intervals to assess (1) residue loss, (2) possible changes in residue distribution, (3) differential residue preservation, (4) possible alteration to visual characteristics for different residue types, and (5) the role of variables such as exposure time, stone tool orientation, and physical and biological weathering agents.

#### Three-year surface experiment

For this experiment, nine experimental variables were considered (**see SI1 in S1 File; Table 4**). Five of these variables were kept constant throughout the experiment: raw material, adhesive recipe, use motion, use duration, and exposure time. The four remaining variables were varied: prehension mode or handle material, worked material, and depositional environment.

**Table 4:** Observed characteristics for use residues exposed to short-term weathering.

*Cause*	*Use*		
*Mechanism of deposition*	Material worked		
*Nature*	Animal tissue	Bone	Wood
*N° tools*	22	NA	22
*N° tools with residual deposit*	13	14	22
*Residue loss*	strong	intermediate	intermediate
*Density*	d2	d2	d3
*Association with the used edge*	weak	strong	strong
*Degree of smearing*	intermediate	strong	strong
*Change in distribution*	No	No	No
*Directionality against the used edge*	absent	strong	strong
*Interpretability Cause*	poor certainty	moderate certainty	certain
*Visual characteristics*	Dorsal: Red, brown Ventral: Translucent	cracks, sharp edges, flat topography	cell walls
*Degree of fragmentation*	strong	intermediate	strong
*Colour*	Black	Yellow/dark brown	dark brown
*Colour change*	Yes	Yes	Yes
*Fungal density*	2	2	3
*Interpretability Nature*	poor certainty	moderate certainty	Moderate certainty

Sixteen scrapers (**see SI1 in S1 File; Table 5**) were knapped out of Harmignies flint by an experienced knapper (Christian Lepers, TraceoLab). Thirteen stone tools were produced using an antler hammer, while the remaining three were created using a wooden hammer. Four tools were hafted on a bone handle and four other tools on a wooden handle, in each case with leather as a binder. Additionally, one tool was secured using a resin and beeswax adhesive. All scrapers (**see SI1 in S1 File; Table 5**) were utilized for a 20-minute scraping activity on a specific material. The materials included hardwood (*Acer platanoides*), soft plants (*Raphanus sativus longipinnatus*, i.e. root vegetable), and dry or fresh bone. After their use, tools were immediately dehafted. No permits were required for the described study, which complied with all relevant regulations.

**Table 5:** Observed characteristics for production residues after exposition to weathering for three years.

*Cause*	*Production*	
*Mechanism of deposition*	Hammer	
*Nature*	Antler	Wood
*N° tools*	13	3
*N° tools with residual deposit*	1	0
*Residue loss*	weak	NA
*Density*	2	NA
*Association with the used edge*	NA	NA
*Degree of smearing*	strong	NA
*Change in distribution*	No	NA
*Directionality against the used edge*	NA	NA
*Interpretability Cause*	certain	NA
*Visual characteristics*	Under cross-polarized light: Brown, cracks, sharp edges, flat	NA
*Degree of fragmentation*	absent	NA
*Colour*	Translucent (bright field); brown (cross-polarized light)	NA
*Colour change*	Yes	NA
*Interpretability Nature*	poor certainty	NA

**Fig 2:**
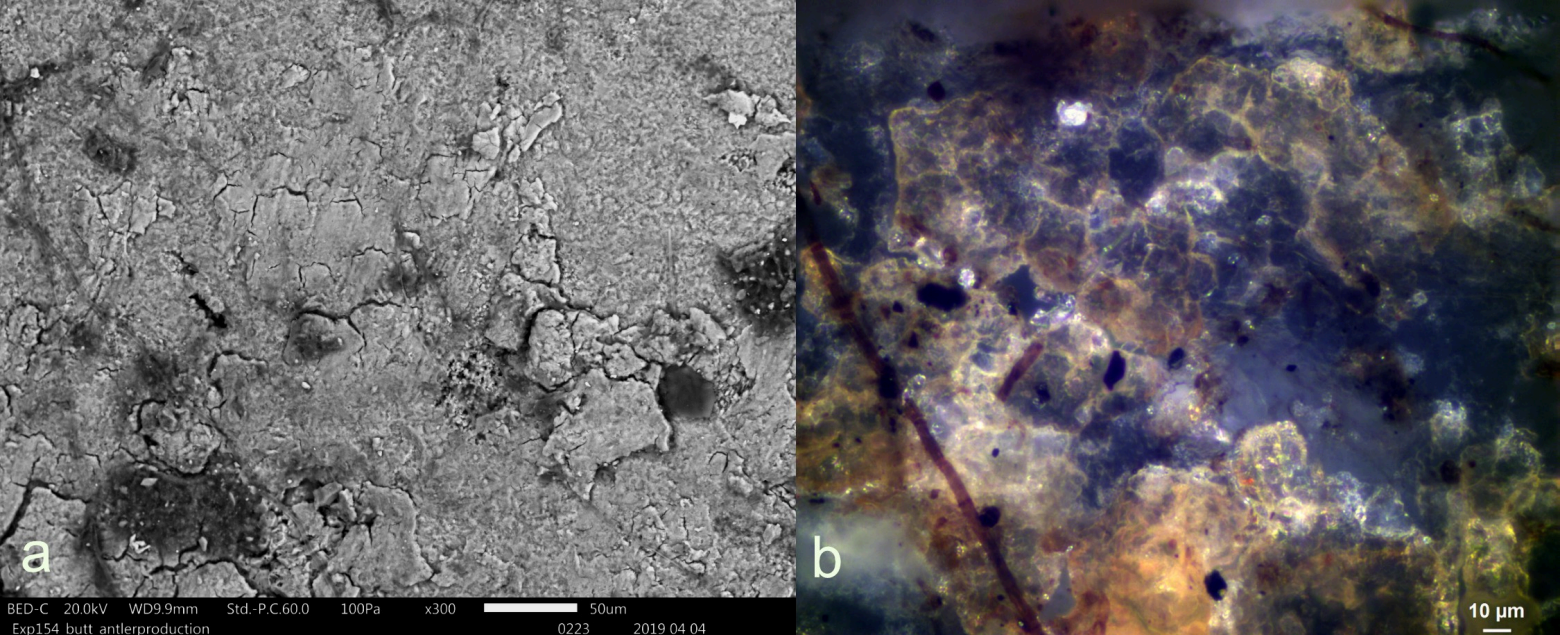
a) antler production residue after 36 weeks displaying a significant degree of smearing b) EDS spectrum of antler residue with characteristic calcium and phosphorus peaks (a = x300).

Eight scrapers were deposited on the surface of a pine forest in Rochefort (Belgium), while the other scrapers were placed on the surface of a mixed wood forest in Lommel (Belgium) (**see SI1 in S1 File; Fig 2**). All tools were placed with the ventral surface facing down in contact with the soil and the dorsal surface upwards, exposed to the air. To minimize potential disturbances from animals or humans, branches were placed over the stone tools. Notable variations between the two environments include differences in the topsoil’s acidity and the sediment type (**see SI1 in S1 File; Table 6**).

**Table 6:** Observed characteristics of hafting residues exposed to weathering for three years.

*Cause*	*Hafting*			
*Mechanism of deposition*	Handle		Binding	Adhesive
*Nature*	Bone	Wood	Leather	Resin
*N° tools*	4	4	8	1
*N° tools with residual deposit*	0	0	0	1
*Residue loss*	NA	NA	NA	absent
*Density*	NA	NA	NA	4
*Association with the used edge*	NA	NA	NA	NA
*Degree of smearing*	NA	NA	NA	absent
*Change in distribution*	NA	NA	NA	No
*Directionality against the used edge*	NA	NA	NA	NA
*Interpretability Cause*	NA	NA	NA	moderate certainty
*Visual characteristics*	NA	NA	Brown, viscous appearance	NA
*Degree of fragmentation*	NA	NA	absent	NA
*Colour*	NA	NA	dark brown	NA
*colour change*	NA	NA	Yes	NA
*Interpretability Nature*	NA	NA	poor certainty	NA

#### Burial experiment

In this experiment, eight experimental variables were considered significant (**see SI1 in S1 File; Table 7**). The experiment kept three variables constant: raw material, use motion, and use duration. The five remaining variables were varied: the worked material, prehensile mode, handle material, adhesive application, and the tool user.

**Table 7:** Observed characteristics for use residues exposed to weathering for three years.

Cause	Use			
*Mechanism of deposition*	Material worked			
*Nature*	Bone	Fat	Plant	Wood
*N° tools*	4	4	4	4
*N° tools with residual deposit*	1	4	2	2
*Residue loss*	strong	strong	Intermediate/strong	strong
*Average Density*	1,5	1	2	1
*Association with the used edge*	strong	intermediate	weak	strong
*Degree of smearing*	weak	weak	intermediate	intermediate
*Change in distribution*	No	No	No	No
*Directionality against the used edge*	parallel	Absent/perpendicular	Absent/perpendicular	Absent/perpendicular
*Interpretability Cause*	high certainty	moderate certainty	moderate certainty	poor certainty
*Visual characteristics*	Under cross-polarized light: Brown, cracks, sharp edges, flat	Dorsal: Red, brown Ventral: Translucent	Cell walls, cellulose fibres	No
*Degree of fragmentation*	strong	intermediate	intermediate	strong
*Colour*	translucent/brown	translucent/brown	Yellow/dark brown	dark brown/black
*Colour change*	Yes	Yes, partially	Yes	Yes
*Interpretability Nature*	poor certainty	poor certainty	certain	uncertain

Twenty-scrapers scrapers (**see SI1 in S1 File; Table 8**) were knapped out of Harmignies flint by an experienced knapper (Christian Lepers, TraceoLab). Among the seventeen hafted tools, nine were hafted on a bone handle and eight on a wooden handle, utilizing leather as a binder. Additionally, twelve tools were secured with a resin and beeswax adhesive. The twenty-six scrapers (**see SI1 in S1 File; Table 8**) were employed to scrape various materials, including hardwood (*Acer platanoides*), soft plant (*Raphanus sativus longipinnatus*), dry bone, or fresh hide, for 20 minutes. After their use, tools were immediately dehafted. No permits were required for the described study, which complied with all relevant regulations.

**Table 8:** Observed characteristics for production residues after three-year burial.

*Cause*	*Production*	
*Mechanism of deposition*	Hammer	
*Nature*	Antler	Wood
*N° tools*	28	7
*N° tools with residual deposit*	1	0
*Residue loss*	NA	NA
*Average density*	d2	NA
*Association with the used edge*	NA	NA
*Degree of smearing*	Significant	NA
*Change in distribution*	No	NA
*Directionality against the used edge*	NA	NA
*Interpretability Cause*	High certainty	NA
*Visual characteristics*	flake-like structure	NA
*Degree of fragmentation*	weak	NA
*Colour*	white	NA
*Colour change*	no	NA
*Average fungal density*	1	NA
*Interpretability Nature*	Poor certainty	NA

All the stone tools were buried at a depth of 20 cm beneath the soil surface (**see SI1 in S1 File; Table 9**) in four distinct locations with differing environmental conditions, such as variations in vegetation, soil acidity, and sediment type (**see SI1 in S1 File; Table 10**). Five scrapers were buried at Scladina Cave as a control sample for comparing the effects of post-depositional processes between open-air and cave sites. All tools were placed with the ventral surface facing downwards towards the soil and the dorsal face upwards, exposed to the air. After three years, the stone tools were excavated, and the surrounding soil was retained to monitor organic soil constituents like rootlets.

**Table 9:** Observed characteristics for hafting residues after three-year burial.

*Cause*	*Hafting*			
*Mechanism of deposition*	Haft		Binding	Adhesive
*Nature*	bone	wood	leather	resin/beeswax
*N° tools*	9	8	17	10
*N° tools with residual deposit*	0	0	1	10
*Residue loss*	NA	NA	NA	weak
*Average density*	NA	NA	d2	d4
*Association with the used edge*	NA	NA	NA	NA
*Degree of smearing*	NA	NA	absent	absent
*Change in distribution*	NA	NA	NA	no
*Directionality against the used edge*	NA	NA	NA	NA
*Interpretability Cause*	NA	NA	High certainty	High certainty
*Visual characteristics*	NA	NA	Fibrous structure	
*Degree of fragmentation*	NA	NA	absent	absent
*Colour*	NA	NA	brown	Dark brown
*Colour change*	NA	NA	No	yes
*Average fungal density*	NA	NA	0	4
*Interpretability Nature*	NA	NA	Poor certainty	Poor certainty

**Table 10:** Observed characteristics for use residues after three-year burial.

*Cause*	*Use*			
*Mechanism of deposition*	fresh bone scraping	fresh hide scraping	tuber scraping	wood scraping
*Nature*	Bone	Fat, Hair	Plant	Wood
*N° tools*	8	6	8	6
*N° tools with a residual deposit*	4	2	3	5
*Residue loss*	Variable, depending on the depositional environment	Intermediate to strong	Intermediate to strong	Intermediate to strong
*Average density*	d2.4	d2	d2	d1.7
*Association with the used edge*	Strong	Strong	Intermediate	Strong
*Degree of smearing*	Intermediate	Strong	Weak	Absent
*Change in distribution*	No	No	No	No
*Directionality against the used edge*	perpendicular	Perpendicular	Absent	Absent (2); parallel (2) Perpendicular (1)
*Interpretability Cause*	High certainty	High certainty	Poor certainty	Moderate certainty
*Visual characteristics*	angular, flat, cracked and birefringent edges	translucent, reflective	cell walls	cell walls
*Degree of fragmentation*	Absent (2) Strong (2)	Intermediate(1) strong (1)	Weak (1) Strong (2)	Intermediate (1) Strong (5)
*Colour*	White- Yellow	Translucent	Brown	Dark brown
*Colour change*	Partially	No	yes	yes
*Average fungal density*	d0.8	d3	d2	d1.2
*Interpretability Nature*	Poor certainty*	Moderate certainty	Certain	Moderate certainty

#### Meteorological data associated with the experimental conditions

Meteorological data associated to the experiment’s duration were obtained from the Meteostat website, as this information was not collected directly from the burial environment. The data was sourced from the weather station in Uccle, Belgium, covering the period from July 8, 2014, to November 22, 2017 (1234 days). The station is situated at an elevation of 28 meters, with coordinates 50.8505, 4.3488 (source: Meteostat.net).

**Fig 3:**
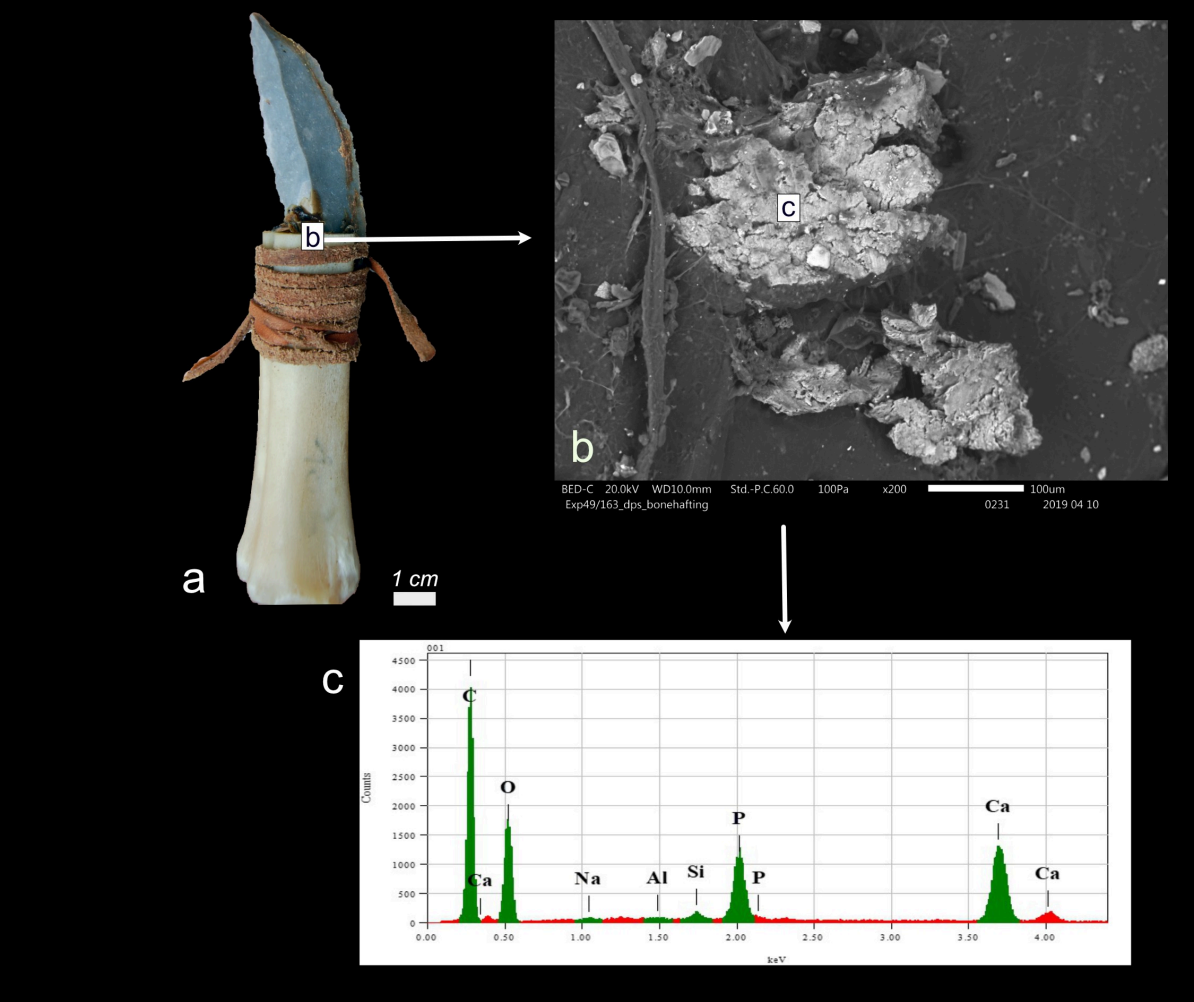
Possible bone hafting residue after four weeks of weathering a) Tool Exp49/163 b) possible bone shaft residue embedded in resin and beeswax mixture on tool EXP49/163 c) EDS analysis of the possible bone residue, indicating Calcium and Phosphorus peaks(b = x160).

**Fig 4:**
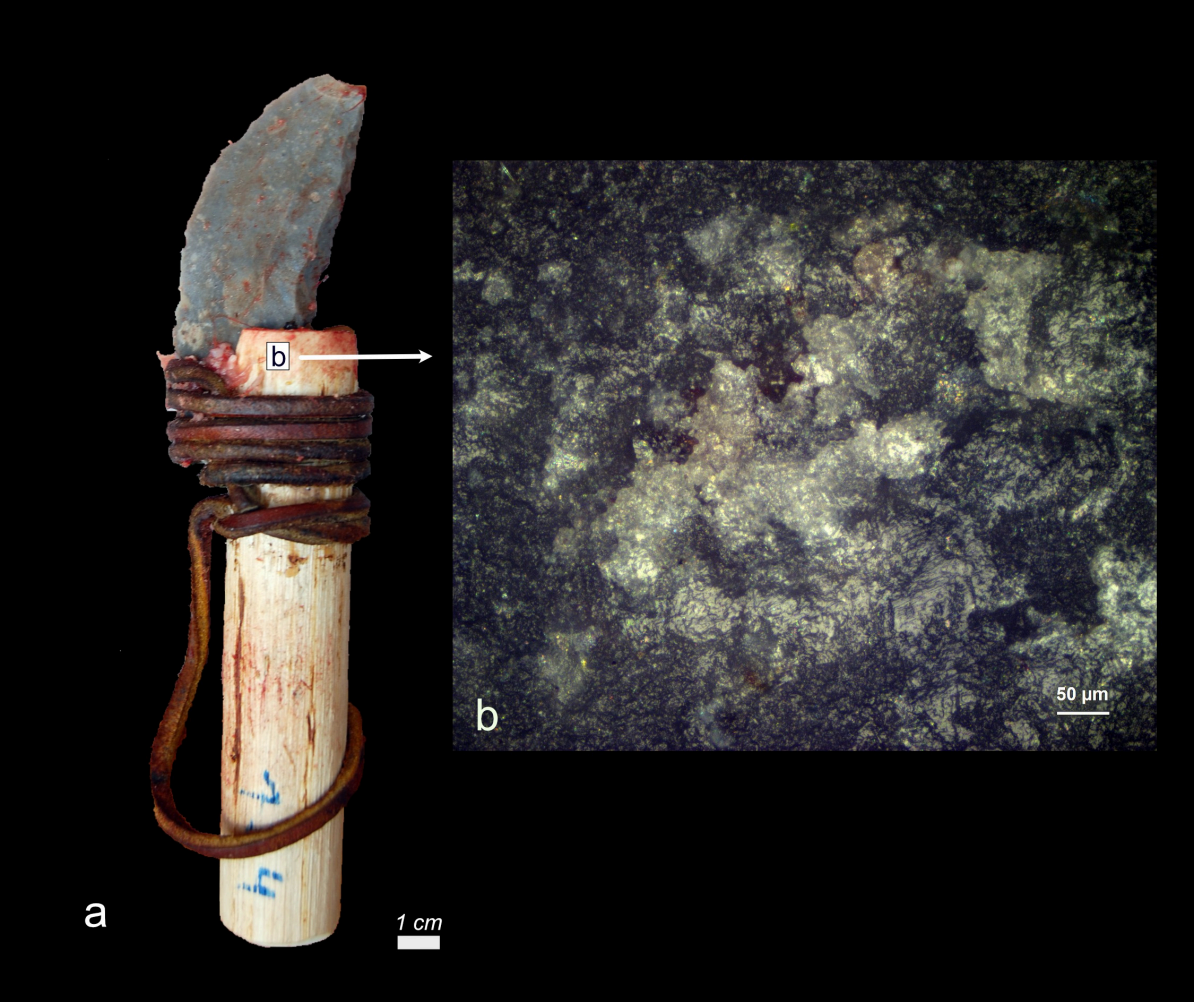
Wood hafting residue after two weeks of weathering a) Tool Exp49/165b) wood haft residue on right ventral surface characterised by a significant degree of smearing (b = x200).

The average temperature was 11.21°C, with recorded temperatures ranging from a minimum of -7.0°C to a maximum of 33.1°C (**See S1 in S1 File; Fig 3**). The average daily precipitation was 3.47 mm, with a maximum daily precipitation of 42.9 mm. The average wind direction was 199.02°, and the average wind speed was 12.29 km/h. The average atmospheric pressure was 1016.76 hPa (For detailed information daily meteorological data see **S1 in S1 File; Fig 4** and **S2 in S1 File**).

### Analytical protocol

Sixteen characteristics were recorded to investigate the impact of weathering or burial on each residual deposit (Table 1). Before and after use, all tools were photographed to document any visible residue accumulation at a macroscopic level. Stereomicroscopy was utilized to examine the overall patterns of residue and to correlate these deposits with the artefacts’ typological and technological attributes. However, its limited magnification does not allow the identification of stone tool residues. High magnification reflected light microscopy (100x–1000x) was primarily used, leveraging illumination methods such as cross-polarisation to highlight distinctive features of certain residue types like plant cell walls [44, 77]. The residues present on the experimental stone tools were therefore examined using a range of microscopy techniques, including a Zeiss stereomicroscope Discovery V.12 (with magnifications up to ×120), a Zeiss Macro-Zoom microscope V.16 (with magnifications up to ×180) and a Zeiss Imager metallurgical incident light microscope (with magnification ranging from ×50 to ×1000), equipped with rotating polarizers and differential interference contrast (DIC).

SEM-EDS allows for higher magnification and facilitates the elemental analysis of residues, which is particularly advantageous for observing smaller features or characterizing residues with no visually distinctive traits [47, 79, 115, 116]. However, SEM-EDS does not provide molecular information, which is often necessary to confirm the exact nature of a residue. When SEM-EDS is applied to hard animal residues such as bone and antler, it identifies calcium and phosphorus elements separately and not the bone mineral apatite (a form of calcium phosphate that provides structural integrity to bones and teeth) [47, 116, 117]. Therefore, additional visual characteristics—such as smearing, the used edge, and the location—were considered to distinguish functional osseous residues from calcium or phosphorus material residues that may originate from sediment or the cortex, as described in [91, 118]. In general, it can be stated that the elemental composition provides a preliminary indication of the residue’s nature and guides further analysis [79]. Therefore, certain residues were analysed in more detail with a JEOL IT300 SEM-EDS (EDS detector JEOL ex-230). Images and elemental spectra of the residues were acquired in situ on the tool surface in low vacuum (LV) mode (100Pa) using the backscattered electron detector (BED) at 20.0kV with a probe current (PC) of 60.0.

## Results

### One-year monitoring experiment

#### Impact on the recognition of stone tool residues and their deposition processes

Three antler production residues (Exp49/145, Exp49/154, and Exp49/159) were meticulously examined, and it became evident that weathering had a negligible impact on these residual deposits. These three remnants exhibited a flat topography, internal cracks, and well-defined sharp edges. The original white colour had transformed into a translucent or brown hue, which became most apparent under cross-polarizing light (**Fig 1**). An SEM-EDS analysis (as illustrated in **Fig 2**) revealed significant peaks for calcium and phosphorus, suggesting that elemental composition of the antler production residues remained intact. Only a minimal presence of fungal spores was noted, likely attributed to the residue’s isolated distribution on the tool’s butt.

The diagnostic criteria for identifying organic hammer residues were consistently applicable across all four cases, as outlined in **Table 2**. These criteria included their localized distribution on the butt, the pronounced degree of smearing, and the perpendicular orientation relative to the ventral surface, collectively facilitating their precise identification.

A small, restricted patch of white residue (EXP49/163), firmly affixed to the resin and beeswax mixture on the tool’s inactive surface, was detected. The elemental characterisation through SEM-EDS analysis revealed Calcium and Phosphorus peaks (**Fig 3**). The elemental composition, combined with its visual characteristics (weak degree of smearing) and its location on the stone tool (non-active region), suggests deposition through friction with a bone shaft. However, the minimal visual clues, such as the absence of a clear smearing pattern, and the limitations of EDS in identifying bone mineral, resulted in a low degree of certainty in the interpretation (**Table 3**).

*Six wood haft residual deposits* could be recognized, but only two deposits [EXP49/151; EXP49/165) could be identified with certainty as they display a significant degree of smearing (**Fig 4**). These residues are further identified by their tendency to compress the internal structure of the wood tissue, making it challenging to discern intact plant cells. This sets them apart from the environmental wood residues we observed, which lack this degree of smudging. In the latter cases, the wood tissue remains unaltered, with plant cell walls being distinctly visible in most instances.

**Fig 5:**
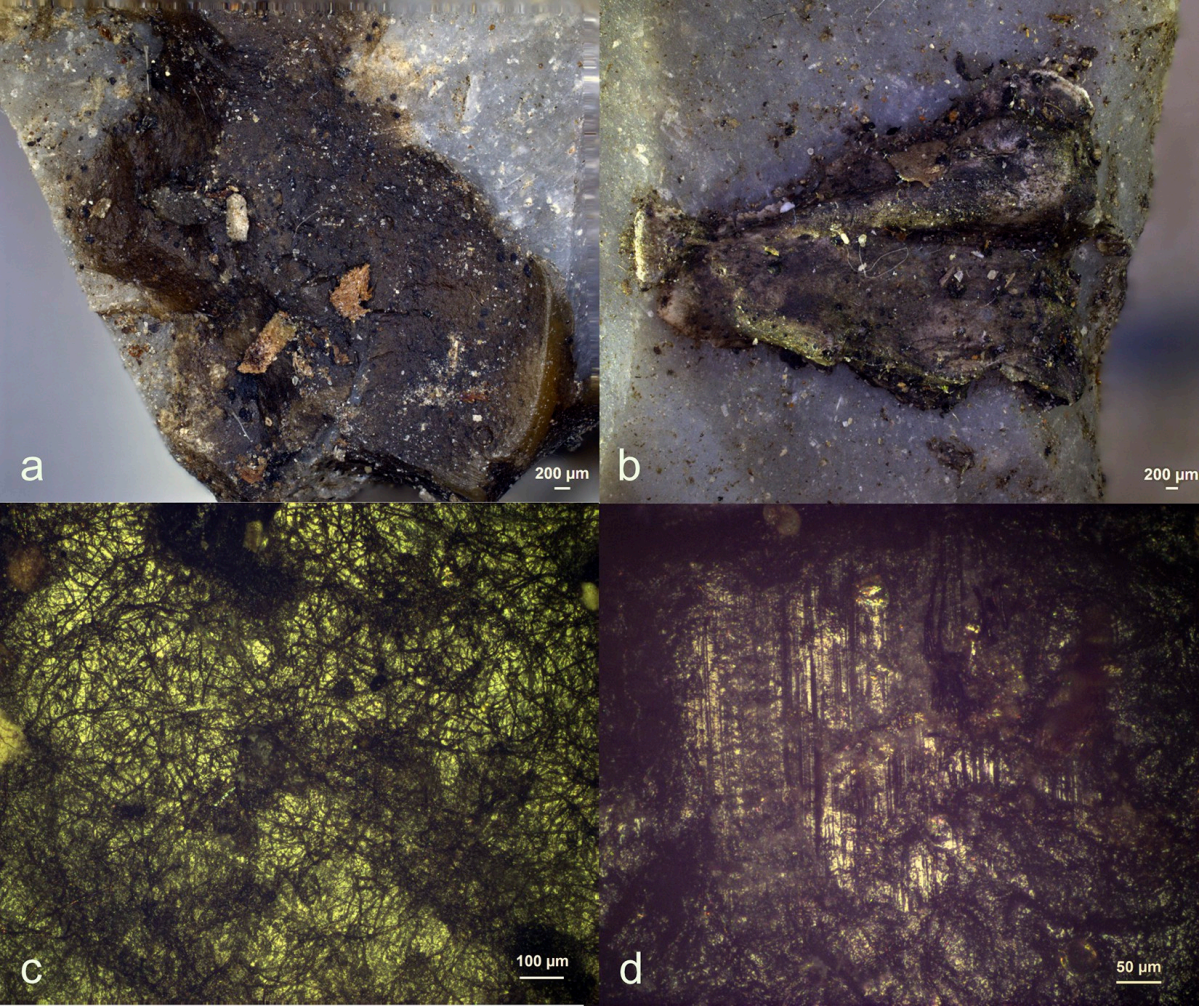
Visual characteristics of resin and beeswax residues after short-term weathering exposure: Brown discolouration (a+b+c+d), high fungal density (c), striations from hafting (d). (a = Exp49/132; 34 weeks), (b = Exp49/137; 30 weeks), (c = Exp49/178; 44 weeks), (d = Exp49/134, 10 weeks) (a = x21.5; b = x107; c = x100; d = x200).

While all forty-four stone tools were secured with leather bindings, no traces of binding residues were discernible. All eighty-six adhesive deposits (comprising resin and beeswax) remained completely intact, both in distribution and density, as illustrated in **Fig 5**. This resilience to fungal decay was particularly striking, as the fungal growth was so rampant on certain adhesive residues that they assumed a dark, almost black appearance, which sometimes made visual identification challenging.

**Fig 6:**
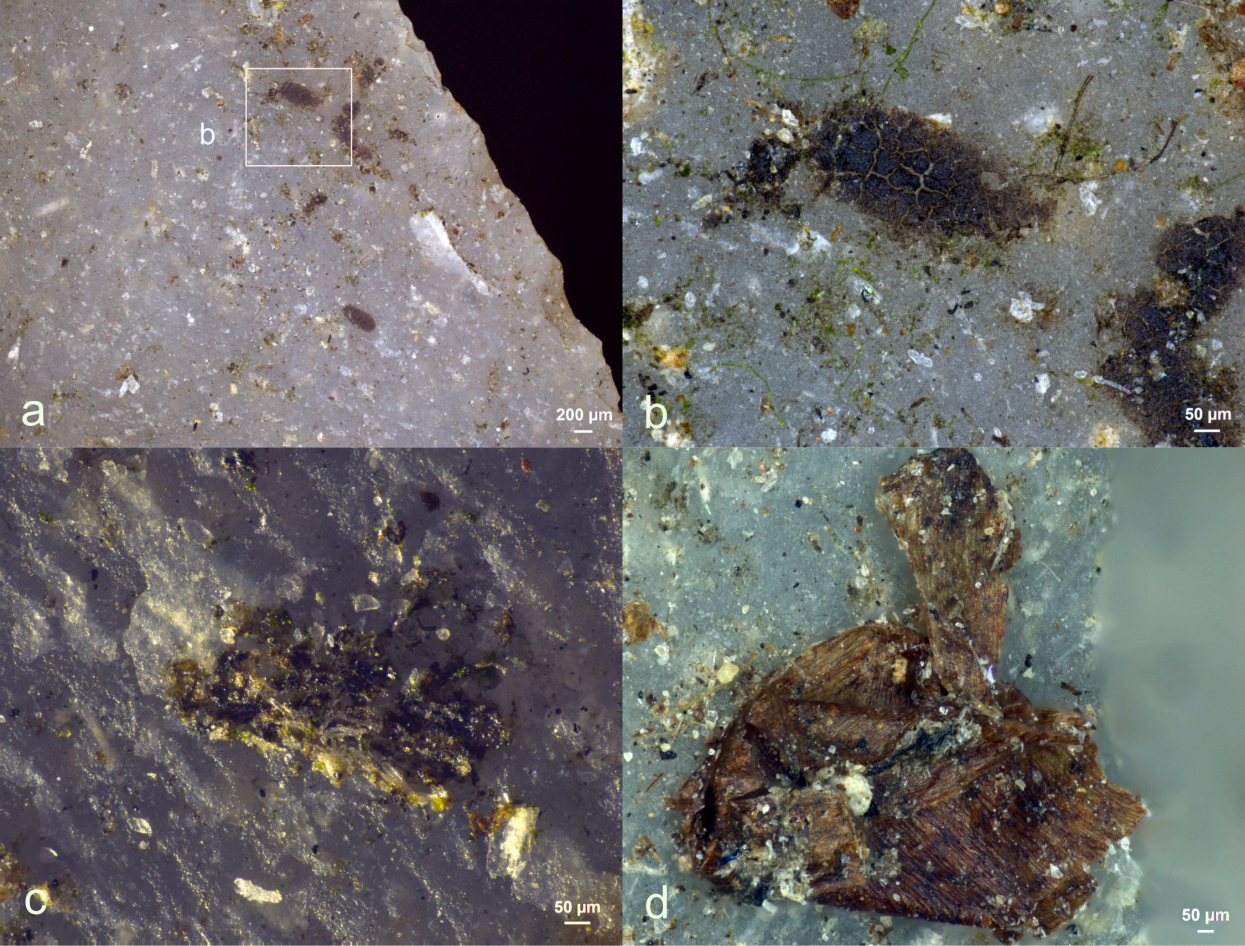
Visual characteristics of butchering residues after short-term weathering exposure: Strong loss leading to poor densities (a), weak association with the used edge (a), black discolouration of blood (a + b), amorphous appearance (c+d), and blood cells only visible under transmitted light (e + f) (a+b+c = EXP49/138; 38 weeks), (d+e+f = EXP49/137; 30 weeks)–(f = stained with Safranin O) (a = x107; b = x180; c = x180; d = x107; e = x400; f = x400).

Soft animal use residues, comprising muscle tissue, fat, and red blood cells, were identified on nineteen recovered meat processing tools (soft animal residues refer to the remains or by-products that originate from the soft tissues of animals. These soft tissues include muscles, organs, skin, and other non-bony parts. They often possess few visual characteristics [44]). The soft animal tissue deposits transformed on the majority of these tools (N13), shifting from thick and continuous depositions (density 4) to isolated fragments (density 1). Five of the remaining six tools exhibited residue densities of two, while one had a density of three. Detecting these diminished deposits necessitated using an incident light microscope, and their altered appearance posed a challenge for identification (as demonstrated in **Fig 6**).

A few residual deposits possessed distinctive characteristics that facilitated their recognition as resulting from use. These features included the pronounced association of specific blood residues with the utilized edge and significant fat smearing, which was only observable through scanning electron microscopy. The weathering process substantially reduced the chances of attributing these soft animal residues to their original cause, as their densities deteriorated significantly. Additionally, only a few residues remained associated with the used edge.

**Fig 7:**
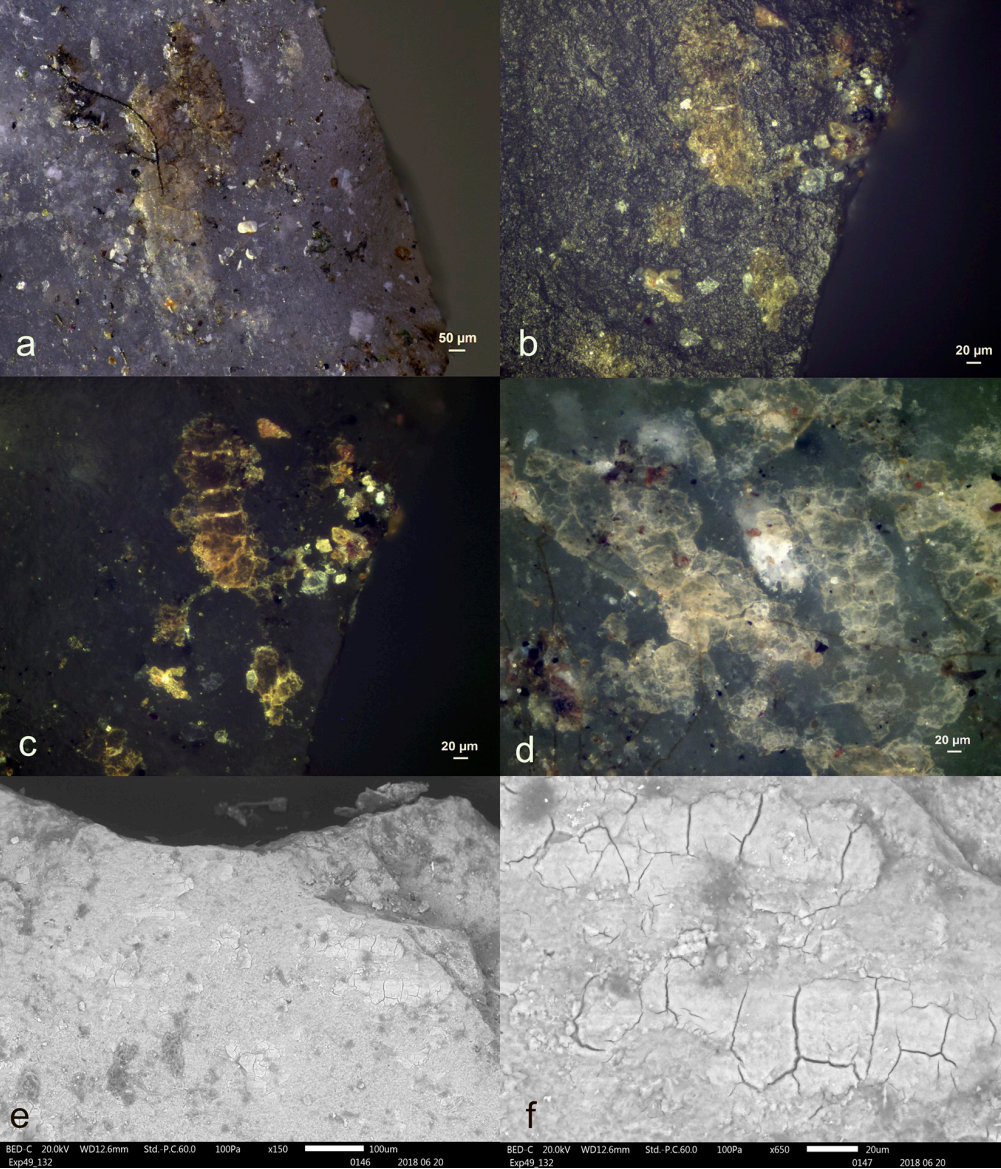
Visual characteristics of bone residues after short-term weathering exposure: Strong association with the used edge (a), (b), (e); significant degree of smearing (e), (f); brown discolouration (a), (b), (c); sharp edges and internal cracks (c), (d) (a+e+f = EXP49/132; 42 weeks), (b+c = EXP49/151; 8 weeks), (d = EXP49/170; 24 weeks)) (a = x11.2; b = x200; c = x200; d = x200; e = x150; f = x200).

Bone use residues were identified on fourteen meat processing tools, manifesting as brown-yellow patches of residue located on or near the utilized edge (as illustrated in **Fig 7**). These residues could be identified as hard animal material thanks to their characteristic flake-like morphology with incipient cracks and the distinctive presence of Calcium and Phosphorus peaks in the EDS spectra. Notably, most of the bone deposits exhibited a high density, significant smearing, and strong correlation with the used edge. Consequently, the potential for recognizing these residues as originating from use can be considered notably high.

**Fig 8:**
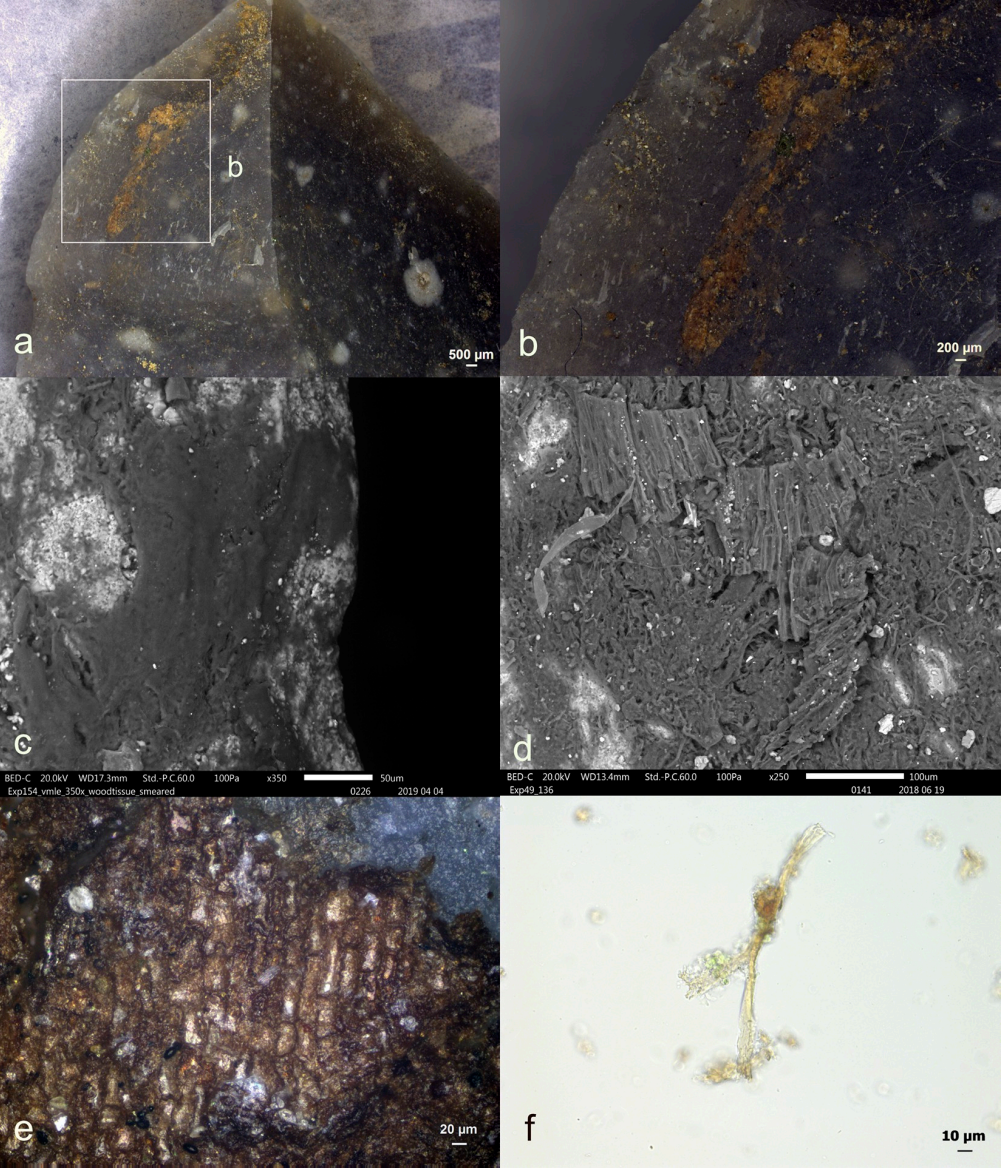
Visual characteristics of bone residues after short-term weathering exposure: Strong association with the used edge (a+b), strong degree of smearing (c), visible plant cells (d+e), cellulose fibres (f) (a+b = EXP49/135; 28 weeks) (c = EXP49/154; 36 weeks) (d = EXP49/136; 18 weeks) (e+f = EXP49/146; 24 weeks)) (a = x9.8; b = x25; c = x350; d = x250; e = x500; f = x400).

Wood use residues were evident on all twenty-two wood processing tools, forming continuous deposits parallel to the used edge (see **Fig 8**). These wood residues often appeared as dark brown, amorphous fragments when examined using stereo or incident light microscopy. The presence of plant cell structures and cellulose fibres within this amorphous mass provided a dependable means of identifying them as plant tissue. The unique plant components were most clearly discernible when observed under transmitted light, enabling the individual elements to be observed. The likelihood of identifying these residues as having a use-related origin is quite substantial, primarily due to their strong association with the edge used for processing, the notable degree of smearing, and the consistent parallel alignment toward the utilized edge—present on at least one of the faces of each tool, as indicated in (**Table 4**).

**Fig 9:**
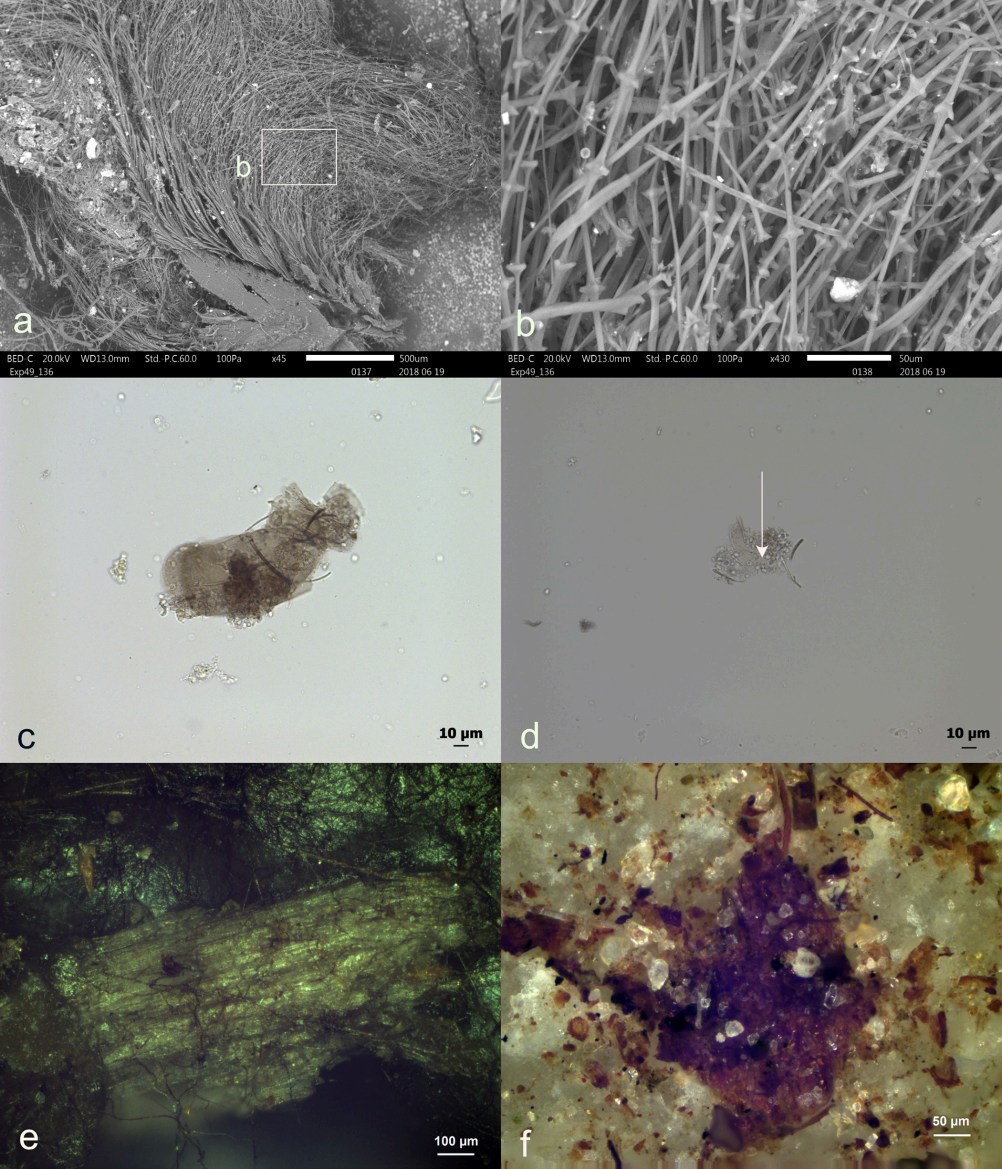
Visual characteristics of observed environmental residues: Accidental feathers (a+b); accidental insect wings (c+d), legs (d) and blood cells (d); environmental wood (e+f) (a+b = exp49/136; 18 weeks), (c+d = exp49/164; 42 weeks),(e = exp49/134; 10 weeks),(f = exp49/175; 6weeks)) (a = x45; b = x430; c = x400; d = x400; e = x100; f = x200).

Various environmental residues were observed on the tools, including wood tissue from the humic layer, pine needles, bird feathers (N = 2), insect wings (N = 1), and bird excrement (N = 1), as depicted in **Fig 9**. These residues were characterized by their absent or weak association with the edge used for processing and a lack or low degree of smearing. These features distinguished them as residual deposits of an environmental nature. The lack of smearing and the minimal adhesion of environmental wood made it easy to distinguish from wood residues related to hafting or use, which typically show significant smearing and strong adhesion. In the case of environmental plant residue, the plant tissue was not crushed, and the plant cells remained visible and completely intact. One might assume that a primary source of environmental wood deposition came from the overlying pine tree branches placed there to safeguard the stone tools from carnivores. Although these pine branches contain various organic compounds, including tannins, resins, and acids, their potential presence on the stone tools was not recognized through microscopy. However, these compounds may still be present in much smaller quantities.

#### Factors that influenced the effects of weathering

**Fig 10:**
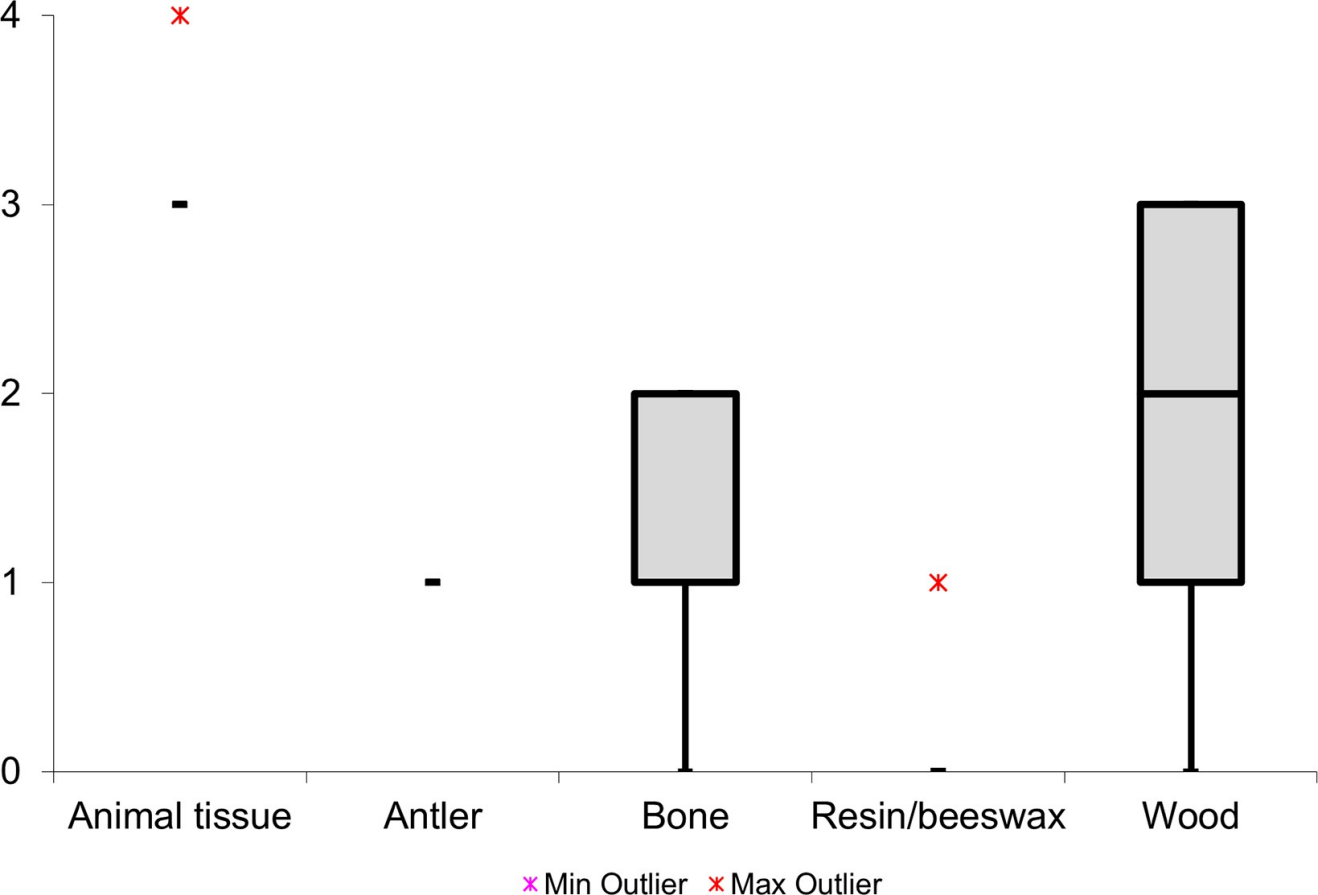
Observed residue loss for each residue type (loss scale: 0 = absent; 1 = weak; 2 = intermediate; 3 = strong; 4 = complete).

The **residue type** significantly influenced the extent of residue loss, primarily dictating the resistance of the residue to weathering processes. When considering soft animal residues, all deposits exhibited substantial or complete loss, as indicated in (**Fig 10**), which has most likely been caused by microbial activity. In sharp contrast, the resin/beeswax and antler deposits displayed minimal residue loss, showcasing their exceptional durability against weathering.

**Fig 11:**
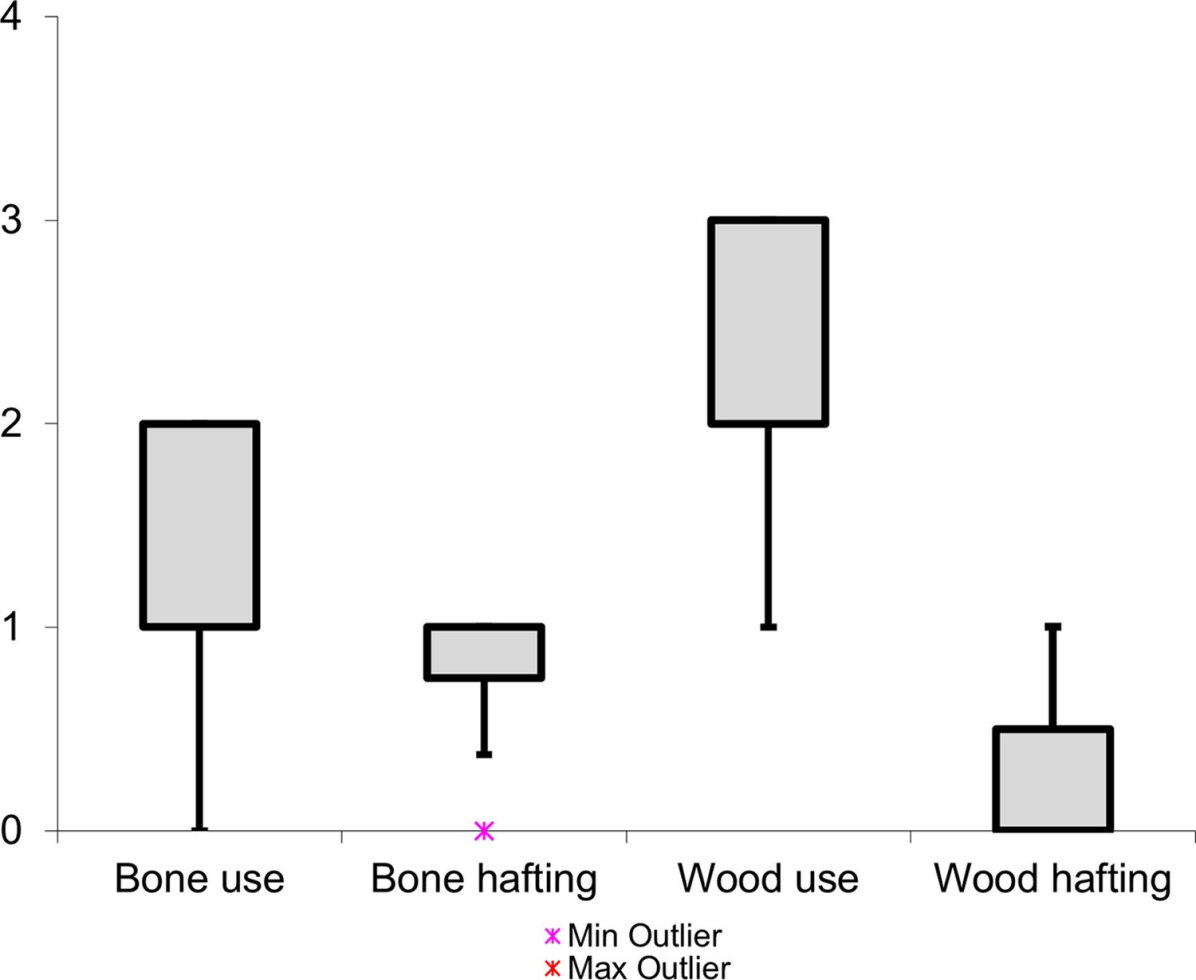
Residue loss for the different residue causes (loss scale: 0 = absent; 1 = weak; 2 = intermediate; 3 = strong; 4 = complete).

The **deposition process** appeared to have played an indirect role in the degree of residue loss. In the context of tool-use-related residues, thirty-five (80%) wood deposits and eight (36%) bone deposits were characterized by an intermediate or strong loss (**Fig 11**). This strongly contrasted with the hafting residues, where none of the deposits (wood, N = 7, or bone, N = 7) were characterized by such a degree of loss. Based on these results, it was hypothesized that thicker tool-use accumulations led to a stronger residue loss than the less dense hafting deposits.

**Fig 12:**
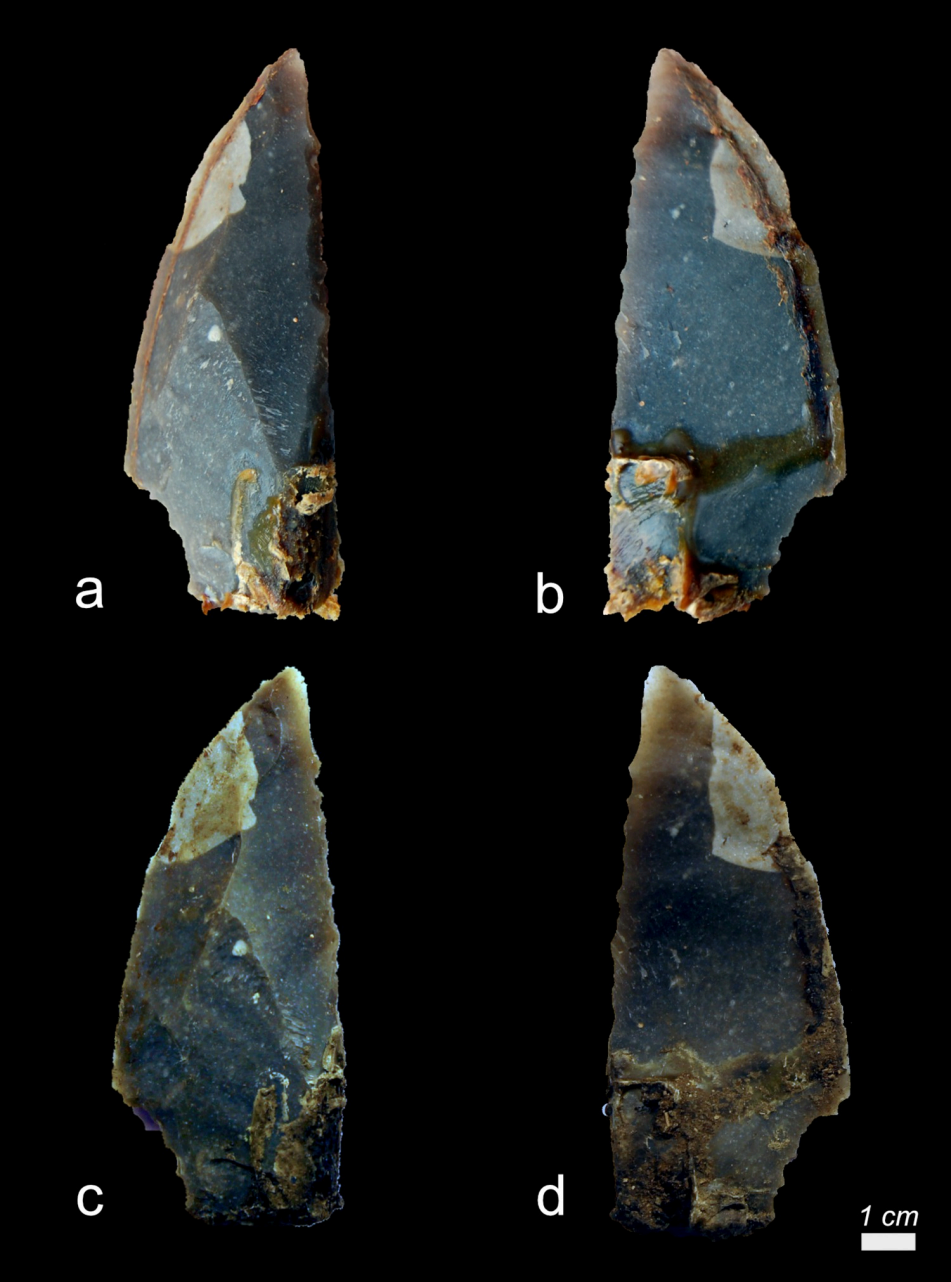
Wood cutting tool exp49/139 before and after being exposed to weathering for 26 weeks. The wood residue on the dorsal surface is preserved in higher densities on the ventral than on the dorsal surface. The resin beeswax remained completely intact.

**Fig 13:**
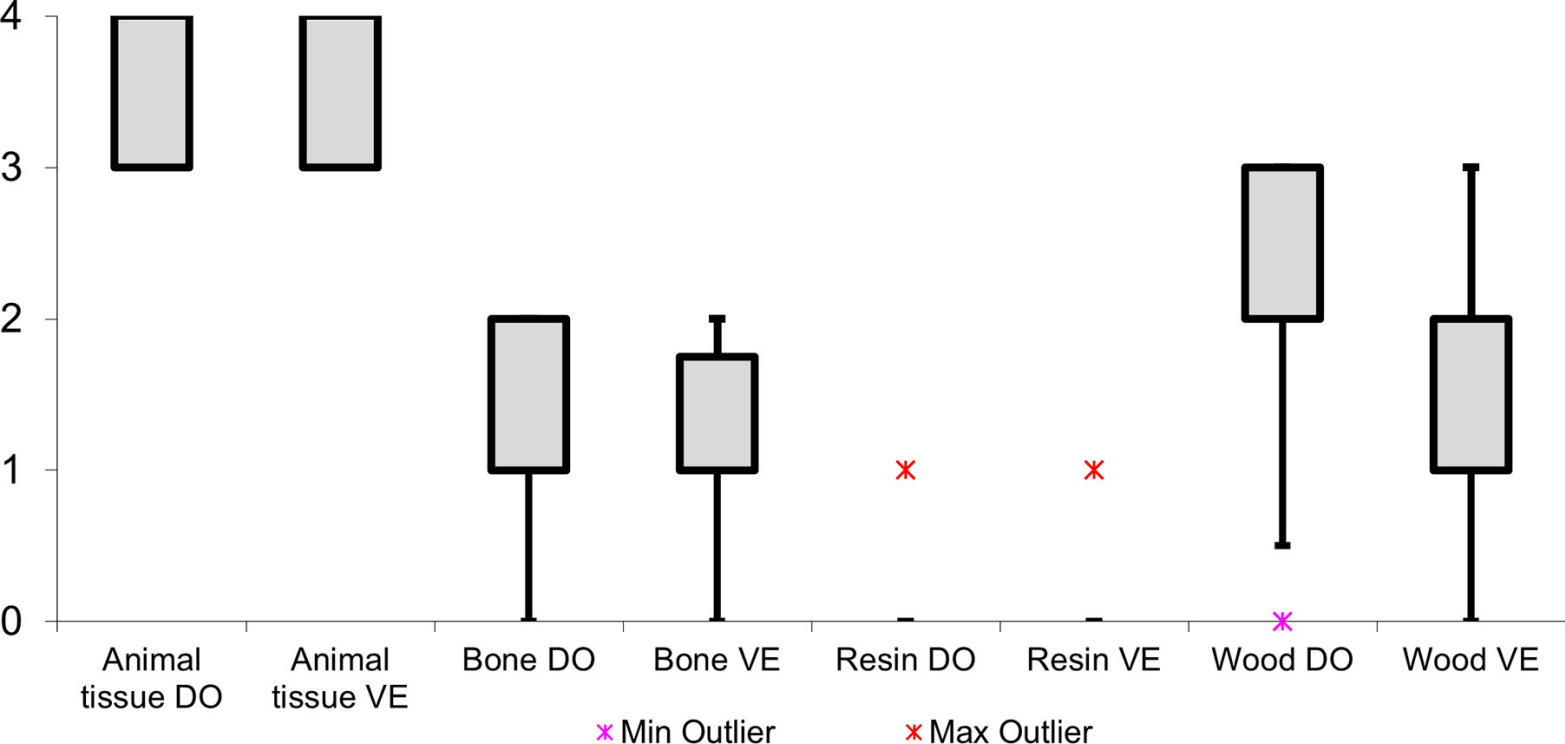
Residue loss for the different residue types and tool surfaces (DO = dorsal; VE = ventral) (loss scale: 0 = absent; 1 = weak; 2 = intermediate; 3 = strong; 4 = complete).

The orientation of the tool’s surface (whether it was positioned upwards or downwards) determined which weathering processes the residues were exposed to. In the context of wood processing residues, the loss of residues was more pronounced on the dorsal surface than on the ventral surface (as depicted in **Fig 12** and **Fig 13**). Ten (46%) out of the twenty-two dorsal deposits exhibited strong residue loss, contrasting four (18%) out of the twenty-two ventral deposits. These findings suggested that the loss of residues on the dorsal surface was primarily driven by the rapid dehydration of the wood tissue, which created cracks in the deposit and caused it to detach from the stone tool surface. The dehydration process occurred slower on the ventral surface, keeping the residual deposit attached to the stone tool surface.

Conversely, denser fungal growth on the ventral surface indicated that residue loss was mainly attributed to fungal decay in the case of bone and animal tissue residues. This denser fungal growth on the ventral surface could be attributed to its direct contact with the soil surface, contributing to the increased decay of residues. Drawing from these findings, we can hypothesise that residues on the upward-facing surface are more susceptible to mechanical weathering factors, such as rainfall, while those on the downward-facing surface are directly influenced by biological weathering, particularly soil microbial activity. The ability of a particular residue type to withstand these specific weathering processes will consequently dictate its likelihood of enduring over time.

**Fig 14:**
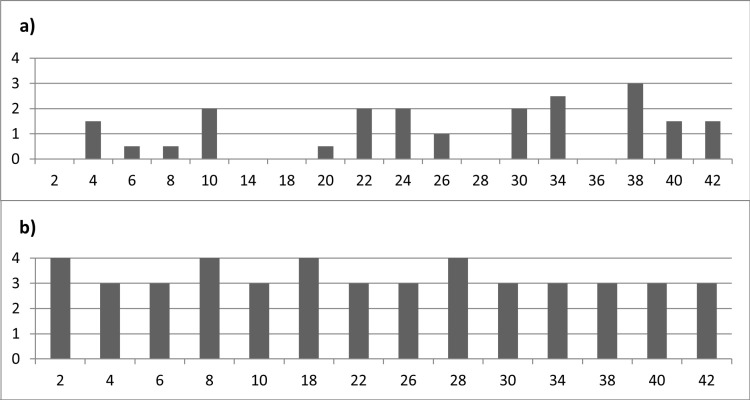
Observed densities (a) and loss (b) versus exposure time in weeks for soft animal residues.

The **duration of exposure** did not have a significant impact on the effect of weathering on residues. When examining soft animal residues (as illustrated in **Fig 14**), it was evident that the tool exposed for just two weeks (exp49/165) had already experienced a complete loss of residue, underscoring the swift decay of these particular residues. In this context, enzymatic degradation likely played a pivotal role in causing such rapid and substantial loss. Tools exposed for periods from 4 to 42 weeks exhibited a consistent pattern of strong or complete residue loss. This observation reinforces the notion that most soft animal residues tend to decay within a mere two weeks (as demonstrated in **Fig 17A+17B**), with only sporadic instances of survival. The irregular persistence of these residues is likely tied to hyperlocal factors, such as variations in microbial activity, further emphasizing the intricate nature of biological weathering.

**Fig 15:**
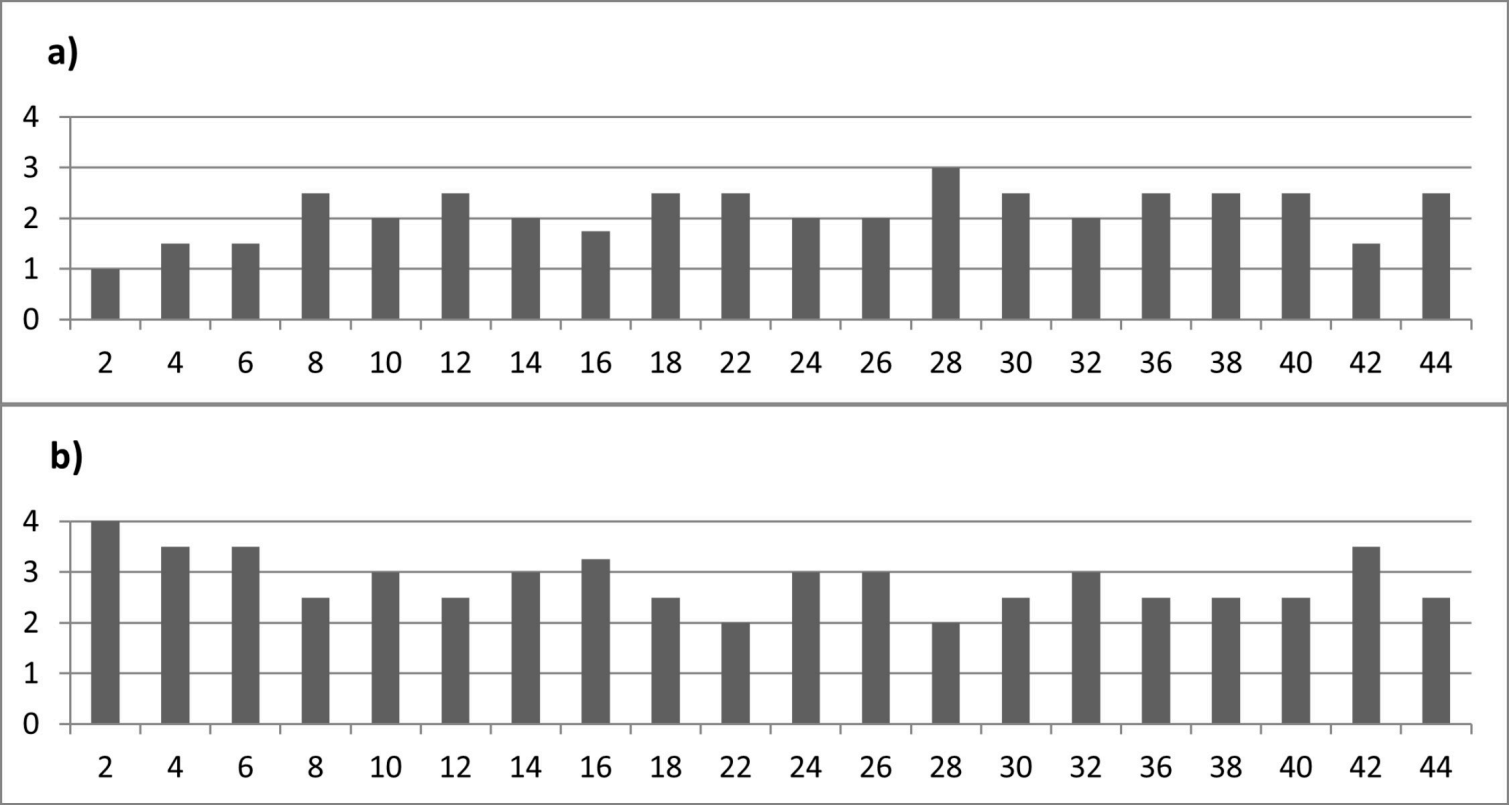
Observed densities (a) and loss (b) versus exposure time in weeks for wood use residues.

Concerning wood use residues (depicted in **Fig 15**), the loss of residues became notably apparent after just two weeks of exposure (**Fig 17C+17D**), and this loss remained relatively constant for tools exposed over more extended periods. It can be posited that the wood residues with weaker adhesion were mechanically removed from the stone tool surface during the initial weeks of exposure. Conversely, the duration of exposure did not significantly impact the wood residues that were more firmly attached.

**Fig 16:**
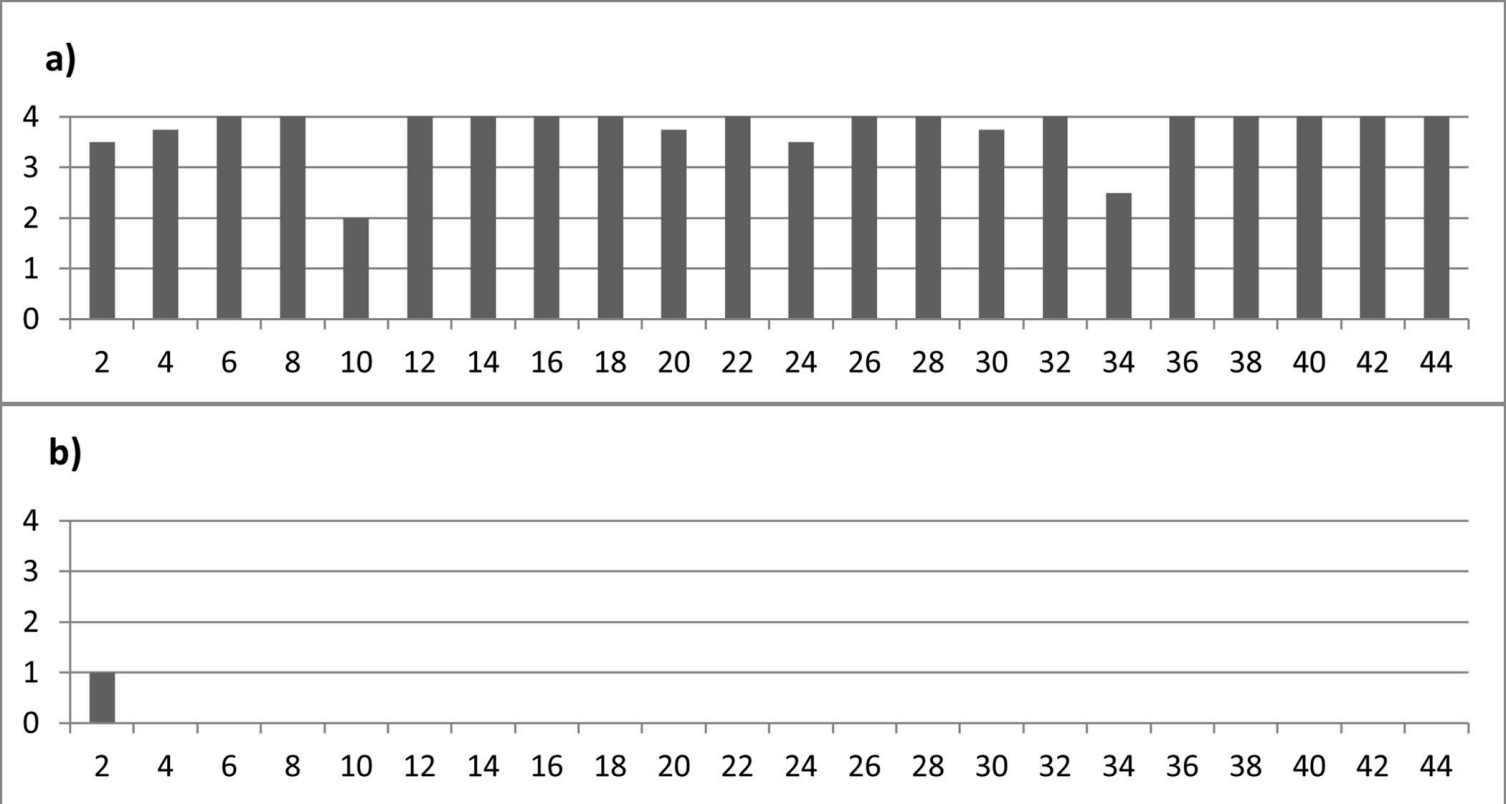
Observed densities (a) and loss (b) versus exposure time in weeks for resin and beeswax residues.

**Fig 17:**
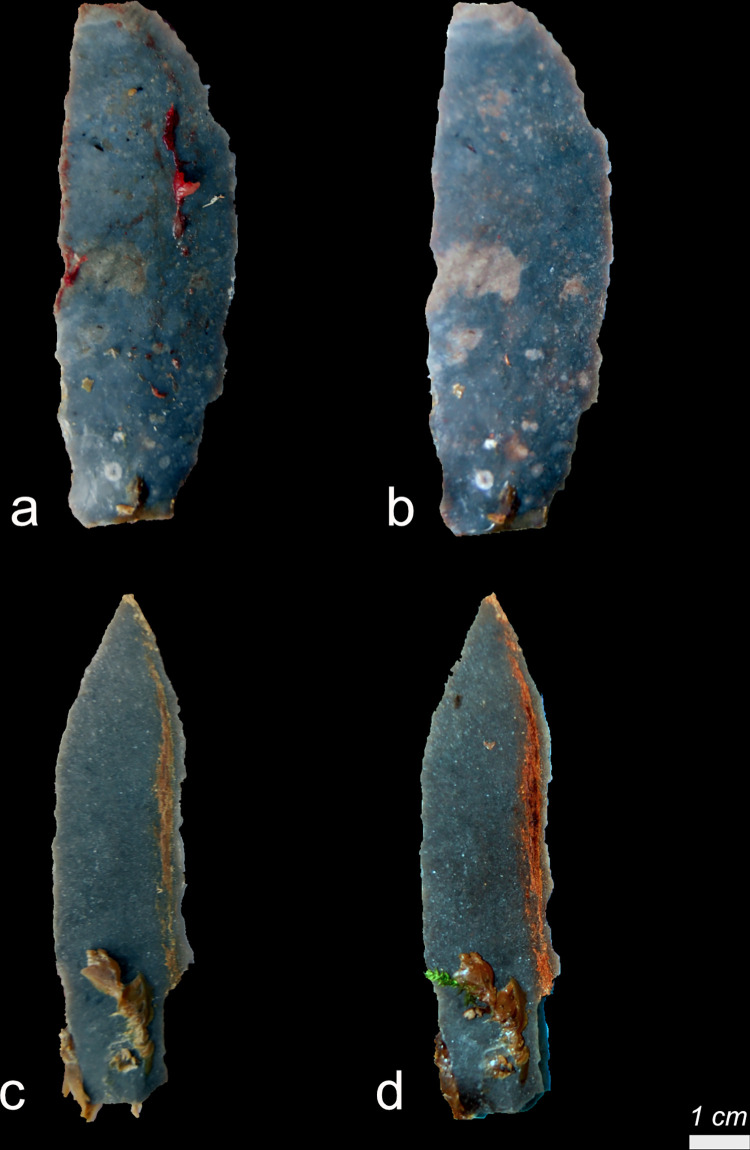
Impact of two-week weathering exposure on stone tool residues. The impact on the butchering residues of exp49/165 (a+b) is significantly higher than on the wood (c+d) or resin and beeswax residues(a+b+c+d) of exp49/141.

As for resin and beeswax deposits (referenced in **Fig 16**), they remained largely intact for a minimum of two weeks **(Fig 17A+17B+17C+17D)**, except for the resin and beeswax present on the exposed tools.

### Three-year surface experiment

#### Impact on the recognition of stone tool residues and their deposition processes

When examined with the stereomicroscope, possible production residues were observed on two (Exp49/53 and Exp49/54) of the sixteen stone tools. These residues were identifiable by their pronounced degree of smearing and localized distribution on the butt, as indicated in **Table 5**. The white colour of both residual deposits suggested they could be antler production residues. However, these residues could be mistaken for similar-looking residues, such as other calcium-rich residues, necessitating a more comprehensive analysis.

**Fig 18:**
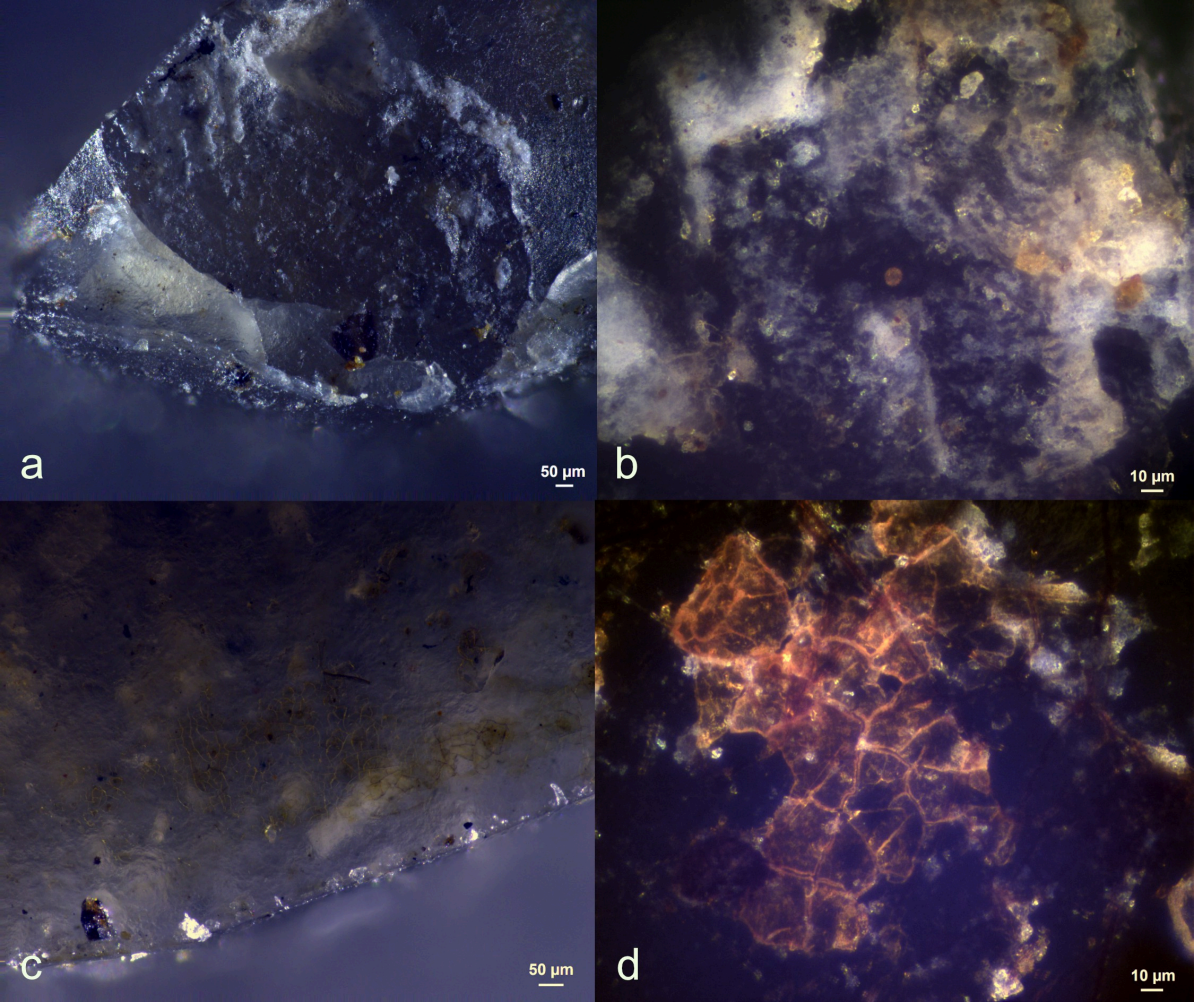
Visual characteristics of authigenic calcium deposit on the butt of tool exp49/53 (Rochefort; 3 years): Isolated location on the butt (a+b), white colour (b+c+d), the granular internal structure (d)) (a = x47; b = x180; c = x200; d = x500).

The residue on tool Exp49/53 appeared white and granular when viewed under brightfield illumination, while the residue on tool Exp49/54 appeared reflective and flat. Under cross-polarized light, the visual distinction became even more apparent: the residue on tool Exp49/53 remained a white, granular deposit (as seen in **Fig 18**), whereas the residue on tool Exp49/54 appeared as a brown-yellow deposit with cracks and sharp edges. Further differentiation was achieved through EDS spectroscopy using the scanning electron microscope, which classified the residues as calcium deposits (Exp49/53) due to their pure Calcium (Ca) composition and antler production residue (Exp49/54) due to their composition of Calcium (Ca) and Phosphorus (Ph).

**Fig 19:**
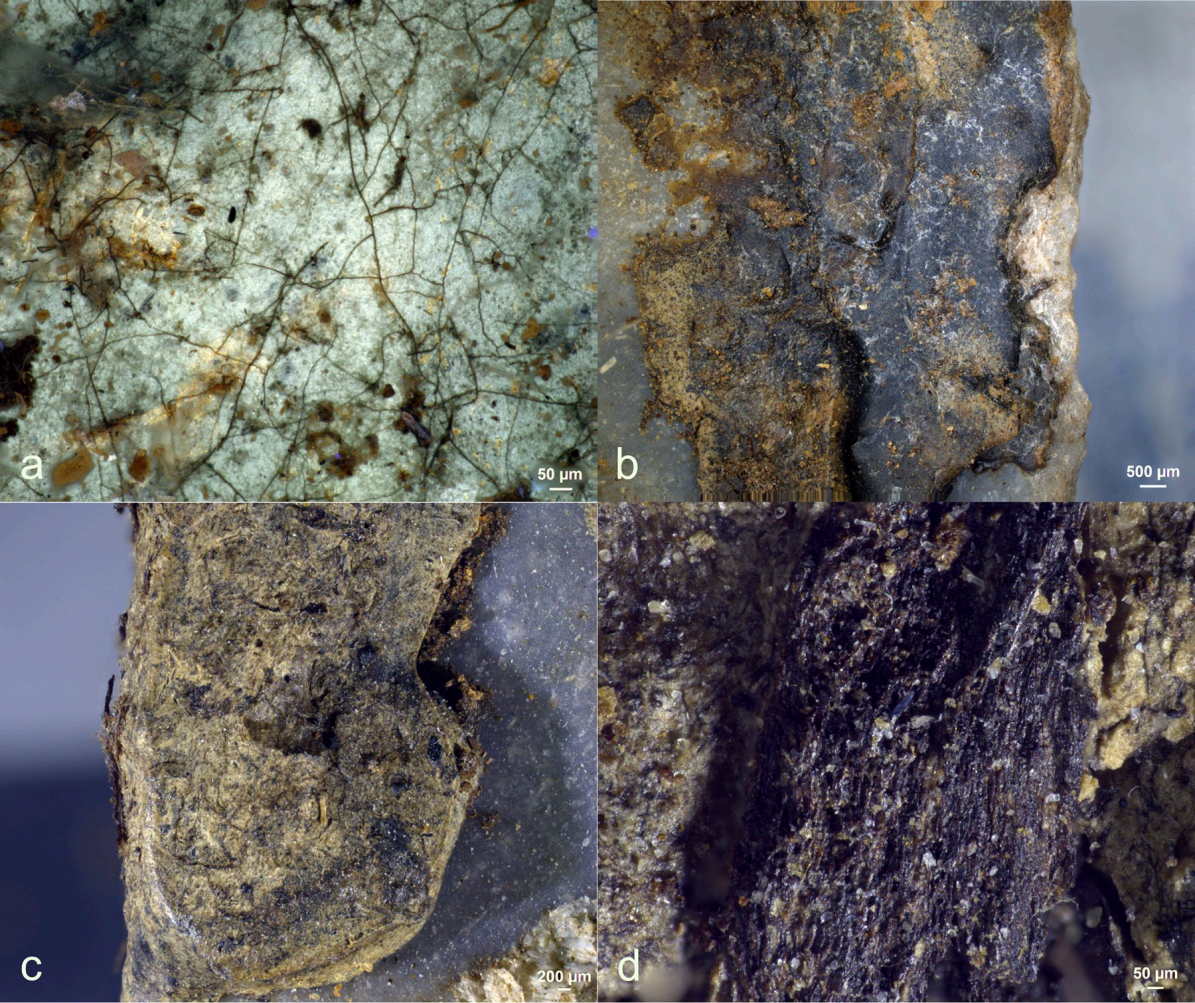
Visual characteristics of hafting residues after three-year weathering on tool exp49/44 (Rochefort; 3years): Intense fungal growth (a), dark brown discolouration of resin and beeswax (a+b), authigenic calcium deposit on resin beeswax (b), dark brown discolouration of wood hafting residues (d)) (a = x88; b = x19; c = x22; d = x120).

Even though eight stone tools (EXP49/08, EXP49/16, EXP49/20, EXP49/28, EXP49/32, EXP49/40, EXP49/44, EXP49/54) were originally hafted with leather bindings, no traces of binding residues were detected. Only one stone tool (Exp49/44) had been hafted with resin and beeswax, and remarkably, this adhesive residue remained entirely intact, even in the presence of vigorous fungal growth. The resin and beeswax mixture displayed noticeable discolouration, taking on a dark appearance that bore visual similarities to wood tar (see **Fig 19**).

While four stone tools were originally hafted with bone shafts (EXP49/08, EXP49/16, EXP49/20, EXP49/54), no discernible bone shaft residues were identified. The possibility of wood shaft residues was only evident on one stone tool (EXP49/44), where the wood residue was found embedded within the resin and beeswax (**Table 6**).

**Fig 20:**
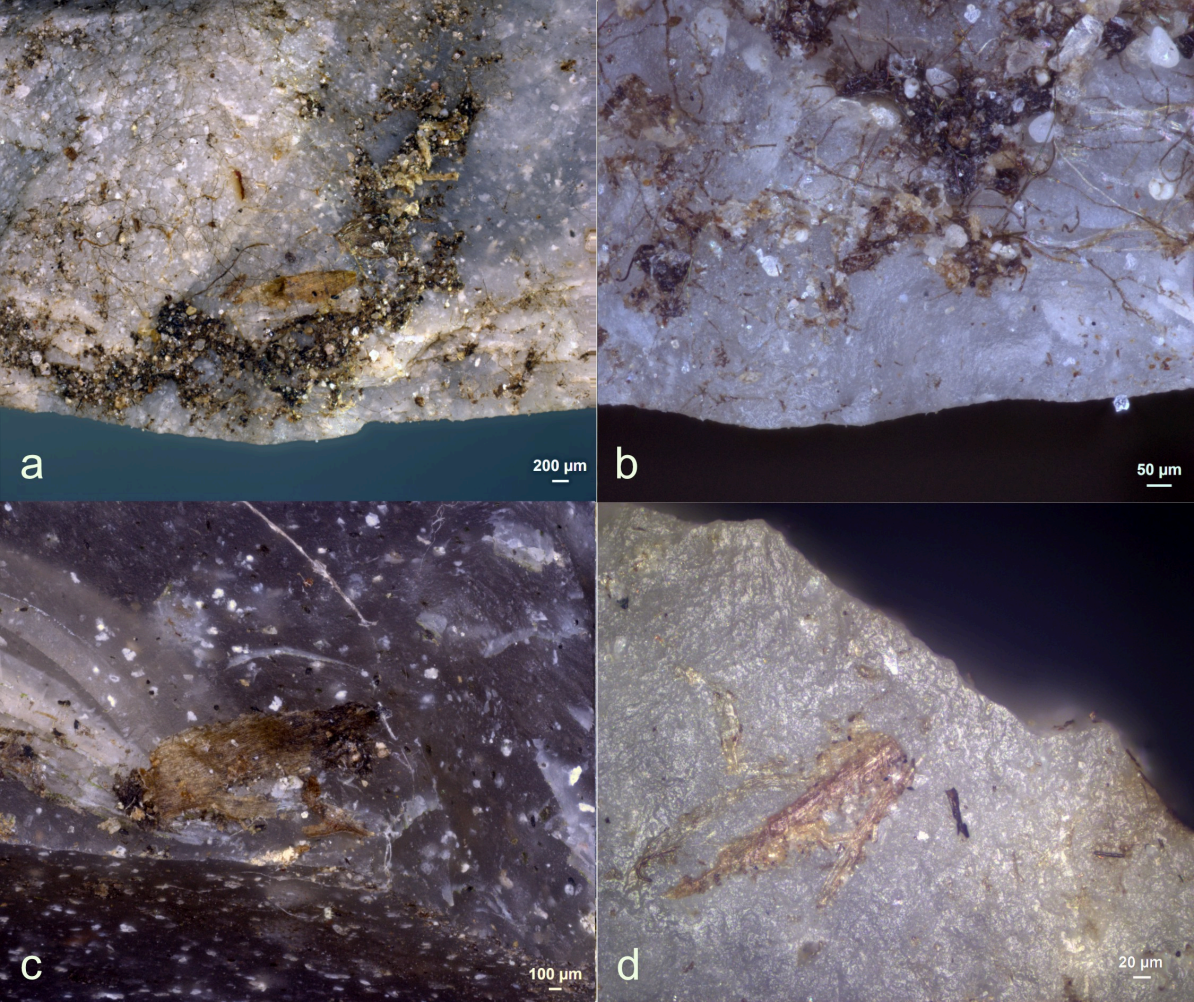
Visual characteristics of wood use residues after three-year weathering: Strong association with the used edge (a+b+c+d), an intermediate degree of smearing (c+d), perpendicular directionality against the used edge (c+d), dark brown discolouration (a+b) (black arrows indicate wood use residues; red arrow indicates environmental wood) (a+b+d = exp49/08; Lommel) (c = exp49/07; Lommel)) (a = x30; b = x65; c = x46; d = x200).

Potential wood residues related to use were observed on two (EXP49/07 and EXP49/08) of the four tools employed for woodworking (as shown in **Fig 20**). Differentiating these use-related wood residues from the omnipresent environmental residues on the soil surface presented a considerable challenge. A pivotal criterion for differentiation between the two residue types rested on the conspicuous alignment of use residues with the utilized edge (distal end), observed on both the dorsal surfaces (as evident in **Fig 20A+20B**) and ventral surfaces. Nonetheless, it’s important to highlight that certain environmental wood residues were also near the utilized edge, underscoring that location or distribution alone could not be the sole determinant for identifying wood use residues. It underscored the necessity of effectively employing a combination of criteria to distinguish between use-related and environmental residues. Therefore, recognizing wood use residues requires a strong association with the employed edge, a notable degree of smearing, and a perpendicular orientation towards the utilized edge. The latter two features emerged due to the pressure applied during use. In the case of the end-scraper, this pressure was localized to the point of contact between the worked material and the ventral surface.

Moreover, the overwhelming majority of wood residues had significantly deteriorated due to the impact of weathering processes. This degradation resulted in their conversion into a dark, amorphous mass, void of visible cell walls or fibres, thereby challenging their identification. These distinctive characteristics were only observable in a minority of the use-related wood residues on the ventral face and the more recently deposited environmental residues.

**Fig 21:**
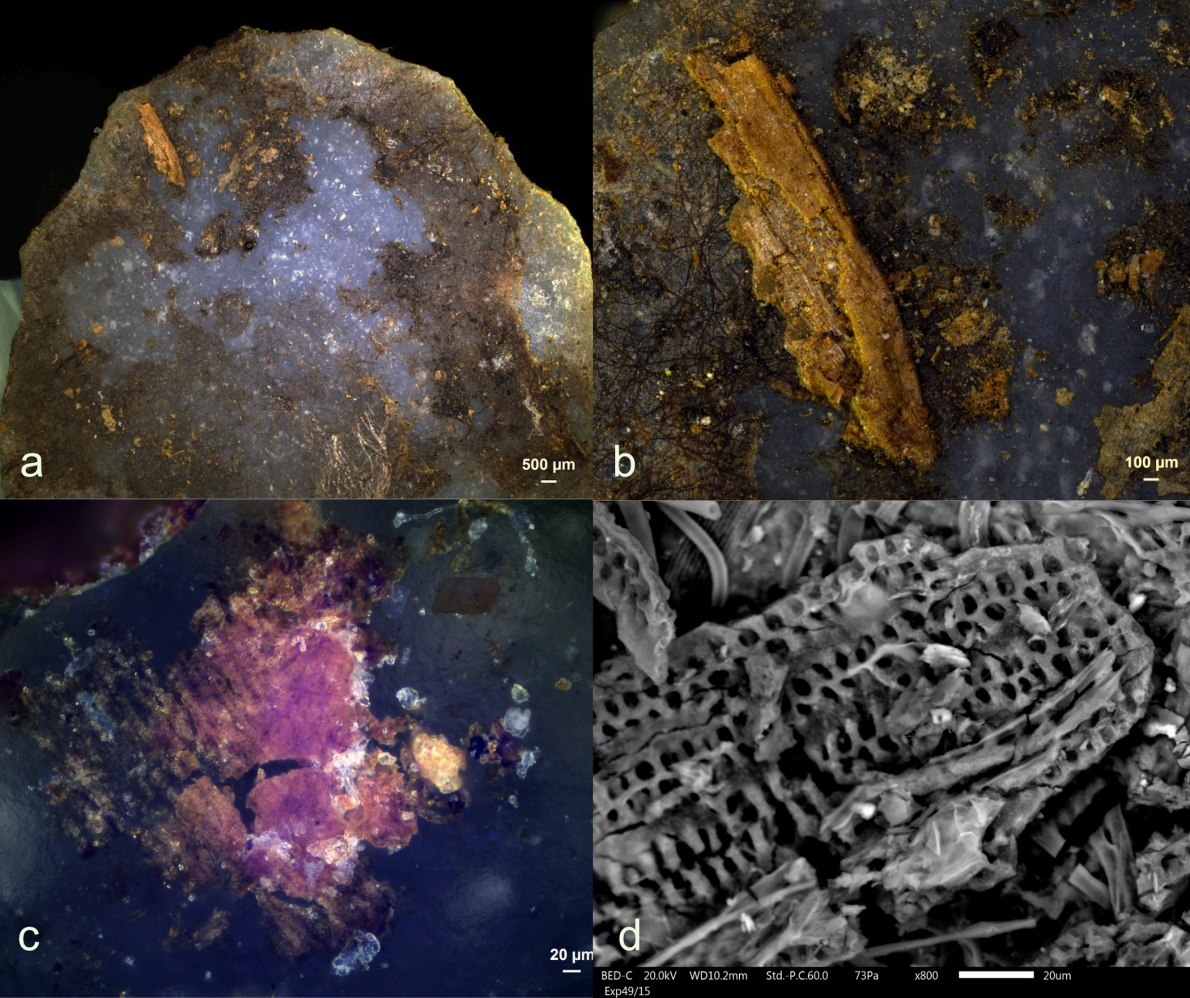
Visual characteristics of plant use residues after three-year weathering: Dispersed distribution (a), weak association with the used edge (a+c), intermediate degree of smearing (c), dark brown discolouration (a+b), intact plant cells (d)) (a = x30; b = x65; c = x200; d = x800).

Potential use-related soft plant residues were detected on two of the four plant working tools (EXP49/15 and EXP49/16), as depicted in (**Fig 21**). In these cases, there was a relatively substantial loss of residues, yet the remnants persisted in large, visible patches (with a density of around 2.2). The soft nature of plant material led to a relatively weak to intermediate connection with the utilized edge, a limited degree of smearing, and a lack of clear directionality. The absence or limited development of these attributes somewhat complicated the certainty of interpretation. However, both plant cell walls and cellulose fibres were abundant and visible, providing a secure basis for identifying these residues as plant material.

**Fig 22:**
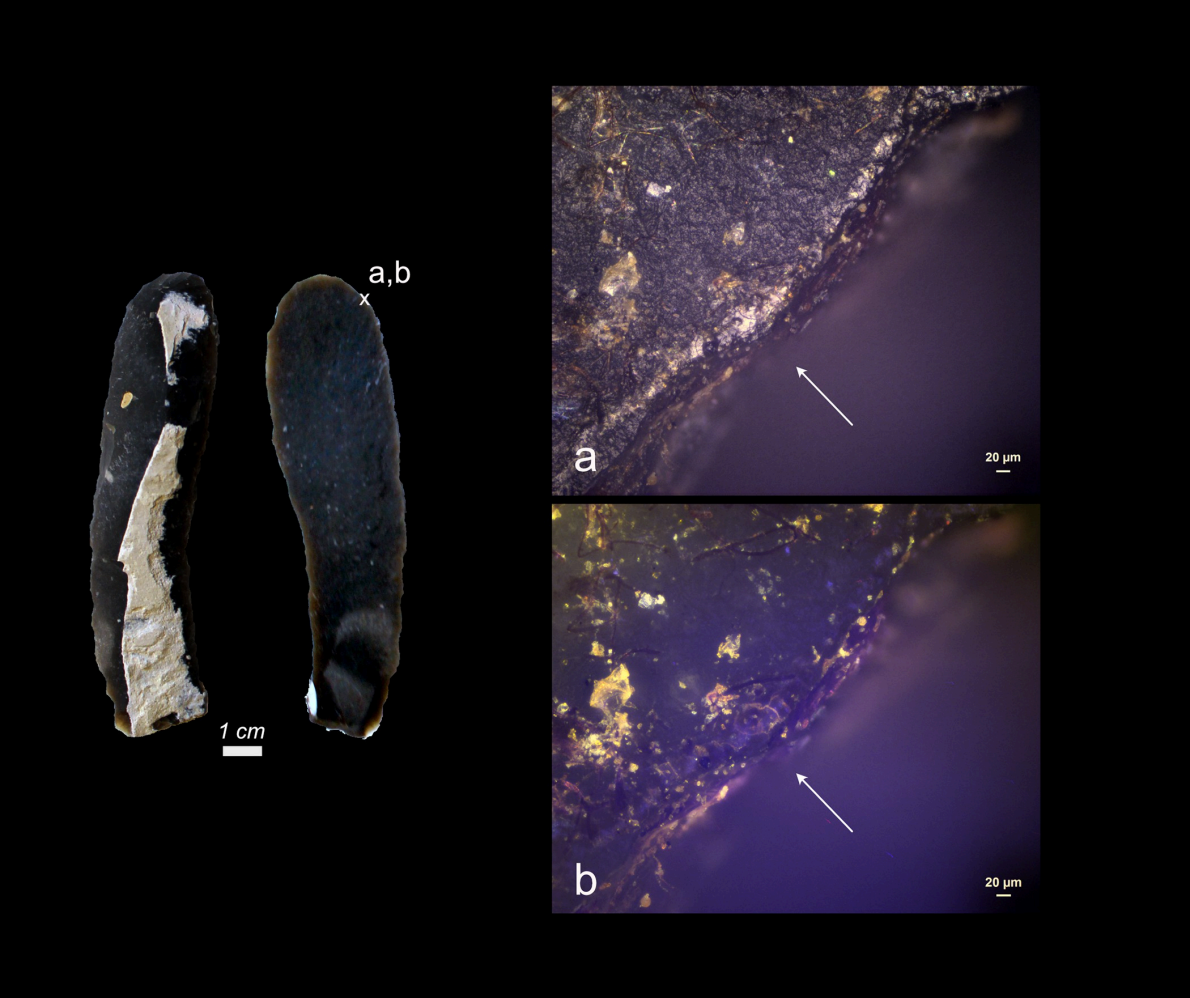
Visual characteristics of bone use residues on tool exp49/31 (Rochefort) after three-year weathering: Isolated distribution (a+b), strong association with the used edge, highly reflective appearance under bright field reflected light (a), brown, cracked and flat under cross-polarized light (b)) (a = x200; b = x200).

Use-related bone residues were identified on just one out of the four stone tools, as indicated in **Fig 22**, suggesting that most of the bone residues had dissolved over time. The sole stone tool exhibiting adhering bone residue (Exp.49/31) displayed a relatively low density (d1). Nevertheless, a distinct association with the utilized edge and a pronounced degree of smearing positively identified the residue as being use-related. Under brightfield illumination, the bone residue appeared reflective and translucent, while it had a brown, cracked and flat appearance under cross-polarised light.

**Fig 23:**
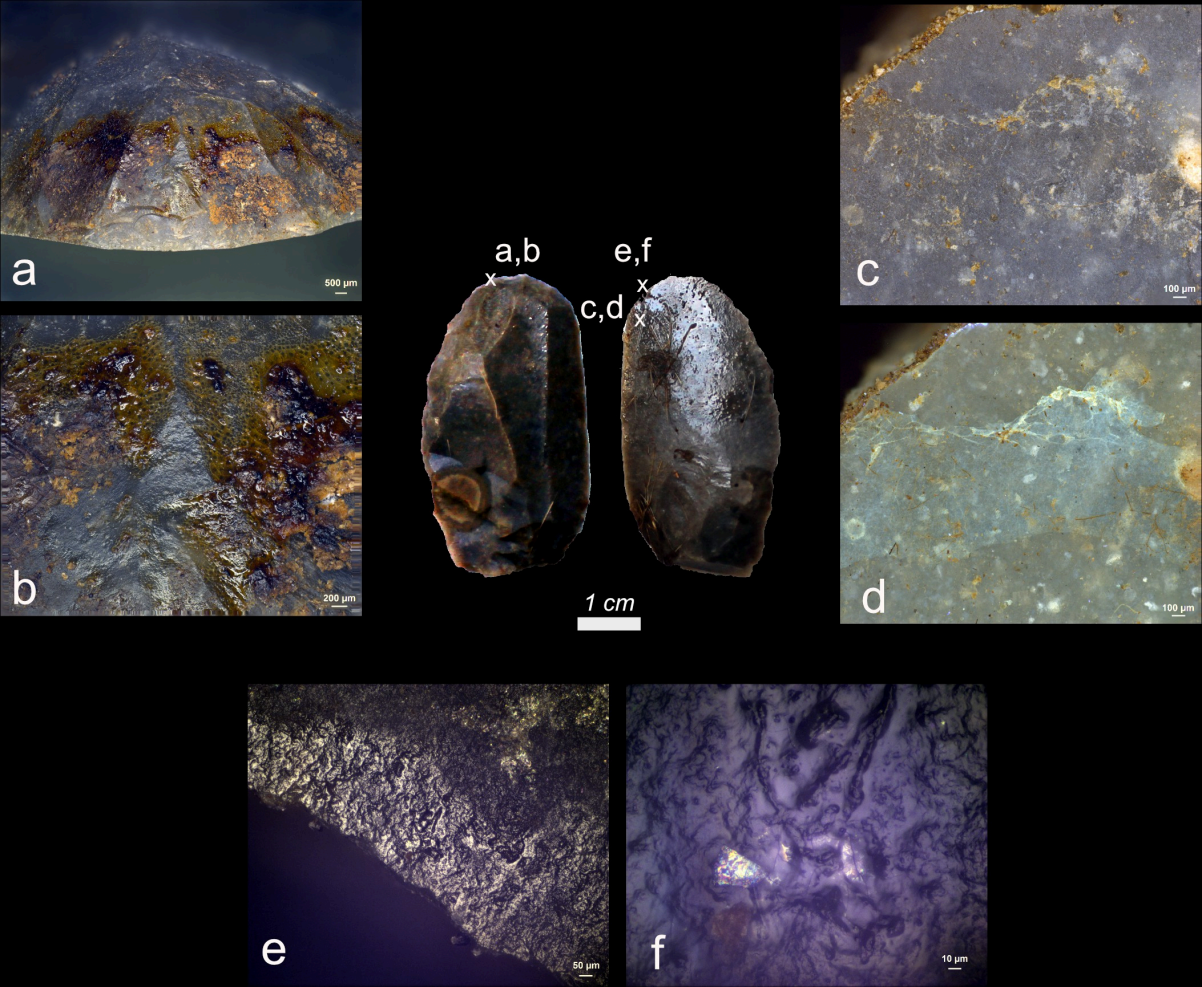
Visual characteristics of possible hide use residues on tool exp49/44 (Rochefort) after three-year weathering: Dispersed distribution (a+c), intermediate association with the used edge (a+c+d), brown discolouration on the dorsal surface (a+b), and translucent appearance on the ventral surface (c-f), best visible with DAPI fluorescence filter (d)) (a = x13.8; b = x50; c = x80; d = x80; e = x100; f = x500).

Possible use-related hide residues, (**Fig 23**), were observed on all four tools, with three specifically being hide scraping tools (EXP49/39, EXP49/40, EXP49/43, EXP49/44). These tools displayed an intermediate to low level of residue loss, resulting in intermediate to high residue densities. The potential hide residues were primarily preserved as a thin layer, although the auto-fluorescence filter facilitated a more comprehensive observation of the complete residual deposit. The intermediate degree of smearing and the dispersed distribution of residues could potentially complicate the positive identification of their use-related origin. Consequently, the strong association of these residues with the utilized edge (distal end) emerged as a crucial factor in confirming their use-related nature.

Furthermore, the hide residues underwent distinct transformations on the dorsal and ventral surfaces, influenced by their exposure to air. The hide residues on the dorsal surface, facing upwards, underwent drying and deoxygenation, resulting in a cracked, brown to red residual deposit (as depicted in **Fig 25A+25B**). In contrast, the hide residues on the ventral surface remained translucent, as they were shielded from direct exposure to air (as seen in **Fig 25C–25F**).

**Fig 24:**
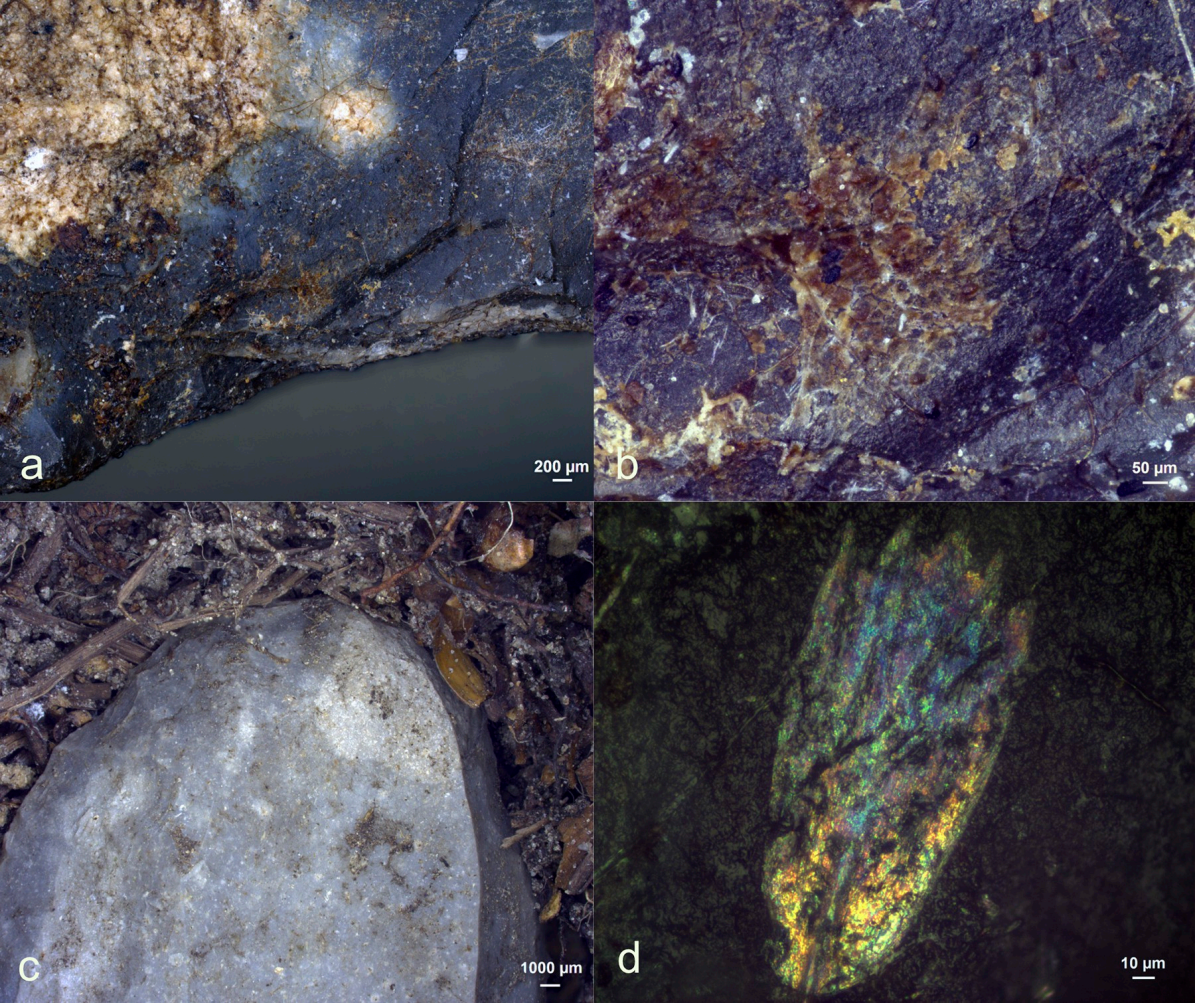
Visual characteristics of environmental residues after three-year weathering a) environmental wood tissue strongly associated with the used edge of bone working tool (Exp49/31) (b) detail of environmental wood residue on tool Exp49/31 c) Exp49/08 surrounded with wood fragments from humus d) Insect wing on woodworking tool Exp49/27 (a+b = EXP49/31; Rochefort); (c = exp49/08; Lommel); (d = Exp49/27; Lommel)) (a = x34; b = x180; c = x7.2; d = x200).

Various environmental residues were detected on the tools (**Fig 24**). Specifically, wood tissue was observed along the distal edge of two bone-working tools (EXP49/27 and EXP49/31). Notably, the wood tissue on one of these tools (EXP49/31) exhibited a strong degree of smearing and a perpendicular alignment against the utilized edge, which could potentially lead to misinterpretation as use-related residue. In addition, insect wings (EXP49/07) were found near the edge used by one woodworking tool.

### Burial experiment

#### Impact on the recognition of stone tool residues and their deposition processes

**Fig 25:**
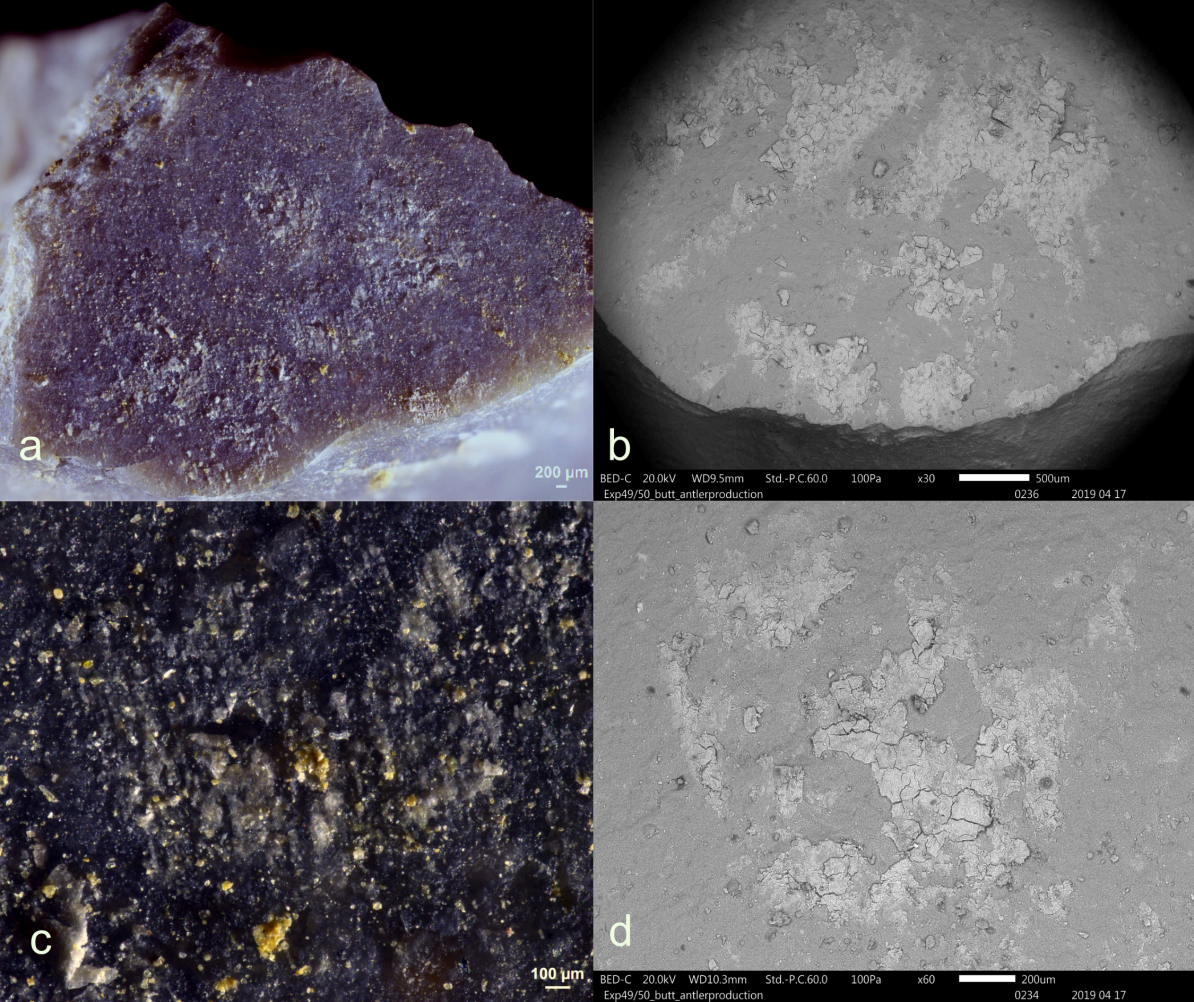
Visual characteristics of antler production residues on tool exp49/50 (Scladina) after three-year burial: Local distribution on the butt (a+b), a significant degree of smearing (C), flat topography a-d), sharp edges and internal cracks) (a = x19.4; b = x30; c = x90; d = x60).

Antler production residues (as detailed in **Table 8**) were only evident on one tool (EXP49/50) (**Fig 25**), among the twenty-eight stone tools that were manufactured and modified using an antler hammer. This residual deposit stood out and could be readily observed with a stereoscope due to its substantial size, high density, and initial white colour. A considerable degree of smearing was noticeable, significantly facilitating the identification of the residue’s origin.

Upon closer examination under cross-polarized light and with a scanning electron microscope, a plate-like structure within the antler deposit became discernible. An EDS analysis further substantiated the significant presence of Calcium and Phosphorus. Given the high density of the residual deposit, it strongly suggests that the residue loss would have been minimal, allowing the original residual deposit to remain predominantly intact.

No evidence of wood production residues was observed on the seven stone tools produced with a wooden hammer.

**Fig 26:**
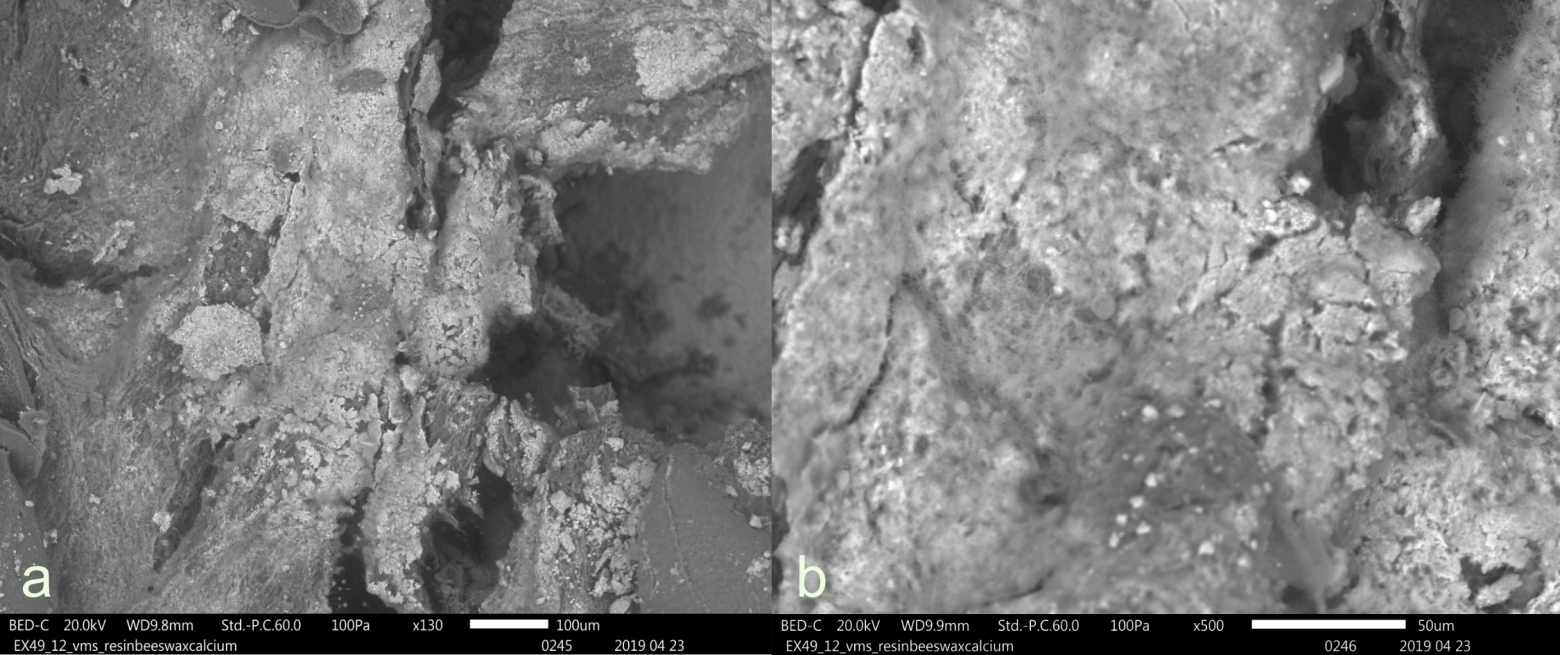
Visual characteristics of calcium residues adhering to resin beeswax that can be mistaken for bone handle residues on exp49/12 (three-year burial; scladina) a) authigenic calcium deposit on top of resin-beeswax mixture b) detail of calcium deposition, showing the microcrystalline structure) (a = x130; b = x500).

Even though nine tools had been originally hafted with a bone handle, no traces of bone handle residues could be identified. While the use of stereo- and incident light microscopes allowed for the observation of white residues on the medial surfaces of several tools, such as Exp49/12 (as illustrated in **Fig 26**), an EDS analysis confirmed that these residues were, in fact, authigenic calcium deposits.

**Fig 27:**
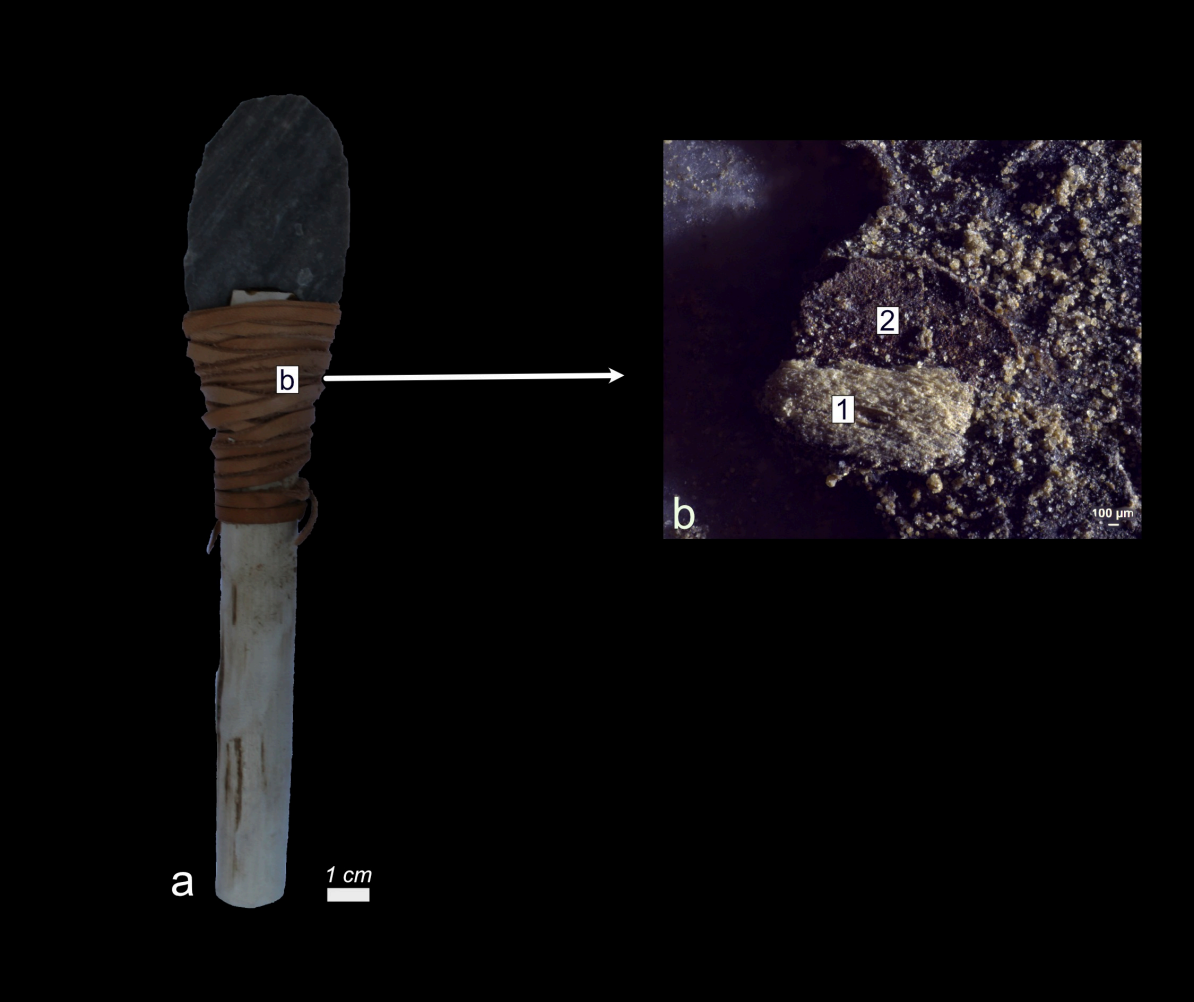
Visual characteristics of rootlet residues that can be mistaken for wood handle residues on a) Exp49/48 (three-year; Val-Meer) b) plant tissue identified as rootlet due to the presence of intact dark brown epidermal tissue (2) with hollow morphology (2) and light brown xylem tissue with large rectangular cells (1) and an absent degree of smearing (b = x52).

Although eight tools had been hafted with a wooden handle, no traces of wood handle residues could be identified. However, on one tool (Exp49/48), as depicted in **Fig 27**, a substantial piece of wood tissue (visible to the naked eye) adhered to the adhesive. The presence of epidermal tissue indicated that this residue was most likely a rootlet.

**Fig 28:**
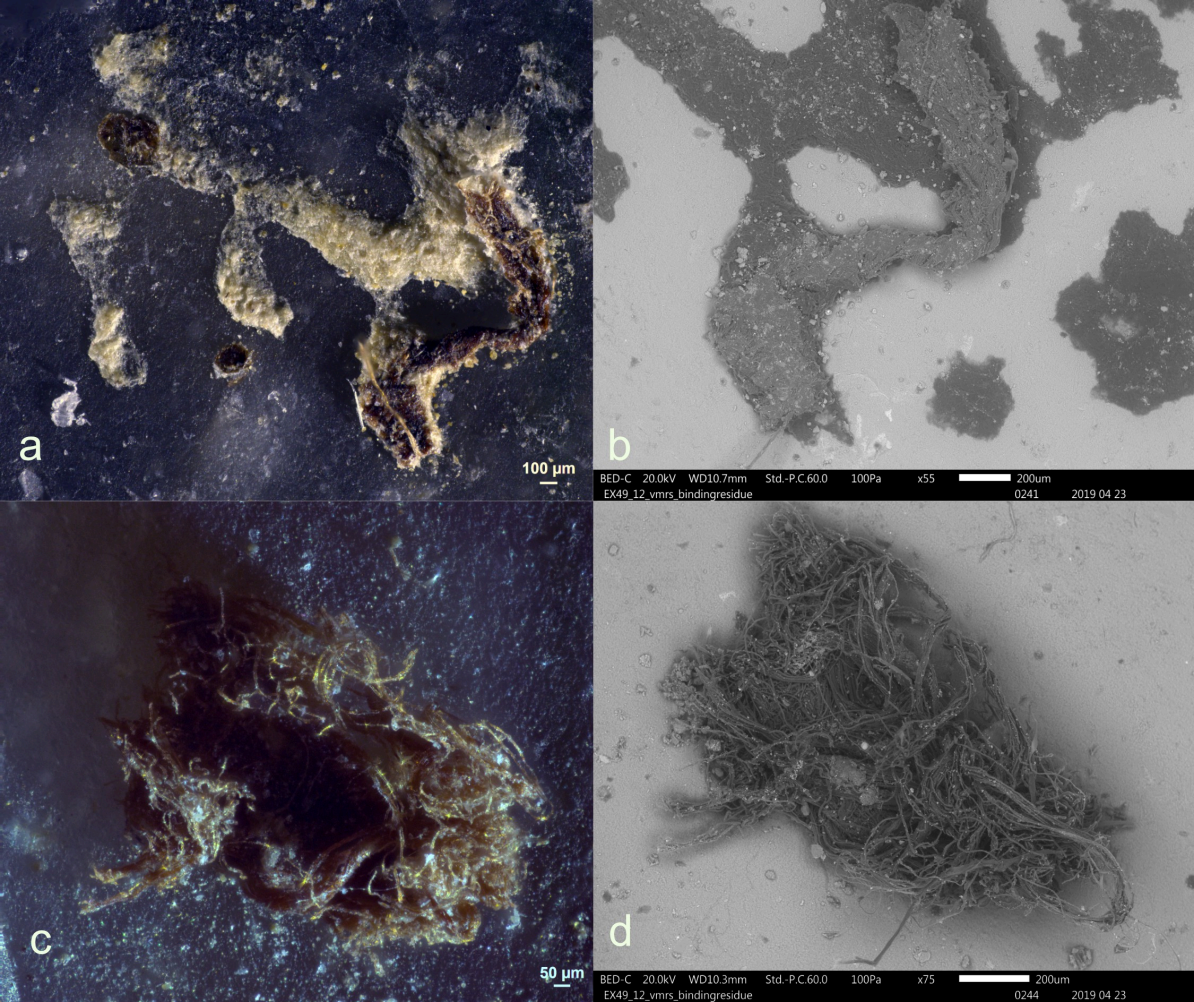
Visual characteristics of sole leather binding residues (Exp49/12; Scladina) that were identified after three-year burial: Twisted nature remained (a+b), fine fibrous structure (c+d) (a = x63; b = x55; c = x120; d = x175).

Residues from leather bindings were identified on one tool, specifically Exp49/12, as shown in **Fig 28**. These residues appeared as clusters of twisted fibres. One of the fibre deposits was affixed to the hafting adhesive, while the other was directly deposited onto the stone tool surface.

Adhesive residues were evident on all ten tools that had been hafted with resin and beeswax (EXP49/12, EXP49/14, EXP49/18, EXP49/26, EXP49/30, EXP49/38, EXP49/42, EXP49/48, EXP49/52, and EXP49/56), and their identification was made possible due to the enduring high densities. Over time, all the resin and beeswax deposits shifted colour, transitioning from a light brown hue to dark brown or black. The resin and beeswax deposit (Exp49/38) retained a partial yellowish tint in one exceptional case. Despite the adhesive deposits displaying an average high fungal density (with a density rating of 4), this did not affect their preservation, as no residue loss or fragmentation was observed.

**Fig 29:**
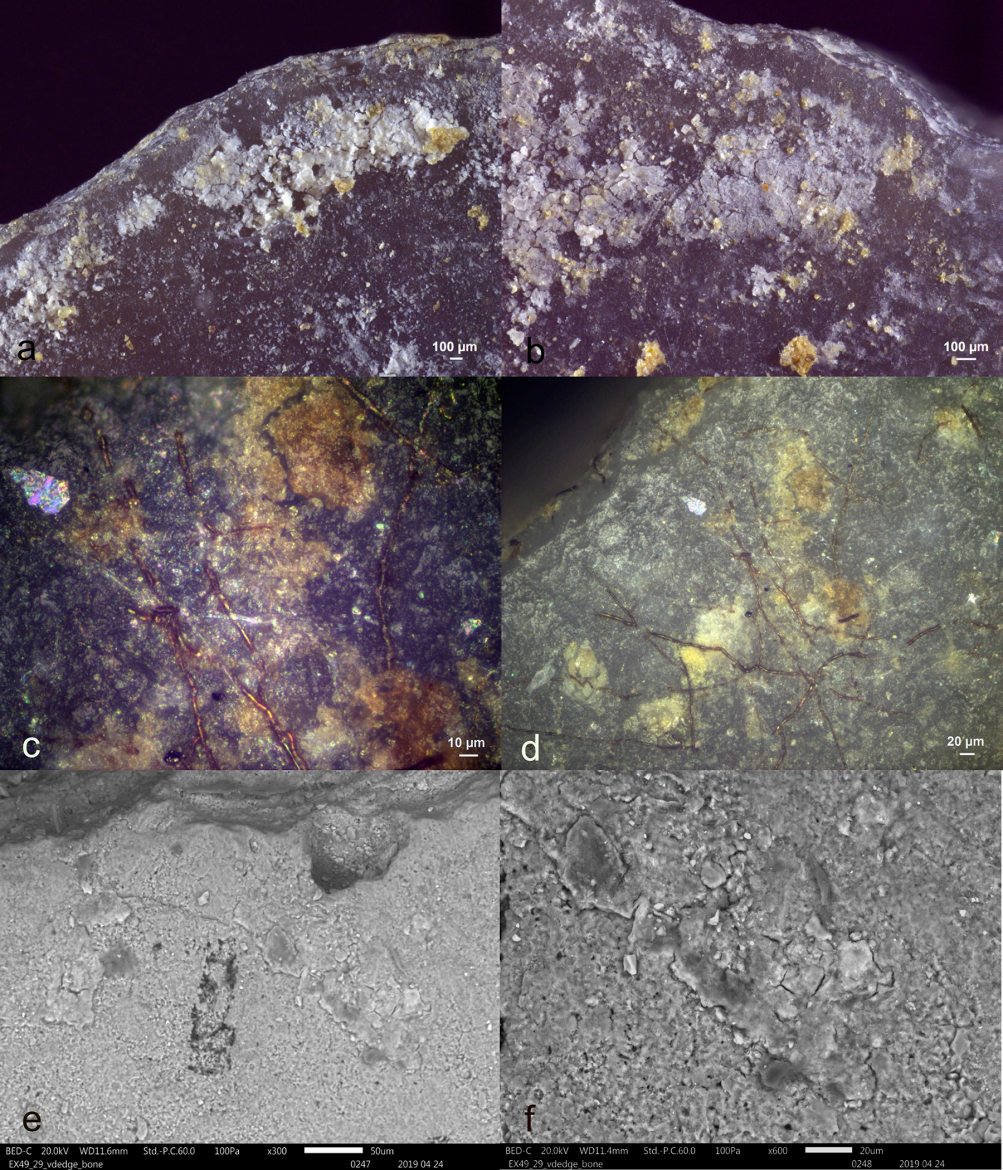
Visual characteristics of bone scraping residues after three-year burial: Strong association with the used edge (a+b+c+e), a significant degree of smearing (a,b), perpendicular directionality against the used edge (a+e), flat topography (e+f), brown discolouration (c+d), sharp edges and internal cracks (f)) (a = x54; b = x85; c = x500; d = x200; e = x300; f = x600).

Residues from bone scraping (Fig 29) were identified on four of the eight bone processing tools (EXP49/26, EXP49/29, EXP49/36, and EXP49/50). Their identification was based on their plate-like structure/cracked appearance, particularly under cross-polarized light. While other mineral residue types may also display similar visual characteristics, this might considered a first identification in recognizing bone residues [91] This identification was further substantiated by an EDS analysis, confirming the presence of Calcium and Phosphorus in these residual deposits, suggesting bone scraping residues. The bone densities exhibited variation, ranging from isolated, small deposits (rated as d1) to continuous and thick accumulations. Most bone deposits were predominantly situated on the ventral surface close to the used edge, which greatly facilitated the identification of the residue’s origin. In cases where the deposits were thick and dense, they were characterized by a strong degree of smearing and a perpendicular alignment against the utilized edge.

**Fig 30:**
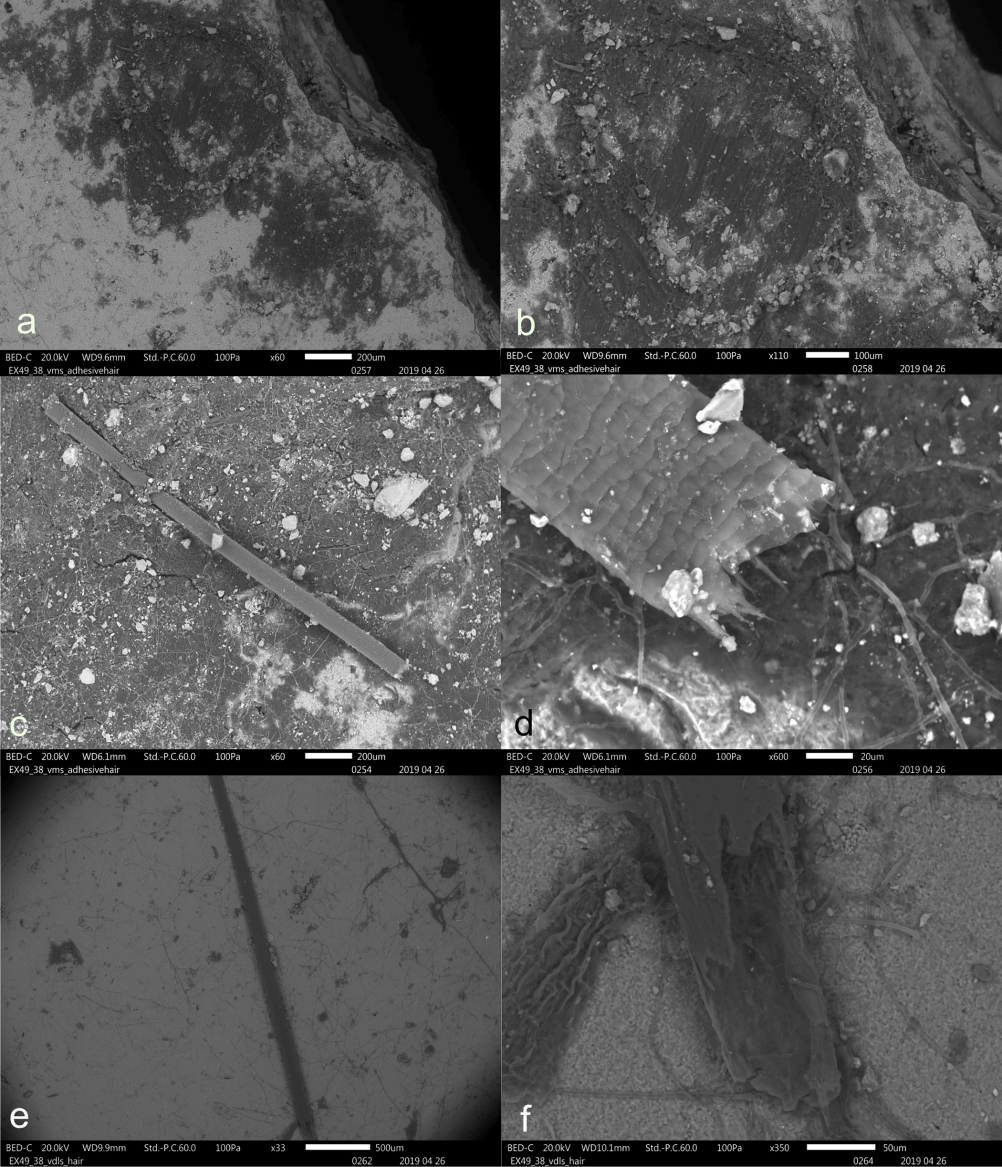
Visual characteristics of hide scraping residues on tool 49/38 (Lommel) after three-year burial: Strong association with the used edge (a+b), a significant degree of smearing (a,b), perpendicular directionality against the used edge (a+b), amorphous fat deposits (a+b+c), isolated hairs (c+e) with intact cuticles (d) and degraded hair shaft (f)) (a = x60; b = x110; c = x60; d = x600; e = x33; f = x350).

Residues from fresh hide scraping were only detected on three of the six hide scrapers (EXP49/37 and EXP49/38). These residues primarily comprised smeared fat deposits strongly associated with the utilized edge. These distinguishing characteristics allowed for a confident identification of the residue’s origin. The fat deposits were exceptionally well-preserved on Exp49/38, (**Fig 30**), while they were less prominently retained on Exp49/37, with only isolated patches of fat deposits visible. These fat deposits were visible under stereo- and incident-light microscopy; however, precise identification remained challenging due to their translucent and amorphous appearance.

Further examination with a scanning electron microscope revealed the deposits’ pure organic composition. On both tools, isolated hair fragments were identified, with Exp49/37 displaying signs of erosion at the hair shaft, likely due to fungal activity. Interestingly, the hide scraping tools exhibited a notably higher fungal density on the active part compared to tools used for other activities.

**Fig 31:**
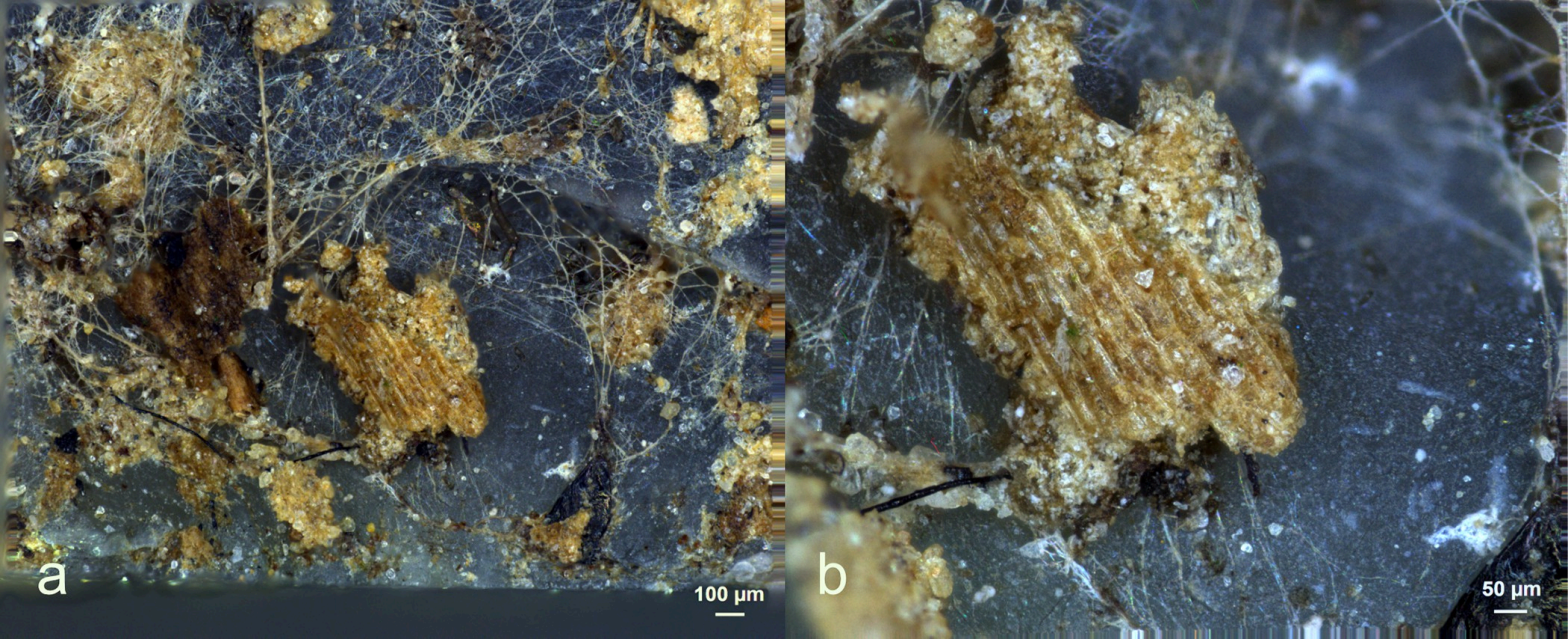
Visual characteristics of soft plant scraping residues on tool 49/14 (Rochefort) after three-year burial: Intermediate association with the used edge, absent degree of smearing, absent directionality against the used edge, intact plant cells (b), fungal growth (a)) (a = x79; b = x180).

Residues from soft plant (*Raphanus sativus longipinnatus*) scraping (**Fig 31**) were identified on three out of the six soft plant scrapers (EXP49/13, EXP49/14, and EXP49/17). These residues appeared as patches of plant tissue located near the utilized edge, oriented perpendicularly to the edge, with no visible smearing. The soft plant residues were primarily plant tissue, featuring visibly intact plant cells. However, they lacked any further distinctive characteristics, allowing their differentiation from wood tissue.

**Fig 32:**
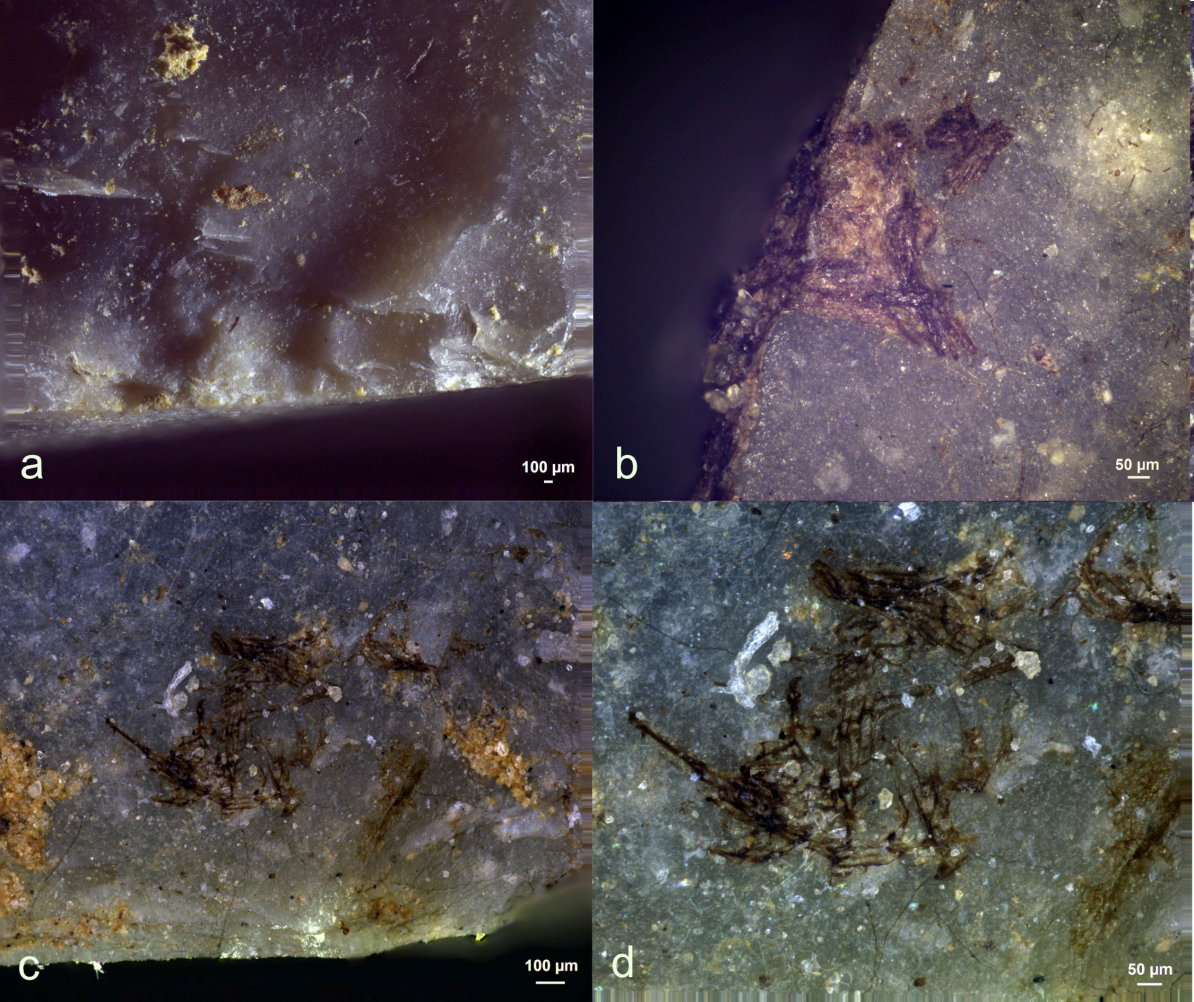
Visual characteristics of wood scraping residues after three-year burial: General poor densities (a) strong association with the used edge (a+c), no smearing (b+c+d), visible plant cells (d) a = Exp49/12 (Scladina); b = Exp49/55 (Lommel); c+d = exp49/52 (Rochefort)) (a = x31.5; b = x100; c = x104; d = x180).

Residues from wood scraping (**Fig 32**) were found on five of the six wood scrapers (EXP49/12, EXP49/51, EXP49/52, EXP49/55, and EXP49/56). However, it is essential to note that there was a significant residue loss. All wood deposits displayed a strong connection with the utilized edge, exhibited various directionalities concerning the used edge, and were notably devoid of visible smearing (**Table 10**). The absence of smearing made it challenging to pinpoint the precise cause of the residue. Furthermore, identifying wood residues primarily relied on the presence of intact cells, which provided a reliable means of recognition.

The observed environmental residues encompassed authigenic calcium, hyphae, and rootlets. In most instances, the calcium manifested as white micro-crystalline deposits when viewed under stereo and bright-field incident light microscopy, rendering it indistinguishable from bone minerals. However, EDS analysis proved instrumental in differentiating between calcium and bone minerals, revealing the distinct pure Calcium composition. Hyphae were present on all stone tools, although their densities exhibited variations contingent on the associated residue type. The adhesive deposits harboured the densest hyphal presence, closely followed by the hide-scraping deposits. Notably, hyphae displayed their greatest density in regions where residues were present.

Additionally, rootlet tissues were identified on several stone tools and could be discerned by their characteristic round, hollow structure and intact epidermal tissue.

#### Factors that influenced the effect of burial processes

The results indicate that both **the residue type** and the **depositional environment’s conditions** are important factors in determining the effect of burial on stone tool residues. The *loss of residues* appears to be heavily influenced by the residue’s ability to withstand burial processes, primarily governed by microbial activity. In this context, the resin and beeswax mixture was the most resilient residue type, with all its deposits enduring regardless of the depositional environment (**Table 11**).

**Table 11:** Observed densities and loss for the resin/beeswax residues.

*Tool Id*	*Depositional environment*	*Observed density*	*Loss*
*EXP49/12*	Scladina	4	absent
*EXP49/13*	Rochefort	4	absent
*EXP49/18*	Lommel	4	absent
*EXP49/38*	Lommel	4	absent
*EXP49/42*	Rochefort	4	absent
*EXP49/48*	Val-Meer	4	absent
*EXP49/52*	Rochefort	4	absent
*EXP49/56*	Lommel	4	absent

*Wood scraping residues* were the second most resilient since five of the six wood scrapers (83%) still contained wood residue (**Table 12**). The only wood scraper without residues was buried in the loess soil of Val-Meer, suggesting that this depositional environment might be extremely hostile to wood residues.

**Table 12:** Observed densities and loss for wood scraping residues.

*Tool Id*	*Depositional environment*	*Observed Density*	*Loss*
*EXP49/11*	Val-Meer	0	complete
*EXP49/12*	Scladina	1	strong
*EXP49/51*	Rochefort	2	strong
*EXP49/52*	Rochefort	2	strong
*EXP49/55*	Lommel	3	weak

*Bone scraping* residues have been observed on four of the eight buried tools (50%), and their preservation seems to be strongly linked with the pH conditions of the depositional environment. The bone residues were not preserved within the most acidic soil, the loess soil from Val-Meer. In contrast, those exposed to the most basic soil, the clayey soil of Scladina cave, showed no or minimal loss and retained their original high bone densities (**Table 13**). The bone scraping residues buried in Rochefort and Lommel, both acidic soils, showed intermediate preservation as bone preserved on one tool of two buried tools.

**Table 13:** Observed densities and loss for bone scraping residues.

*Tool Id*	*Depositional Environment*	*Observed density*	*Loss*
*EXP49/25*	Lommel	0	Complete
*EXP49/26*	Lommel	1	Strong
*EXP49/29*	Rochefort	1	Strong
*EXP49/30*	Rochefort	0	Complete
*EXP49/36*	Scladina	4	Weak
*EXP49/50*	Scladina	4	Absent
*EXP49/35*	Val-Meer	0	Complete
*EXP49/49*	Val-Meer	0	Complete

*Plant scraping* residues were observed on three of the seven buried tools (43%), preserving particularly well within the soil of the forest environments of Lommel and Rochefort (**Table 14**). It remains unclear why this is the case.

**Table 14:** Observed densities and loss for plant scraping residues.

*Tool Id*	*Depositional environment*	*Observed density*	*Loss*
*EXP49/13*	Rochefort	1	Strong
*EXP49/14*	Rochefort	3	Intermediate
*EXP49/17*	Lommel	2	Intermediate
*EXP49/18*	Lommel	0	Complete
*EXP49/23*	Scladina	0	Complete
*EXP49/49*	Val-Meer	0	Complete
*EXP49/50*	Scladina	0	Complete

*Fat* from hide scraping was considered the least resistant residue within these temperate depositional environments, as only two of the six hide scraping tools (33%) preserved residues (**Table 15**). It is unclear Observed densities and loss for hide scraping residues.why the sandy soil of Lommel proved to be the only environment suitable for preserving fats and hair.

**Table 15:** Observed densities and loss for hide scraping residues.

*ToolId*	*Depositional environment*	*Observed Density*	*Loss*
*EXP49/37*	Lommel	1	strong
*EXP49/38*	Lommel	3	intermediate
*EXP49/41*	Rochefort	0	complete
*EXP49/42*	Rochefort	0	complete
*EXP49/47*	Scladina	0	complete
*EXP49/48*	Val-Meer	0	complete

Over the three-year burial period, most residual deposits (56%) underwent colour changes, varying from a darkening of their initial hue to a complete alteration in colour. Colour change proved strongly linked with residue type and, to a lesser extent, with the depositional environment **(Table 16)**. Resin and resin with beeswax deposits appeared to be the most susceptible to colour change, with all deposits taking on a dark brown stain. In most cases, wood and soft plant tissue deposits also displayed a darker brown colour. The burial environment’s conditions also significantly influenced the colouration of bone residues. Two bone deposits from Rochefort and Lommel became translucent, while the two from Scladina retained their original white colour. The preservation of the white colour could be attributed to the high presence of carbonate in the cave sediment, whereas the soil in Lommel and Rochefort contained fewer carbonates.

**Table 16:** Properties of colour change for the observed residues.

*ToolId*	*Face*	*Nature*	*Cause*	*Location Deposition*	*Change in colour*	*Colour*
*EXP49/12*	Dorsal	Leather	Hafting	Scladina	no	dark brown
*EXP49/12*	Ventral	Leather	Hafting	Scladina	no	dark brown
*EXP49/12*	Ventral	Resin	Hafting	Scladina	yes	black
*EXP49/12*	Ventral	Wood	Use	Scladina	yes	dark brown
*EXP49/13*	Ventral	Resin	Hafting	Rochefort	yes	black
*EXP49/13*	Ventral	Plant	Use	Rochefort	yes	transparent
*EXP49/14*	Dorsal	Plant	Use	Rochefort	yes	brown
*EXP49/14*	Ventral	Plant	Use	Rochefort	yes	brown
*EXP49/17*	Dorsal	Plant	Use	Lommel	yes	brown
*EXP49/17*	Ventral	Plant	Use	Lommel	yes	brown
*EXP49/18*	Dorsal	Resin	Hafting	Lommel	yes	black
*EXP49/18*	Ventral	Resin	Hafting	Lommel	yes	black
*EXP49/26*	Ventral	Bone	Use	Lommel	no	white, yellow
*EXP49/29*	Dorsal	Bone	Use	Rochefort	no	white
*EXP49/29*	Ventral	Bone	Use	Rochefort	yes	white-brown
*EXP49/30*	Ventral	Resin& beeswax	Hafting	Rochefort	yes	brown
*EXP49/36*	Dorsal	Bone	Use	Scladina	no	white
*EXP49/36*	Ventral	Bone	Use	Scladina	no	white
*EXP49/37*	Dorsal	Fat	Use	Lommel	no	transparent
*EXP49/37*	Ventral	Fat	Use	Lommel	no	transparent
*EXP49/38*	Ventral	Resin	Hafting	Lommel	yes	black, remained yellow at bottom
*EXP49/38*	Dorsal	Fat	Use	Lommel	no	transparent
*EXP49/38*	Ventral	Fat	Use	Lommel	no	transparent
*EXP49/42*	Ventral	Resin	Hafting	Rochefort	yes	black
*EXP49/48*	Dorsal	Resin	Hafting	Val-Meer	yes	dark brown
*EXP49/48*	Ventral	Resin	Hafting	Val-Meer	yes	dark brown
*EXP49/48*	Ventral	Wood	Hafting	Val-Meer	no	brown
*EXP49/50*	Butt	Antler	Production	Scladina	no	white
*EXP49/50*	Dorsal	Bone	Use	Scladina	no	white
*EXP49/50*	Ventral	Bone	Use	Scladina	no	white
*EXP49/50*	Ventral	Plant	Use	Scladina	no	brown
*EXP49/51*	Dorsal	Wood	Use	Rochefort	yes	dark brown
*EXP49/52*	Ventral	Resin	Hafting	Rochefort	yes	brown
*EXP49/52*	Ventral	Wood	Use	Rochefort	no	brown
*EXP49/55*	Dorsal	Wood	Use	Lommel	yes	dark brown
*EXP49/55*	Ventral	Wood	Use	Lommel	yes	dark brown
*EXP49/56*	Ventral	Resin	Hafting	Lommel	yes	dark brown
*EXP49/56*	Dorsal	Wood	Use	Lommel	yes	dark brown
*EXP49/56*	Ventral	Wood	Use	Lommel	yes	dark brown

#### Factors that influenced the effects of weathering

The results showed that both the **residue type** and **the environmental conditions during deposition** played pivotal roles in determining the extent of weathering impact (**Table 17**). Specifically, the resilience of a residue against prevailing environmental conditions largely dictated the degree of residue loss. In this context, it was notable that the soil surface conditions were predominantly acidic. The pH level in Lommel was exceptionally low at 3.3, whereas Rochefort’s pH registered only mildly acidic at 6.2. Hide residues resulting from hide scraping exhibited remarkable resistance to the weathering processes they endured, with most of these residues displaying minimal loss. This starkly contrasted with the bone residues, which were only preserved on the distal ventral left edge of tool EXP49/31 deposited on the soil surface in Rochefort.

**Table 17:** Observed densities and loss for the wood scraping residues.

*Tool Id*	*Nature*	*Location Deposition*	*Observed Density*	*Loss*
*EXP49/07*	Wood	Lommel	2	strong
*EXP49/08*	Wood	Lommel	2	strong
*EXP49/53*	Wood	Rochefort	0	complete
*EXP49/54*	Wood	Rochefort	0	complete
*EXP49/15*	Plant	Rochefort	2	intermediate
*EXP49/16*	Plant	Rochefort	3	intermediate
*EXP49/19*	Plant	Lommel	0	complete
*EXP49/20*	Plant	Lommel	1	strong
*EXP49/27*	Bone	Lommel	0	complete
*EXP49/28*	Bone	Lommel	0	complete
*EXP49/31*	Bone	Rochefort	2	intermediate
*EXP49/32*	Bone	Rochefort	0	complete
*EXP49/39*	Fat	Lommel	3	weak
*EXP49/40*	Fat	Lommel	2	intermediate
*EXP49/43*	Fat	Rochefort	3	weak
*EXP49/44*	Fat	Rochefort	3	weak

A thorough examination with the scanning electron microscope confirmed the absence of bone residues on the other stone tools (EXP49/27, EXP49/28, EXP49/32). The limited preservation of bone could be attributed to the elevated soil acidity, which induced the dissolution of bone minerals.

**Fig 33:**
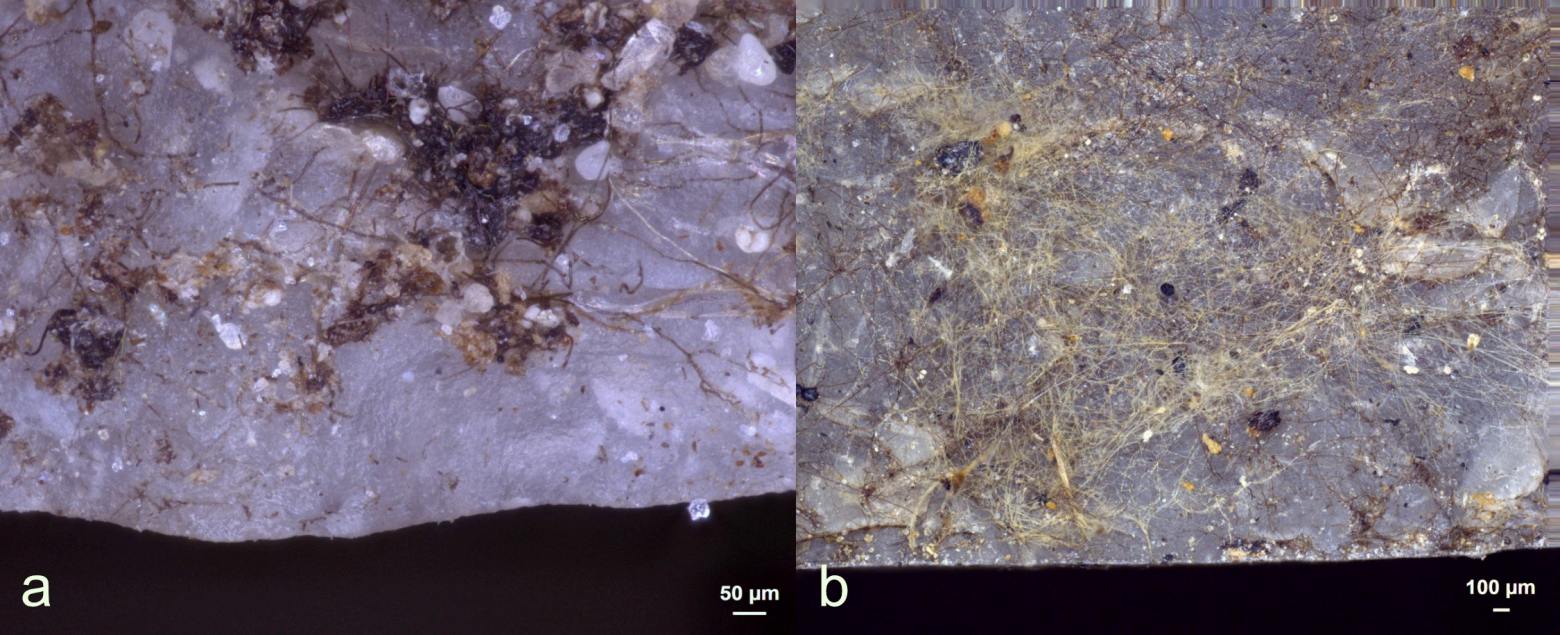
Differential fungal growth and wood preservation (a) Less intense fungal growth (d2) on distal dorsal end of tool Exp49/08 that was deposited on Lommel soil surface (b) Intense fungal growth (d4) on distal dorsal end tool Exp49/53 that was deposited on the Rochefort soil surface) (a = x65; b = x45.5).

The **depositional environment**, and more specifically, the pH conditions of the topsoil, emerged as a crucial factor in determining residue loss. Wood residues, for instance, were only preserved on the scrapers from Lommel (EXP49/07; EXP49/08), albeit in relatively low densities. The distinct preservation outcomes between the two locations can be attributed primarily to variations in fungal growth patterns (**Fig 33**). The soil of Lommel, characterized by its extreme acidity, acted as an inhibitor of fungal growth, resulting in better residue preservation compared to the scrapers deposited at Rochefort.

In the case of soft plant residues, higher residue densities were observed on the Rochefort scrapers (EXP49/15; EXP49/16) compared to the Lommel scrapers (EXP49/19; EXP49/20), which displayed significant fragmentation. Interestingly, this observation contradicts the more intense fungal growth on both Rochefort scrapers and the cause of this disparity remains unclear.

## Discussion

In recent years, the study of stone tool residues has yielded unique and groundbreaking insights into past human behaviour [118–123], enabling us to achieve a level of detail about stone tool usage that had been unattainable through other methods. Residue analysis has become an essential component of functional analysis, however, relatively little attention has been dedicated to studying the effects of post-depositional processes on stone tool residues [42–44]. Factors influencing the preservation or degradation of residues remain largely unknown, which contrasts with other archaeological disciplines, such as archaeozoology, where mechanisms for preservation and degradation are better understood [10, 88, 124, 125]. Enhance our comprehension of residue preservation requires gather more empirical data through experimentation and examining archaeological lithics and refining our understanding of residue taphonomy to also more accurately assess the potential of an archaeological context.

It is generally assumed that stone tool residues preserve poorly and that they mostly completely degrade within temperate contexts [32]. This assumption relies on either limited experimentation [42, 44] or knowledge acquired from the taphonomy of macro remains [11, 19, 88], but nevertheless resulted in few residue studies having been conducted on such contexts, potentially overlooking valuable information. Our study aimed to evaluate the preservation potential of stone tool residues within temperate contexts through comprehensive and large-scale actualistic experiments and systematic tests of the impact of different variables. The combination of three experiments allowed us to examine the effect of both weathering and burial processes on residue preservation and interpretation and because bothprocesses are associated with different taphonomic agents [51], we first studied these processes separately after which we assessed their combined effect.

Weathering, in particular subaerial weathering, is widely acknowledged as one of the most destructive post-depositional processes for archaeological materials [5, 19, 126–129] however, only limited attention has been given to its impact on stone tool residues [42]. The one-year monitoring experiment provided valuable insights into how each residue type reacts when exposed to weathering and highlighted varying resistance and significant difference in residue loss. Soft animal residues rapidly decayed with most residue having deteriorated within two weeks after being placed on the soil surface. This swift decay is likely due to intense microbial activity as commonly observed in soils of temperate environments [129, 130].

In contrast, wood residues appeared less susceptible to microbial activity and exhibited slower decay. One potential explanation could be that the compressed wood residues hinder enzyme penetration and restrict the extensive breakdown of hemicellulose and cellulose [130]. Mechanical weathering played a more prominent role and caused residues to tear off, especially during the initial weeks of exposure. The orientation of the tool proved influential with wood residues on the upward-facing surface and fully exposed to mechanical weathering being strongly affected and those on the downward-facing surface being shielded from such processes and preserving better. The impact of short-term weathering was less pronounced for other residue types, notably the resin/beeswax mixture, which exhibited exceptional resistance to weathering due to the strong adhesive [131] and antifungal properties [132–134] of terpene and terpenoid molecules. This resilience has been observed [135] and partly explains why they may preserve on assemblages from open-air sites [136, 137]. It suggests that similar residue types (e.g., birch tar) have a high chance of survival on stone tools from assemblages derived from temperate open-air sites. More systematic screening of such assemblages for these resilient and water-insoluble residue types may thus prove useful, even if stone tools have been cleaned [96, 135, 138].

The three-year surface experiment further validated distinct preservation patterns for the different residue types when exposed to weathering. Once again, the findings indicate that the residues’ unique resistance against specific weathering conditions determines the outcome. Bone residues exhibited poor preservation compared to other residue types, suggesting that chemical weathering played a significant role in their loss. The low pH values in the Lommel and Rochefort locations contributed to the partial dissolution of bone minerals, as these burial conditions are renowned for destroying bone [110, 139, 140]. At the same time, other residue types (such as resin/beeswax and hide) displayed greater resilience to chemical weathering, particularly for hide scraping residues.

Preservation clearly differed between the two locations and can be attributed to the notable disparity in soil acidity in each area. The long-term preservation potential of hide residues within acidic conditions requires further investigation following the results from other burial settings [123]. The experiment confirms that weathering in temperate environments results in substantial loss of residues [32], contingent upon the residue type, but none of the residue types completely vanished demonstrating that residues may even preserve in unfavourable environments. Our findings also indicate that impact from weathering is most pronounced during the initial weeks after exposure, and gradually diminishes over time, at the exception of chemical weathering.

We also showed that weathering introduces complexities for the visual identification of stone tool residues, because colour changes are frequent. For instance, white bone residues may become translucent, while translucent fat can turn brown. This observation casts doubt on the reliability of using colour as an argument for identifying amorphous residues [54, 141–143], as this visual property appears unstable. Browned fat can easily be mistaken for hafting adhesive such as resin based solely on visual observations, thus confirming the important role of using molecular characterization techniques like Raman, GC-MS or FTIR for accurate identification [81, 85, 117, 122]. Additionally, our experiments highlighted that weathering also significantly impacts the accurate interpretation of the process that deposited the residue because the initial distribution pattern is disrupted and significant residue loss may have resulted in sparse densities. Deposition of environmental residues, such as bird feathers or wood remains from humus, may further increase the risk of misinterpretation. It underscores the importance of integrating evidence from both use-wear and residue analysis, as demonstrated previously [78, 90, 144, 145], and raises questions about some studies that have reported such residue types without providing corresponding use-wear traces [146–148].

A burial of three years proved to lead to considerable residue loss and a modification in residue colour. Although most residual deposits were significantly affected, the characteristics used to recognize the deposition process generally remained unaffected. For instance, the remaining use residues remained associated with the used edge, which helped identifying the deposition process. However, butchering residues (muscle tissue, collagen) were not included in this study and previous experiments have indicated substantial loss of these residue types upon burial, up to the point that also the criteria used for recognizing the deposition process are affected [42, 44]. Our experiments further showed that burial resulted in the deposition of environmental residues (wood, calcium minerals), which could potentially be mistaken for functional residues if not considered.

The overall outcome of this taphonomic experiment affirmed the existence of specific patterns of residue preservation within temperate environments, which complements findings from previous experiments conducted in different settings [42, 44]. Drawing from both prior knowledge and evidence presented here, the impact of each post-depositional process on a residue proves contingent upon its characteristics, such as whether it is biological, physical, or chemical in nature, its intensity, and the specific sequence in which processes occur, a concept known as non-commutativity [52]. It further depends on the resistance exhibited by the residue in the face of these processes, which is strongly influenced by the structural properties of the biomolecules [93, 94]. As the depositional environment determines the nature, intensity and sequential order of the post-depositional processes [92], it is crucial to study the effect of each depositional environment on stone tool residues. In the case of temperate environments, our study further confirmed the results of an earlier experimental study by Croft et al. [44] that fungal activity and soil acidity are the primary factors responsible for residue decay. -The important role of fungal activity and soil acidity is best illustrated in our study by the fact that the depositional environment with the highest density of hyphae on the remaining residues and lowest pH value (5.95, Val-Meer) showed the poorest residue preservation.

Consequently, residue types most resistant to microbial activity and soil acidity survived best. Resin/beeswax, for instance, appears indestructible under short-term taphonomic impact, as previously observed [135], while bone and fat residues seem highly vulnerable in temperate conditions. The chemical stability of lipid-based residues such as resin and beeswax over long periods is primarily due to their saturated chemical bonds, hydrophobic nature, oxidative stability, protective environmental properties, and potential for polymerization [93, 94, 149]. These factors collectively contribute to their resistance to degradation and longevity in various conditions. In the case of bone residues, soil acidity plays a major role in their decay, as highly acidic conditions lead to complete bone mineral dissolution, as observed with faunal remains [53, 125, 150, 151]. Soft animal residues such as collagen or muscle tissue seem to preserve very poorly within open-air temperate conditions, which aligns with findings from previous studies on the decay of soft tissue from different environmental contexts [42, 44, 55, 56]. The vulnerable character of these residue types, particularly against microbial activity, raises questions regarding the frequent identification of such residue types (e.g., blood, collagen, muscle tissue) on stone tools [57, 62, 63, 146]. From a molecular perspective, unprotected proteins (i.e., not preserved within a mineral matrix such as bone or shell) show a low preservation potential. They are generally assumed to be preserved under exceptional conditions [94]. The preservation of such fragile residue types on stone tools is controversial and biochemical methods may also lead to false-positive results, such as the false identification of blood through the use of Hemastix [152–156]. In addition, recent research has revealed that modern proteinaceous residues may deposit easily on stone tools during (post-) excavational handling [49, 96, 157], demonstrating the necessity implementing procedures that distinguish modern from ancient proteinaceous residues, such as screening with a DAPI fluorescence filter [118]. Such precautions were not taken in the studies mentioned above, and further confirmation is therefore required before these results can be considered reliable.

Further experimentation and analysis of archaeological residues are necessary to further improve insight into the long-term effects of post-depositional processes on stone tool residues. Ideally, such experiments should incorporate a range of residue types and depositional environments. Residue studies performed on archaeological assemblages from open-air temperate contexts seem to corroborate our findings that only the most resilient residue types, such as vegetal or mineral residues, preserve within these settings [138, 158]. In such cases, the residues yielded unique and precise insights that enhanced comprehension of how stone tools were utilized, including identifying the use of hafting adhesives. Such archaeological evidence, combined with findings from the experiments presented here, underscore the potential of open-air contexts in temperate environments, though differential preservation will always need to be considered. In any case, researchers should be encouraged to investigate these sites for residues rather than disregard them.

## Conclusion

Our study assessed the preservation potential of residues on stone tool in temperate environments, which are often considered unfavourable. Analysing residues from experimental stone tools exposed to weathering and burial provided new insights into the taphonomy of stone tool residues under such harsh conditions. Both exposure to weathering and burial resulted in significant degradation of most residue types, although many proved to be more resilient than previously believed. For the majority of residual deposits, the visual characteristics that can link the residue with its deposition process (i.e. smearing, association with the used edge) remained visible, even after three years of burial, except for the butchering residues. Furthermore, the wide array of tested depositional environments allowed us to observe that the preservation potential of residues is influenced by the specific conditions of the depositional environment and the resilience of the residue types to these conditions. Microbial activity and soil acidity significantly impacted residue preservation, and but also other factors contributed to the degradation or survival of residues in these environments. Certain residue types, such as resin, exhibited remarkable resilience when exposed to post-depositional processes, suggesting their high potential for preservation in temperate environments. Considering the preservation potential exhibited by specific residue types, researchers should be actively encouraged to explore these sites for residues rather than dismissing them outright. Our findings further revealed the complexity of residue taphonomy, emphasizing the need for additional experiments encompassing a broader spectrum of residue types and depositional conditions.

## Supporting information

S1 File Details of experimental setup. (DOCX)

S2 File Meteorological data from the weather station in Uccle during the period of the experiment. (XLSX)
