# Supplementary material for: Examining the effect of post-depositional processes on the preservation and identification of stone tool residues from temperate environments: An experimental approach
Source: PLoS One. 2024 Oct 21;19(10):e0309060. doi: 10.1371/journal.pone.0309060 (PMC11493299; doi:10.1371/journal.pone.0309060)
Supplement: S1 File — (DOCX) [file pone.0309060.s001.docx]

# SupplemENTARY INFORMATION 1: Experimental SEttings

# Experimental settings One-year weathering experiment

| N° | Code | Experimental variables | Categories |
| --- | --- | --- | --- |
| 1 | RM | Raw Material | flint (Harmignies) |
| 2 | AD | Adhesive recipe | Picea abies resin & beeswax (70-30%) |
| 3 | HA | **Handle** | bone; wood |
| 4 | MW | **Material worked** | fresh meat and bone (Sus scrofa domesticus); wood (Corylus avellana) |
| 5 | UM | Use Motion | cutting |
| 6 | UD | Use Duration | 20 minutes |
| 7 | EN | Environment | pine forest (Tongeren) |
| 8 | ET | Exposure time | between 2-46 weeks (2-week intervals) |

Table 1 Overview of the experimental variables used in the experiment.


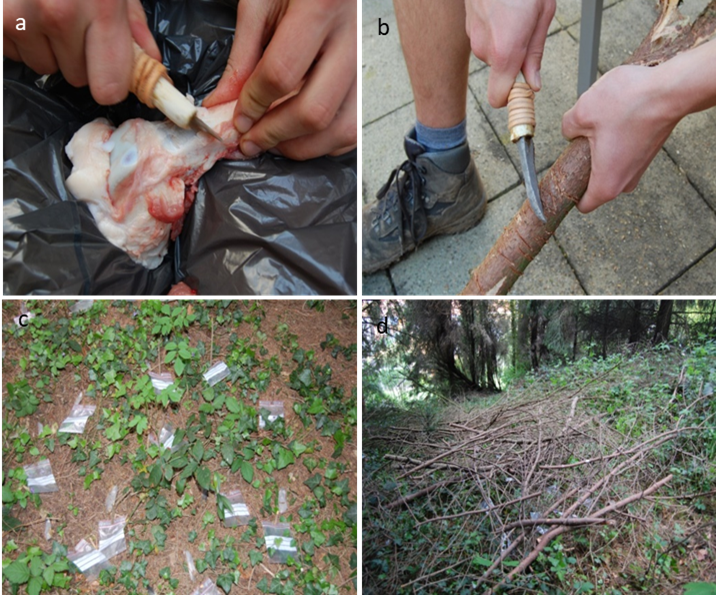


**Figure 1 Overview of the tool use activities and the depositional environment a) Removal of fresh meat from pig bone b) Wood cutting c) Depositional environment d) Covering of tools with pine tree branches to protect against carnivores**

| Depositional environment | |
| --- | --- |
| Location | Tongeren |
| N° tools | 44 |
| Environment | Pine forest |
| Coordinates | 50°10'01.6"N 5°15'24.2"E |
| Precipitation per year | 140-145 mm |
| Sediment | Purely organic: pine needles |
| Humidity | Humid |
| Soil pH | 6.2 |
| Bioturbation | High |
| Biological activity | High |

Table 2 Properties of the depositional environment

| Date | Fresh wood use | Fresh bone use | Weeks on surface |
| --- | --- | --- | --- |
| 07/05/2016 | Tools deposited on the soil | | |
| 21/05/2016 | EXP49/141 | EXP49/165 | 2 |
| 04/06/2016 | EXP49/163 | EXP49/172 | 4 |
| 18/06/2016 | EXP49/160 | EXP49/175 | 6 |
| 02/07/2016 | EXP49/168 | EXP49/151 | 8 |
| 16/07/2016 | EXP49/149 | EXP49/134 | 10 |
| 30/07/2016 | Exp49/173 | Exp49/205 | 12 |
| 13/08/2016 | EXP49/177 | EXP49/169 | 14 |
| 27/08/2016 | Exp49/144 | Exp49/155 | 16 |
| 10/09/2016 | Exp49/136 | Exp49/161 | 18 |
| 24/09/2016 | Exp49/157 | Exp49/171 | 20 |
| 08/10/2016 | Exp49/174 | Exp49/152 | 22 |
| 22/10/2016 | Exp49/146 | Exp49/170 | 24 |
| 05/11/2016 | Exp49/139 | Exp49/150 | 26 |
| 19/11/2016 | Exp49/135 | Exp49/162 | 28 |
| 03/12/2016 | Exp49/153 | Exp49/137 | 30 |
| 17/12/2016 | Exp49/159 | EXP49/180 | 32 |
| 31/12/2016 | Exp49/132 | Exp49/142 | 34 |
| 14/01/2017 | Exp49/154 | Exp49/167 | 36 |
| 28/01/2017 | Exp49/158 | Exp49/138 | 38 |
| 11/02/2017 | Exp49/145 | Exp49/148 | 40 |
| 25/02/2017 | Exp49/164 | Exp49/132 | 42 |
| 11/03/2017 | Exp49/178 | (lost) | 44 |
| 25/03/2017 | Exp49/206(182) | (lost) | 46 |

Table 3 Exposure time of the different stone tools

# Experimental settings: Three-year surface experiment

| N° | Code | Experimental variables | Categories |
| --- | --- | --- | --- |
| 1 | RM | Raw Material | flint (Harmignies) |
| 2 | AD | Adhesive recipe | *Picea abies* resin & beeswax (70-30%) |
| 3 | PM | **Prehensile mode** | handheld/hafted |
| 4 | HM | **Handle material** | bone;wood |
| 5 | MW | **Material worked** | hardwood (*Acer platanoides*); soft plant (*Raphanus sativus longipinnatus*); dry bone; fresh hide; |
| 6 | UM | Use Motion | scraping (SC) |
| 7 | UD | Use Duration | 20 minutes |
| 8 | EN | **Environment** | Lommel (LO) mixed forest; Rochefort (RO) |
| 9 | ET | Exposure time | +-3 years |

Table 4 Overview of the experimental variables used in the experiment

| Tool ID | hammer material | material  worked | prehensile  mode | ht | hm | b | ad | environment |
| --- | --- | --- | --- | --- | --- | --- | --- | --- |
| EXP49/07 | antler | hw (f) | hh |  |  |  |  | Lommel |
| EXP49/08 | antler | hw (f) | h | j | b | l |  | Lommel |
| EXP49/15 | antler | sp(f) | hh |  |  |  |  | Rochefort |
| EXP49/16 | antler | sp(f) | h | j | b | l |  | Rochefort |
| EXP49/19 | antler | sp(f) | hh |  |  |  |  | Lommel |
| EXP49/20 | antler | sp(f) | h | j | b | l |  | Lommel |
| EXP49/27 | antler | b(d) | hh |  |  |  |  | Lommel |
| EXP49/28 | wood | b(d) | h | j | w | l |  | Lommel |
| EXP49/31 | antler | b(d) | hh |  |  |  |  | Rochefort |
| EXP49/32 | antler | b(d) | h | j | w | l |  | Rochefort |
| EXP49/39 | anterl | h(f) | hh |  |  |  |  | Lommel |
| EXP49/40 | antler | h(f) | h | j | w | l |  | Lommel |
| EXP49/43 | wood | h(f) | hh |  |  |  |  | Rochefort |
| EXP49/44 | wood | h(f) | h | j | w | l | rb | Rochefort |
| EXP49/53 | antler | hw (f) | hh |  |  |  |  | Rochefort |
| EXP49/54 | anter | hw (f) | h | j | w | l |  | Rochefort |

Table 5 Experimental settings for the sixteen scrapers


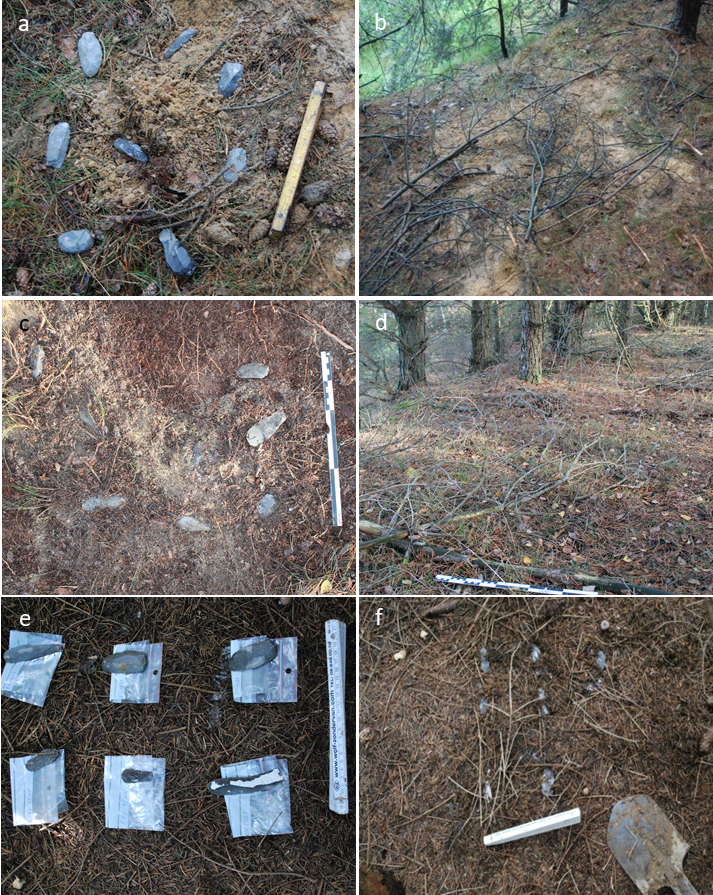


Figure 2Experimental settings for the 3-year weathering experiment a) Deposition of stone tools on the soil surface at Lommel b) Branches were placed over stone tools to minimize possible interventions from animals or humans c) Stone tools after three years d) Burial location after three years e) Detail of stone tools on the soil surface at Rochefort f) Deposition of stone tools on the soil surface at Rochefort

| Location | Rochefort | Lommel |
| --- | --- | --- |
| N° tools | 8 | 8 |
| Vegetation | Pine trees | Mixed wood forest |
| Coordinates | 50°10'01.6"N 5°15'24.2"E | 51°15'03.9"N 5°17'50.2"E |
| Precipitation per year | 140-145 mm | 140-145 mm |
| Soil properties | | |
| Sediment | Silt | Sand |
| Humidity | Humid | Humid |
| Soil pH | 6.4 | 3.3 |
| Biological activity | | |
| Bioturbation | high | high |
| Biological activity | high | high |

Table 6 Properties of the depositional environment

# Experimentals settings: Burial experiment

| N° | Code | Experimental variables | Categories |
| --- | --- | --- | --- |
| 1 | RM | Raw Material | flint (Harmignies) |
| 2 | MW | **Material worked** | Wood (Acer platanoides); Dry bone; Fresh hide; Fresh plant (Raphanus sativus longipinnatus) |
| 3 | PM | **Prehensile mode** | Haft; prehension |
| 4 | HA | **Handle material** | Wood (Corylus avelana); Bone (Capreolus, capreolus) |
| 5 | AD | **Adhesive** | Resin (70%) and beeswax (30%); none |
| 6 | ET | **Tool user** | Lode Cnuts; Dries Cnuts; Miel Cnuts; Daan Cnuts; Eugène Piette |
| 7 | UM | Use Motion | scraping |
| 8 | UD | Use Duration | 20 minutes |

Table 7 Overview of the experimental variables used in the experiment

| TOOL ID | MW | H/HH | HT | HA | B | AD | Location |
| --- | --- | --- | --- | --- | --- | --- | --- |
| EXP49/11 | HW(F) | HH | NA | NA | NA | NA | VM |
| EXP49/12 | HW(F) | H | J | B | L | RB | SCL |
| EXP49/13 | SP(F) | HH | NA | NA | NA | NA | RO |
| EXP49/14 | SP(F) | H | J | B | L | RB | RO |
| EXP49/17 | SP(F) | HH | NA | NA | NA | NA | LO |
| EXP49/18 | SP(F) | H | J | B | L | RB | LO |
| EXP49/23 | SP(F) | HH | NA | NA | NA | NA | SCL |
| EXP49/24 | SP(F) | H | J | B | L | NA | VM |
| EXP49/25 | B(D) | HH | NA | NA | NA | NA | LO |
| EXP49/26 | B(D) | H | J | W | L | RB | LO |
| EXP49/29 | B(D) | HH | NA | NA | NA | NA | RO |
| EXP49/30 | B(D) | H | J | W | L | RB | RO |
| EXP49/35 | B(D) | HH | NA | NA | NA | NA | VM |
| EXP49/36 | B(D) | H | J | W | L | NA | SCL |
| EXP49/37 | H(F) | HH | NA | NA | NA | NA | LO |
| EXP49/38 | H(F) | H | J | W | L | RB | LO |
| EXP49/41 | H(F) | HH | NA | NA | NA | NA | RO |
| EXP49/42 | H(F) | H | J | W | L | RB | RO |
| EXP49/47 | H(F) | HH | NA | NA | NA | NA | SCL |
| EXP49/48 | H(F) | H | J | W | L | RB | VM |
| EXP49/49 | B(D)+SP(F) | HH | NA | NA | NA | NA | VM |
| EXP49/50 | SP(F)+B(D) | H | J | B | L | NA | SCL |
| EXP49/51 | HW(F) | HH | NA | NA | NA | NA | RO |
| EXP49/52 | HW(F) | H | J | B | L | RB | RO |
| EXP49/55 | HW(F) | HH | NA | NA | NA | NA | LO |
| EXP49/56 | HW(F) | H | J | B | L | RB | LO |

Table 8 Experimental settings for the buried scrapers

| Experimental variables | Categories |
| --- | --- |
| Exposure time | 3 years |
| Sediment | Clay, Loess, Silt, Sand |
| Precipitation/year | 140-145 mm; 135-140 mm |
| Burial depth | 20 cm* (except scladina) |
| Vegatation | pine forest; wood forest; agricultural landscape; cave |

Table 9 General burial settings

|  | Rochefort | Lommel | Val-Meer | Scladina |
| --- | --- | --- | --- | --- |
| Deposition | 20 cm under the soil surface | 20 cm under the soil surface | 20 cm under the soil surface | Buried in tube |
| N° tools | 8 | 8 | 5 | 5 |
| Vegetation | Pine trees | Pine trees | Grasses | NA |
| Site type | Open-air | Open-air | Open-air | Cave |
| Coordinates | 50°10'01.6"N 5°15'24.2"E | 51°15'03.9"N 5°17'50.2"E | 50°47'40.7"N 5°35'02.4"E | 50°29'01.7"N 5°01'33.0"E |
| Precipitation/year | 140-145 mm | 140-145 mm | 135-140 mm | NA |
| Soil properties | | | | |
| Sediment | Silt | Sand | Loess | Clay |
| Soil Humidity | Humid | Humid | Humid | Dry |
| Soil acidity | 6.4 | 6.6 | 5.95 | 7.63 |
| Biological activity | | | | |
| Bioturbation | very high | high | low | NA |
| Biological activity | very high | high | low | NA |

Table 10 Detailed burial settings for the four different locations

# Metereological Data


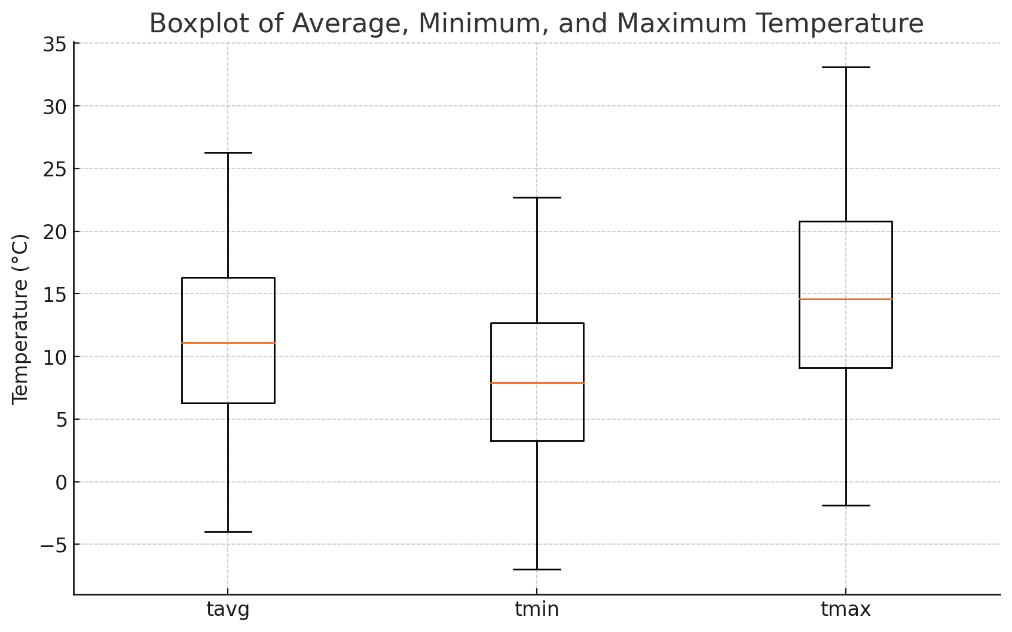


Figure 3 Boxplot of the average, minimum and maximum daily temperature during the period the experiment has taken place

-
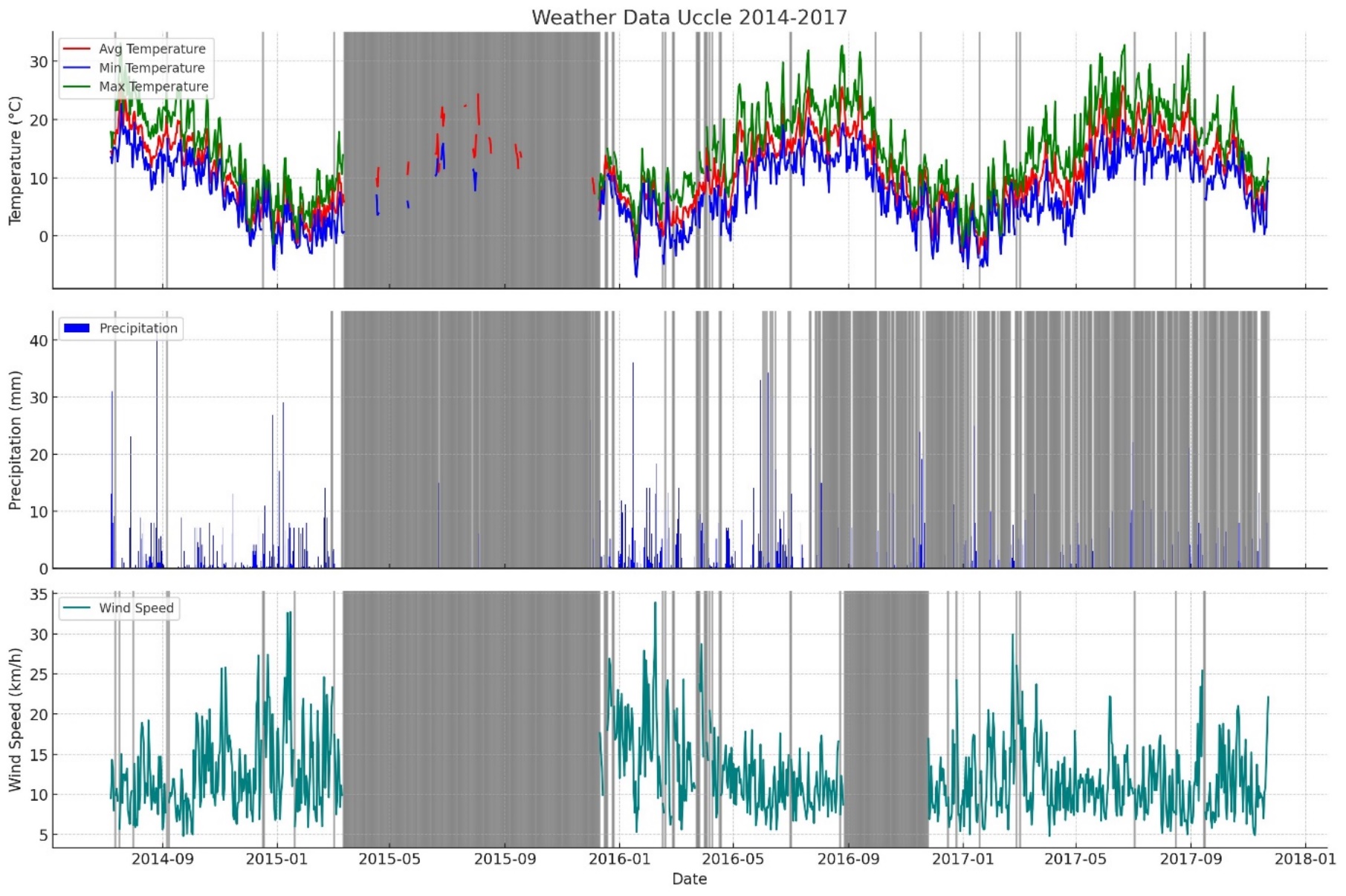

- Figure 4 The available meteorological data (temperature, precipitation, and windspeed) at Uccle (Brussels) from the Royal Meteorological Institute of Belgium during both the weathering and burial experiments are shown. Grey zones indicate the periods where no data is available
